# Insulin in motion: The A6-A11 disulfide bond allosterically modulates structural transitions required for insulin activity

**DOI:** 10.1038/s41598-017-16876-3

**Published:** 2017-12-08

**Authors:** Bianca van Lierop, Shee Chee Ong, Alessia Belgi, Carlie Delaine, Sofianos Andrikopoulos, Naomi L. Haworth, John G. Menting, Michael C. Lawrence, Andrea J. Robinson, Briony E. Forbes

**Affiliations:** 1 School of Chemistry, Monash University, Clayton, Victoria, 3800 Australia; 2 College of Medicine & Public Health, Flinders University of South Australia, Bedford Park, 5042 Australia; 3 University of Melbourne, Department of Medicine, Parkville, Victoria, 3010 Australia; 4 Research School of Chemistry, Australian National University, Acton, ACT 2601 Australia; 5 School of Life and Environmental Sciences, Deakin University, Waurn Ponds, Victoria, 3216 Australia; 6 The Walter and Eliza Hall Institute of Medical Research, 1G Royal Parade, Parkville, Victoria, 3052 Australia; 7 Department of Medical Biology, University of Melbourne, Royal Parade, Parkville, Victoria, 3050 Australia

## Abstract

The structural transitions required for insulin to activate its receptor and initiate regulation of glucose homeostasis are only partly understood. Here, using ring-closing metathesis, we substitute the A6-A11 disulfide bond of insulin with a rigid, non-reducible dicarba linkage, yielding two distinct stereo-isomers (*cis* and *trans*). Remarkably, only the *cis* isomer displays full insulin potency, rapidly lowering blood glucose in mice (even under insulin-resistant conditions). It also posseses reduced mitogenic activity *in vitro*. Further biophysical, crystallographic and molecular-dynamics analyses reveal that the A6-A11 bond configuration directly affects the conformational flexibility of insulin A-chain *N*-terminal helix, dictating insulin’s ability to engage its receptor. We reveal that in native insulin, contraction of the C_α_-C_α_ distance of the flexible A6-A11 cystine allows the A-chain *N*-terminal helix to unwind to a conformation that allows receptor engagement. This motion is also permitted in the *cis* isomer, with its shorter C_α_-C_α_ distance, but prevented in the extended *trans* analogue. These findings thus illuminate for the first time the allosteric role of the A6-A11 bond in mediating the transition of the hormone to an active conformation, significantly advancing our understanding of insulin action and opening up new avenues for the design of improved therapeutic analogues.

## Introduction

Insulin is fundamental to the physiological regulation of blood glucose concentration^1^. A deficiency in insulin results in diabetes, a major economic and primary health care burden across both developed and developing countries. Insulin therapy is essential in both type 1 diabetes and late-stage type 2 diabetes, with current therapeutic insulins being designed to restore the normal biphasic insulin response to food intake^2,3^. While such therapeutic analogues are largely successful in controlling blood glucose levels, their means of administration and their pharmacokinetic and pharmacodynamic profiles are far from ideal, putting patients at risk of both hyper- and hypoglycemia. Notably, none of the currently available therapeutic insulin analogues have employed in their design an atomic-level understanding of how insulin engages its receptor, as such detail has only recently begun to emerge^4,5^. A thorough understanding of the conformational changes involved in insulin/insulin receptor interaction therefore has the potential to lead to a new generation of insulin analogues with improved pharmacological properties.

**Figure 1:**
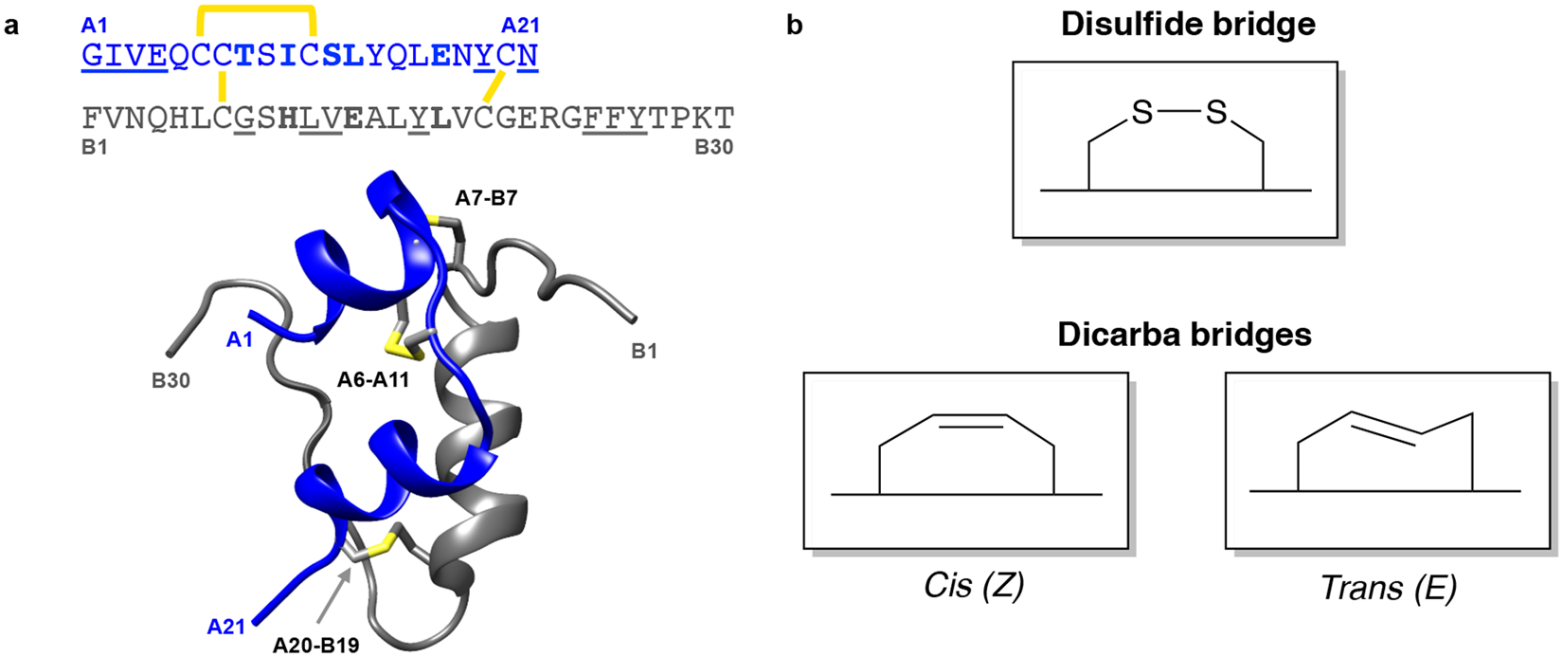
Insulin sequence and structure. (**a**) Primary sequence (*top*) of the A (*blue*) and B (*grey)* chains of human insulin, highlighting disulfide bonds (*yellow*), site 1-binding residues (*underlined*) and site 2-binding residues (*bold*)^14^. Ribbon diagram of insulin (2Zn-coordinated T_6_ conformation^16^ PDB entry 1MSO) showing the location of the three αhelices and the three disulfide bonds. (**b**) Schematic diagram of native cystine and isomeric *cis-* and *trans*dicarba bridges.

Insulin is a two-chain polypeptide, comprising an A chain of 21 residues that includes two α helices (residues A1 to A8 and A12 to A18, respectively), and a B chain of 30 residues that includes a single α helix (residues B9 to B19) (Fig. 1a)^6^. Integral to insulin’s structure are its three disulfide bonds — one intra-chain (Cys^A6^-Cys^A11^) and two inter-chain (Cys^A7^-Cys^B7^ and Cys^A20^-Cys^B19^) (Fig. 1a). Formation of these disulfide linkages ensures both the correct folding of the insulin precursor polypeptide and the structural stability of the mature hormone^7,8^. Both the A6-A11 and the A20-B19 cystines are buried within the core of the hormone, whereas the A7-B7 cystine is partly surface exposed. Insulin is stored as a 2Zn hexamer in pancreatic βcells, but it is the monomeric form that engages the insulin receptor (a receptor tyrosine kinase)^9^.

The insulin receptor is a disulfide-linked (αβ)_2_ homodimer, the ectodomain of which in its *apo* form adopts a folded-over Λ-shaped conformation^10,11^. Insulin binding to the insulin receptor is currently understood to involve the hormone forming a high-affinity cross-link between two distinct sites (1 and 2) on the receptor surface^4,12^. A number of the insulin residues involved in site 1 binding are also involved in forming the insulin dimer within the classical 2Zn insulin hexamer (Fig. 1a)^4,5,13,14^. The location of IR site 2 is not well defined^4,10^ but evidence exists that it is engaged by insulin residues involved in the hexamer-forming surface of the hormone^12,14^ (Fig. 1a).

Recent crystallographic studies of insulin in complex with domain-minimized insulin receptor constructs comprising site 1 alone revealed two key insights into the mechanism of interaction: (i) both the insulin B chain and the αCT segment of the receptor undergo conformational change upon their mutual engagement;^4,5^ in the case of insulin, such change involves the long-predicted folding out of the B-chain *C*-terminal segment (residues B24-B30) away from the hormone core^15^, and (ii) within the complex, the B-chain *N*-terminal segment does not form the *N*-terminal α-helical extension to the B8-B20 helix that is characteristic of the so-called R- or R^f^ states of the hormone that occur in crystals grown in the presence of phenolic derivatives^7^, but rather it adopts a conformation similar to that in the classical T-state structures of insulins^16^, wherein the B-chain *N*-terminal segment is folded back against the hormone^7^.

Lacking from our current understanding is the role of disulfide bond flexibility in insulin’s engagement with the IR. Here, we specifically seek to explore the influence of the A6-A11 disulfide bond on insulin structure and function through strategic use of olefin metathesis to replace the A6-A11 disulfide bond of insulin with a C=C double bond (Fig. 1b)^17^. An unsaturated C=C dicarba bond is considerably more rigid than a disulfide bond and adopts either a *cis* or *trans* configuration, with an insurmountable barrier to exchange under physiological conditions. Introduction of a dicarba bond into a number of small polypeptides^18^ (oxytocin^19^, calcitonin^20^, and H3-relaxin^21^) has been shown to improve their stability and, in some cases, their activity. Our synthetic techniques permit generation of both the *cis* and *trans* configuration of the A6-A11 dicarba bond within insulin (Fig. 1b). Taken together with a re-analysis of extant T-state insulin crystal structures, our structural, molecular dynamics and biological characterization of these dicarba insulin isomers leads to a new understanding of the critical interplay between A-chain conformational flexibility and restraint that is allosterically regulated by the A6-A11 disulfide bond. Such structural transitions are required for insulin/insulin receptor engagement.

## Results

### Chemical synthesis

**Figure 2:**
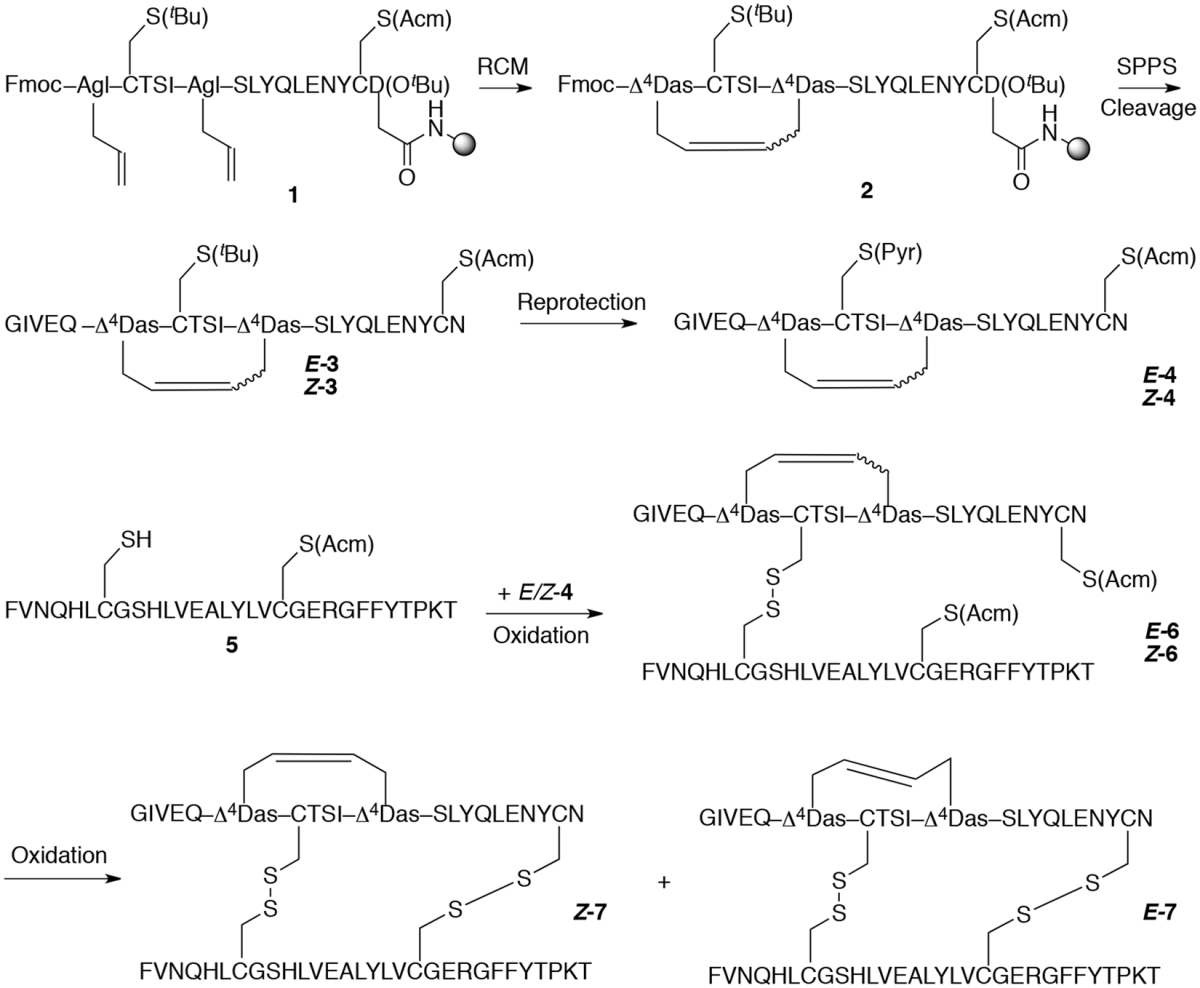
Synthesis of dicarba insulins was performed *via* ring-closing metathesis (RCM) and an interrupted solid phase peptide synthesis (SPPS)-catalysis approach. L-Allylglycine (Agl), *tert*-butyl (^*t*^Bu), acetamidomethyl (Acm), *S*-pyridinyl (Pyr), *cis* isomer (*Z*), *trans* isomer (*E*).

While dicarba bonds have been previously introduced into the analogous bond of insulin-like peptides INSL3 and INSL7^21–23^, no strategy exists in the literature to generate A6-A11 intra-chain dicarba analogues of human insulin. Here, the highly hydrophobic *N*-terminus of the insulin A-chain necessitated the development of an interrupted solid phase peptide synthesis (SPPS)-catalysis approach^24^ to overcome deleterious aggregation and achieve quantitative ring closing metathesis (*see Supplementary Methods*). Additionally, to ensure exclusive generation of the required *C*-terminal asparagine residue on resin cleavage, Fmoc-L-Asp-O^*t*^Bu was loaded onto Rink amide resin *via* its side chain. Microwave-accelerated SPPS in combination with HATU–DIPEA activation and Fmoc-protected amino acids were used to generate the truncated peptide sequence **1** (Fig. 2), carrying through each intermediate without purification and characterization. Two strategically placed L-allylglycine residues were incorporated into the primary sequence to facilitate formation of the intra-chain dicarba bridge, and cysteine residues were orthogonally protected to later aid regioselective disulfide oxidation and tethering of the B chain (Fig. 2). It was critically important to perform the catalysis without the five *N-*terminal residues; performing the ring closure on the full A-chain sequence (21 mer), unlike other insulin-super family molecules, gave only poor conversion. Hence, ring-closing metathesis (RCM) of the fully protected, truncated resin-tethered peptide **1** (16 mer) was performed in the presence of 20 mol% second-generation Grubb’s catalyst in DCM with 0.4 M w/v LiCl in DMF.

Under these conditions, microwave irradiation of the peptidyl-resin at 100 °C for 2 h resulted in near quantitative conversion to the desired carbocycle **2**. Continued microwave-accelerated SPPS was then performed and the remaining five residues (GIVEQ) were appended to the *N*-terminus to deliver the complete dicarba insulin A chain. Mass spectral analysis of the 21-mer gave the required molecular ions for carbocycle **3** and the RP-HPLC trace showed the formation of two geometric isomers (***E*** **-3** and ***Z*** **-3**) in a 3:1 ratio (see Fig. S1c). Following resin cleavage, crude peptide **3** was exposed to an acidic cleavage mixture containing 2,2′-dipyridyl disulfide to facilitate concerted *tert*-butyl-deprotection and pyridinyl-reprotection of residue CysA7. Each of the resultant isomeric dicarba insulin peptides **4** were then purified before being subjected to regioselective chain coupling. Construction of the complementary insulin B chain **5** was achieved through microwave-accelerated SPPS, in combination with HBTU/HOBt–DIPEA activation and Fmoc-protected amino acids, on preloaded Fmoc-Thr(^*t*^Bu)-PEG-PS resin. During chain elongation, orthogonally protected Cys(Trt) and Cys(Acm) residues were strategically incorporated into the primary sequence in positions B7 and B19 respectively. Preparative RP-HPLC gave the required insulin B chain **5** in 30% yield and 90% purity. The monocyclic A-B conjugates were prepared by combination of the dicarba insulin A chains (***E*** **-4**, ***Z*** **-4**) with the insulin B chain **5** under basic conditions. In all cases, oxidation was complete within minutes giving the required 51 amino acid peptides (Fig. 2, ***E*** **-6** and ***Z*** **-6**). Mass spectral analysis of the isolated solids supported formation of the covalent A-B dimers. The final disulfide bridge in the c[Δ^4^A6,11]-dicarba human insulins (Fig. 2, ***E*** **-7** and ***Z*** **-7**) was formed on exposure of each of the monocyclic A-B conjugates (***E*** **-6**, ***Z*** **-6**) to iodine under acidic conditions. Removal of the acetamidomethyl (Acm) protecting groups at positions A20 and B19 resulted in spontaneous oxidation of the liberated free thiol groups to give the two target isomeric *trans-* and *cis*-dicarba insulin peptides (Fig. 2, peptides ***E*** **-7** and ***Z*** **-7**, respectively)^17^, which were then purified by RP-HPLC and independently subjected to biological testing and structural analysis.

### Stereochemical assignment of the dicarba bridge

The C^β^ chemical shifts of peptides comprising Δ^4^-diaminosuberic acid (Δ^4^Sub) residues show appreciable differences between the *cis-(Z*) and *trans-(E)* configurations^25^. These features were used to identify the stereochemistry of the insulin A-chain A6-A11 dicarba bridge without the need for structural calculations. Hence, TOCSY and ^13^C-HSQC spectra were acquired on each of the dicarba insulin A-chain peptides (***E-*** **4** and ***Z*** **-4**). Δ^4^Sub H^γ^ resonances for each *E-* and *Z-*dicarba isomer were readily identifiable at ~5.6 ppm, which facilitated the assignment of associated Δ^4^Sub H^β^ resonances as well as Δ^4^Sub C^β^ resonances in the ^13^C HSQC. As with other dicarba peptide sequences, only minor differences in carbon chemical shifts were observed between the two isomers, with the major difference occurring at the C^β^ atoms of the dicarba bridge (Fig. S2)^25^. Consistent with the previously reported model for stereochemical assignment, the upfield C^β^ shifts for the *cis* isomer presented at δ30.9 and δ32.8 and those for the *trans* isomer appeared at δ36.4 and δ36.9. The stereochemistry of the *trans* isomer ***E-*** **7** was also confirmed by X-ray crystallography. Additionally, an independent, stereoselective synthesis of *cis*-[A6–11]-dicarba insulin A chain (***Z-*** **3**) (Fig. SIl) was achieved using a preformed, orthogonally protected *Z-*configured diaminosuberic acid residue (*cis-* **S1**)^25^ and SPPS (Fig. S3) and found to be identical to material obtained *via* RCM (Fig. S1c).

### Receptor binding and activation

**Figure 3:**
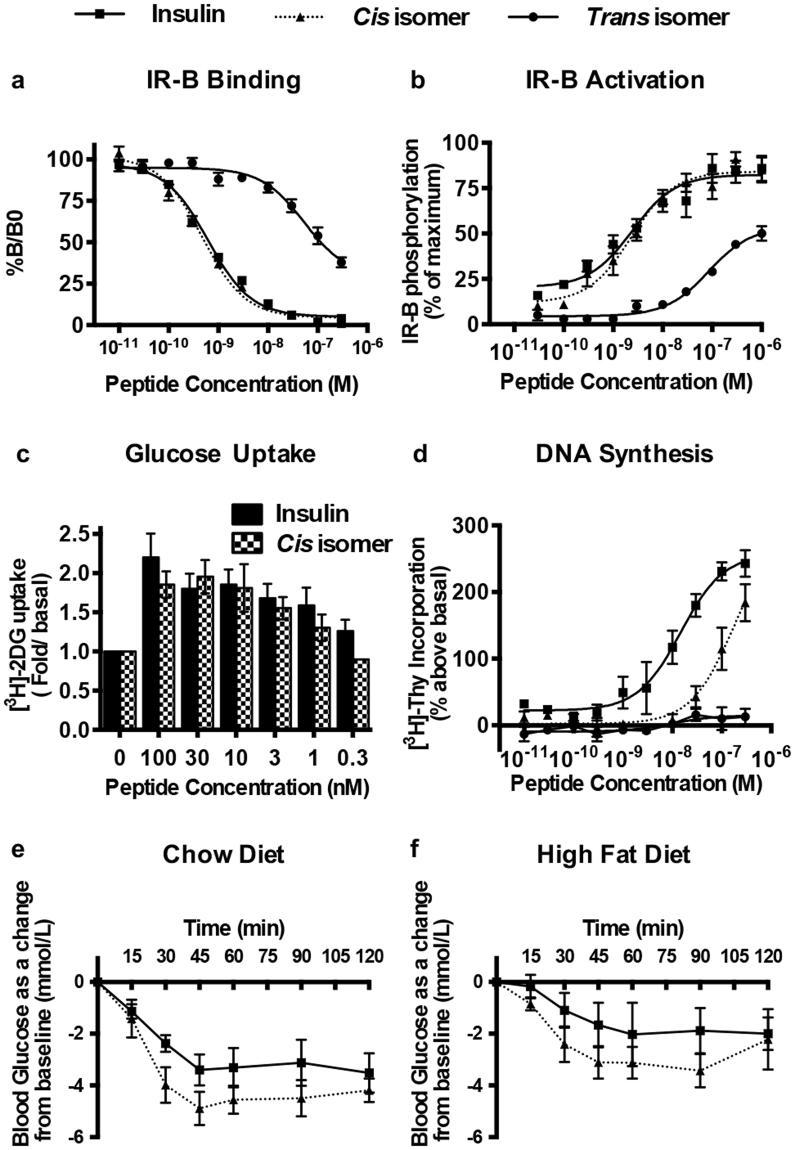
Insulin receptor binding, activation and biological activities of *cis*- and *trans* isomers. (**a**) Competition binding of insulin (squares), *cis*- (triangles) and *trans*- (circles) isomers with europium-labelled insulin. Results are expressed as a percentage of binding in the absence of competing ligand (%B/B0). (**b**) Activation of IR-B by increasing concentrations of dicarba insulins (10 min stimulation) is expressed as receptor phosphorylation as a percentage of the maximal phosphorylation induced by insulin. Insulin *vs cis* isomer (non significant); insulin *vs trans* isomer ****(P ≤ 0.0001) (2-way ANOVA; Dunnett’s multiple comparison) (**c**) Glucose uptake stimulated by increasing concentrations of insulin or *cis* isomer is expressed as fold glucose uptake (pmol/min/mg) above basal. Insulin *vs cis* isomer (ns) (paired *T-test*). (**d**) DNA synthesis in response to increasing concentrations of dicarba insulins is shown as percentage incorporation of ^3^H-thymidine (^3^H-Thy) above basal. All data in (**a**–**d**) are the mean ± S.E.M. n = at least 3 independent experiments. (**e**) Insulin tolerance test in mice fed on a normal diet (chow), or (**f**) on a high fat diet were administered through intraperitoneal injection (ip) with 0.75 IU/kg insulin (squares; solid lines) or *cis* isomer (triangles; dotted lines) under non-fasting conditions and tail vein blood glucose was measured *via* glucose meter at indicated times^27^. n = 5–6 per group. Blood glucose levels are expressed as change over basal levels (mmol/L). Chow diet, insulin *vs* *cis* isomer ** (P ≤ 0.01); high fat diet, insulin *vs* *cis* isomer **(P ≤ 0.01) (paired T-test).

The affinities of the two dicarba insulin isomers for the human insulin receptor isoforms IR-A and IR-B and the human type 1 insulin-like growth factor receptor (IGF-1R) were determined using ligand competition binding assays^26^. The *cis* isomer ***Z-*** **7** was found to bind IR-B and IR-A with similar affinity to native insulin, whereas the *trans* isomer ***E*** **-7** had a ~50-fold lower affinity for IR-B and IR-A than native insulin (Fig. 3a, Table S1 and Fig. S4a). The *cis* isomer ***Z-*** **7** bound IGF-1R with similar affinity to insulin (Table S1 and Fig. S4c), whereas *trans* isomer ***E*** **-7** exhibited negligible affinity to IGF-1R.

In receptor phosphorylation assays, the *cis* isomer was equipotent to insulin in activation of IR-B (Fig. 3b) and IR-A (Fig. S4b), but slightly poorer than insulin in activation of IGF-1R (Fig. S4d), whereas the *trans* isomer was ~1000 fold less potent than insulin in activating IR-B (Fig. 3b). The *trans* isomer had very low binding affinity for the IR-A and IGF-1R and its activity at these receptors was left undetermined.

In summary, these data indicate that (i) only one of the two stereochemical isomers of A6-A11 dicarba insulin (*viz*., the *cis* isomer) is a potent analogue, and (ii) the equipotency of the *cis* analogue to native insulin is maintained despite the increase in rigidity across the A6-A11 connection afforded by the unsaturated dicarba bond.

### Metabolic and mitogenic activity

The *cis* isomer was equipotent to native insulin in promoting glucose uptake by NIH3T3-L1 adipocytes (Fig. 3c), aligning with its equipotency in the above receptor binding and receptor activation assays. However, the *cis* isomer was 5–10 fold less potent than native insulin in promoting DNA synthesis, despite its equal affinity for IR and IGF-1R (Fig. 3d). The *trans* isomer was unable to stimulate DNA synthesis significantly above basal levels, correlating with its poor receptor binding ability.

### Insulin tolerance test

The *cis* isomer lowered blood glucose more effectively than native insulin in an insulin tolerance test in mice (Fig. 3e,f) measured as described^27^. This was also evident in insulin-resistant mice fed on a high-fat diet (Fig. 3f).

### Biophysical characterization

**Figure 4:**
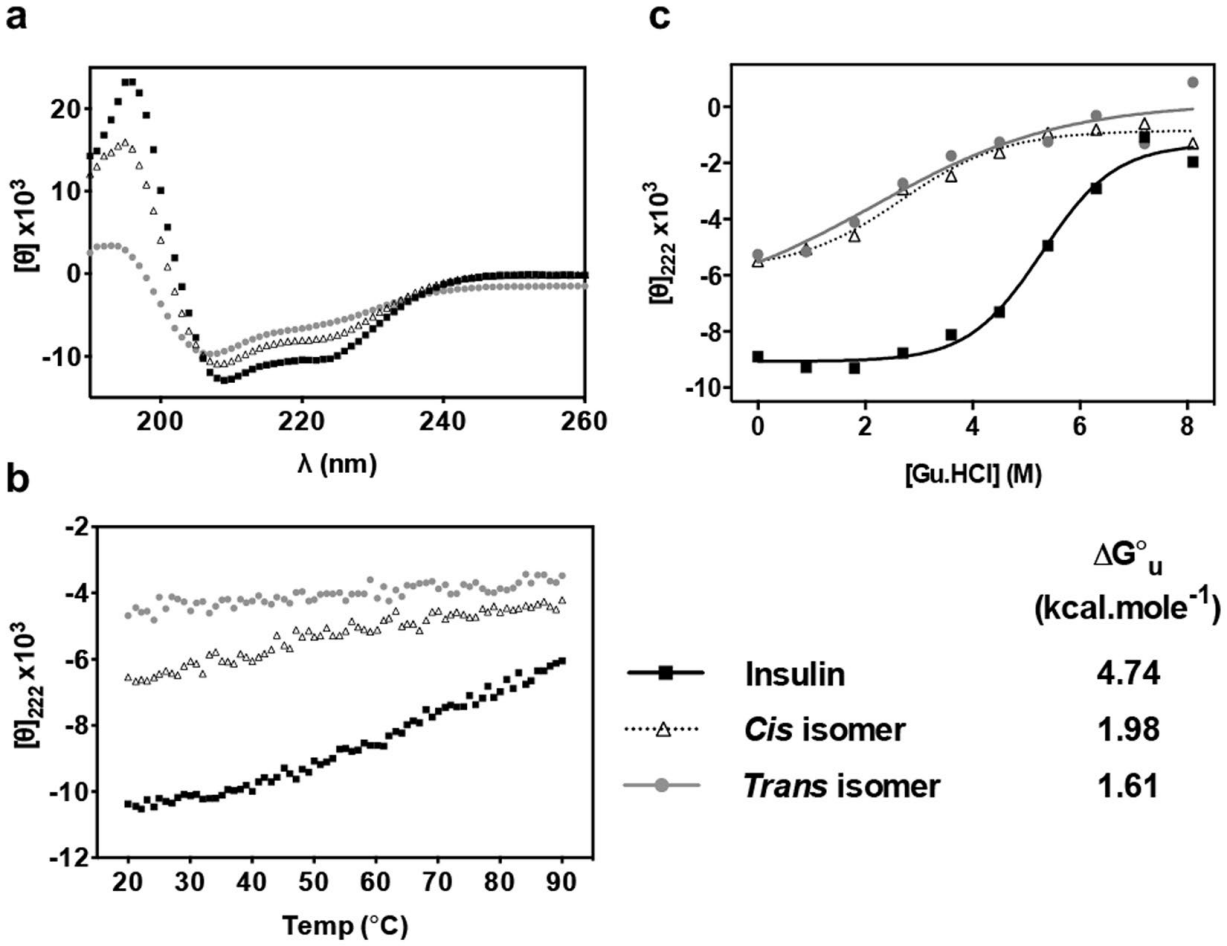
Thermal and chemical stability of *cis-* and *trans* isomers. (**a**) Circular dichroism far-UV spectra reveal lower helical propensities in both the *cis-* and *trans* isomers. *θ* = ellipticity. (**b**) Differences in thermal unfolding are monitored by ellipticity at 222 nm and show both the *cis-* and *trans* isomers are considerably less stable than insulin. (**c**) Unfolding in the presence of guanidine demonstrates that both isomers are considerably destabilized compared to insulin. Δ*G* values derived from guanidine denaturation studies are listed.

The effect of introduction of a dicarba bond at A6-A11 on protein secondary structure and stability was monitored by circular dichroism (CD; Fig. 4). Far UV spectra indicate (Table S2) that both the *cis-* and *trans* isomers have significantly lower helical content (37% and 23%, respectively) than native insulin (48% in our assay, similar to that reported by others^28^). In our experiments, native insulin exhibits a sigmoidal thermal denaturation curve (as monitored by ellipticity at 222 nm) with apparent midpoint *T*_m_ = ~60 °C (Figs 4a and S5b), similar to the previously-reported value^29^. In contrast, both dicarba isomers exhibit only a small decrease in ellipticity with increasing temperature (Figs 4b and S5c). The slope of the *trans* isomer denaturation curve appears slightly lower than that of the *cis* isomer, most likely because the *trans* isomer has a much lower initial helical content. Insulin exhibits a two-state transition upon chemical denaturation (ΔG = 4.74 kcal mol^−1^; Fig. 4c). The dicarba insulin analogues are considerably less stable, with inferred ΔG = 1.98 kcal mol^−1^ for the *cis* isomer and ΔG = 1.6 kcal mol^−1^ for the *trans* isomer (Fig. 4c).

### Crystal structure determination

**Figure 5:**
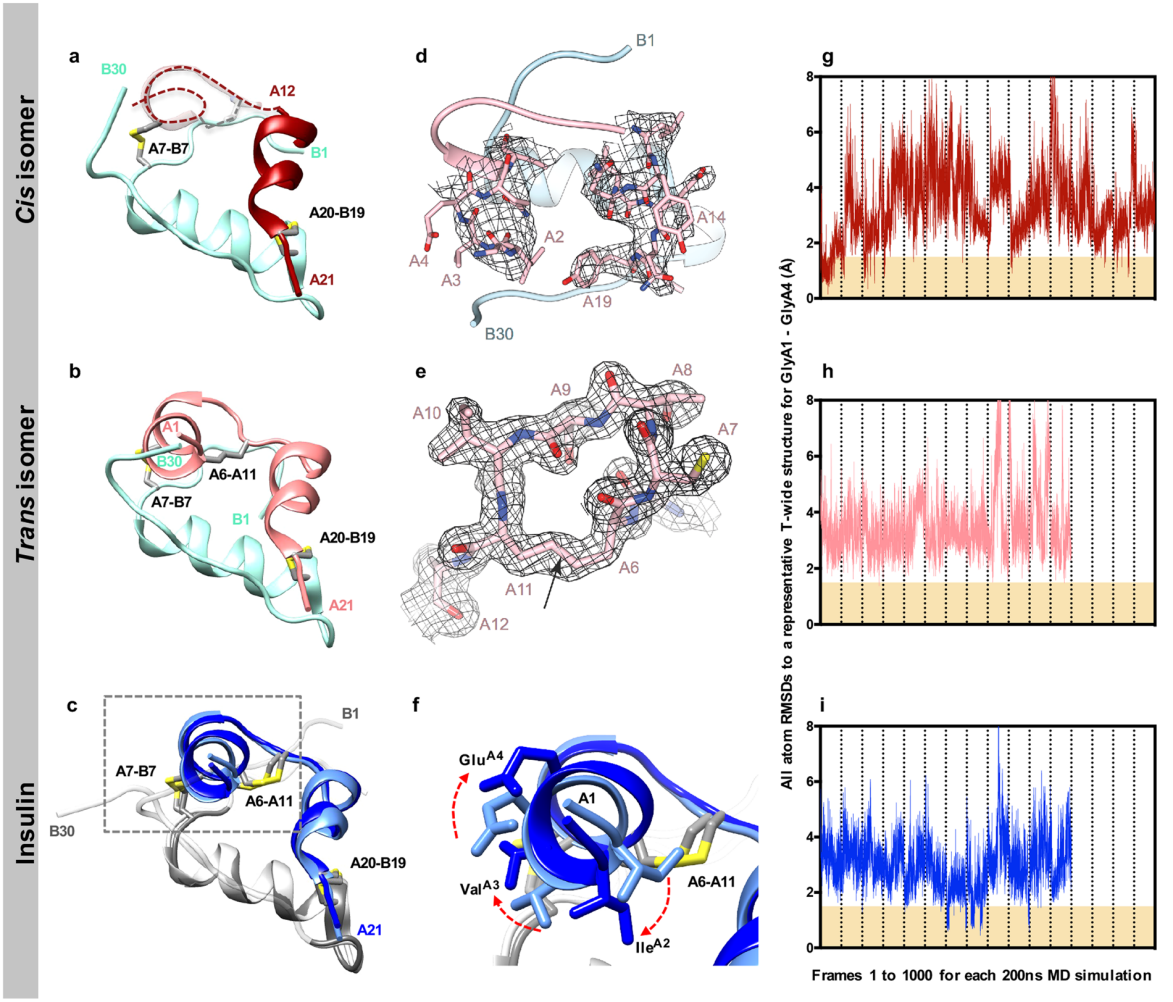
Structural comparison of *cis-* and *trans* isomers with T-state insulin structures. (**a**) The *cis-* and (**b**) *trans* isomers represent T-state insulins with conserved B-chain structures (*cyan*). The *cis* isomer has a poorly defined A1-A8 helix (*dotted line*), whereas the two *trans* isomers in the crystallographic asymmetric unit both display a Class 1 A1-A8 helix. (**c**) The two insulin structures within the asymmetric unit of PDB entry 1MSO aligned across their B chain helices, showing both a Class 1 (*light blue*) and Class 2 (*dark blue*) A1-A8 helix. (**d**) (2*mF*_obs_ − *DF*_calc_) difference electron density associated with the residues within the *N*- and *C*-terminal helices of the A chain of the *cis* isomer; contour level: 1 σ. (**e**) (2*mF*_obs_ − *DF*_calc_) difference electron density in the vicinity of the A6-A11 dicarba bond (*arrowed*) within the *trans* isomer, contour level: 1.3 σ. (**f**) A zoom-in view of the boxed selection in panel (c) highlighting the differences in the A2, A3 and A4 side-chain positions of the two different classes. Analysis of molecular dynamics data of (**g**) *cis* isomer (**h**) *trans* isomer and (**i**) insulin, showing RMSDs of all atoms of residues Gly^A1^ to Glu^A4^ for each simulation frame with respect to a representative Class 2 conformation structure (referred to as T-wide here). Regions corresponding to the Class 2 conformation are shaded *yellow*. Average RMSDs over all simulation frames for insulin, *cis* isomer and *trans* isomer are 3.0 ± 0.9 Å, 3.4 ± 1.3 Å and 3.5 ± 1.0 Å, respectively. Note that each bin between pairs of tick marks on the horizontal axis represents a separate 200 ns simulation.

The X-ray crystal structures of the *cis-* and *trans* isomers were determined using diffraction data to 2.70 Å and 1.55 Å resolution, respectively, with the crystals being grown under similar conditions (see *Methods*). Data processing and refinement statistics are presented in Table 1. Both crystal structures exhibit a T-state-like conformation in that the B chain *N*-terminal segment adopted an extended conformation folded back against the interface between the B chain helix and the polypeptide linker between the two A chain helices (Fig. 5a,b). Both crystal structures also contain dimers formed in a fashion closely similar to that of the classical insulin T-state dimer. In the crystal structure of the *cis* isomer, residues A1 to A10 appear largely disordered, with the A chain *N*-terminal helix being represented by no more than a relatively featureless “blob” of difference electron density (Fig. 5d). The dimensions of this blob loosely approximate that of the polypeptide core of the 8-mer α-helix of the native hormone and likely reflect a crystallographic superposition of the helix in a variety of azimuthal and axial orientations. Various unsuccessful attempts were made to obtain alternative crystals of the *cis* isomer with higher structural definition of the A-chain *N*-terminal helix. Nevertheless, we were able to build a tentative model of the *cis* isomer into the available electron density and to refine this model crystallographically to acceptable *R*_work_/*R*_free_ statistics, though with poor overall stereochemistry (as evidenced by the root-mean-square deviations (RMSDs) of the bond angle and bond lengths from ideality; Table 1). The Ramachandran plot statistics for the two structures are: *trans* isomer: 99% in the favoured region, none in the disallowed region; *cis* isomer: 72% in the favoured region, 11% in the disallowed region (the latter values aligning with the poor stereochemical nature of the model). We note that, in the *trans* isomer structure, the A1-A10 segment is involved in crystal contacts whereas in the *cis* isomer structure the A1-A10 segment is not involved in crystal contacts (or at least not in the putative conformation in which it has been modelled).

**Table 1:** X-ray diffraction data processing and refinement statistics.

	*cis* isomer	*trans* isomer
Data collection^1^		
Space group	I2_1_3	P2_1_3
Cell dimensions *a*, *b*, *c* (Å)	79.78, 79.78, 79.78	77.28, 77.28, 77.28
Resolution (Å)	28.20–2.70 (2.80–2.70)^2^	30–1.55 (1.60–1.55)^2^
*R*_merge_	0.066 (1.653)	0.144 (5.03)
*I*/σ (*I*)	16.19 (1.13)	11.72 (0.38)
*CC*_1/2_	0.999 (0.280)	0.999 (0.107)
Completeness (%)	98.8 (98.9)	0.999 (1.00)
Redundancy	6.0 (6.2)	10.8 (10.4)
Molecules/asymmetric unit	1	2
Refinement		
Resolution (Å)	28.20–2.70	30.0–1.55
No. reflections	2403	22449
*R*_work_/*R*_free_	0.223/0.282^3^	0.195/0.215^3^
No. atoms		
Protein	404	827
Ligand/ion	0	0
Water	0	97
*B*-factors		
Protein (Å^2^)	120.	33.4
Water	n.a.	46.7
R.m.s. deviations		
Bond lengths (Å)	0.011	0.006
Bond angles (°)	1.5	0.8

^1^Diffraction data are from a single crystal in both instances.

^2^Resolution limits were set based on the *CC*_1/2_ correlation statistic being assessed significant at the *P* = 0.001 level of probability.

^3^Free set comprised 5% of the reflections.

### Structural comparison with native insulin

In order to compare the three-dimensional structure of the *trans* isomer determined here with those of native insulin, we began by analysing the conformations of residues A1-A11 in extant insulin crystal structures in the Protein Data Bank (PDB; see Table S3). Inspection of T-state structures of (receptor-free) insulin monomers, including monomers from T_2_, T_6_, T_3_R_3_ and T_3_R^f^_3_ assemblies by alignment of the respective B-chain helices (residues B8-B20) revealed that they could be partitioned into two classes on the basis of the conformation of the A-chain *N*-terminal helix: Class 1 (30 structures), in which residues A1 to A9 exhibit the classical (*i*, *i* + 4) α-helical hydrogen-bonding pattern; and Class 2 (93 structures), wherein residues A1 to A5 form a single α-helical turn and residues A3 to A9 adopt a wider helix conformation, approximating an (*i*, *i* + 5) π-helix (Fig. 5c,f). The distinction between these two classes is most apparent in the hydrogen bonding exhibited by the backbone amide of Thr^A8^: in Class 1 structures the amide forms a canonical α-helical (*i*, *i* + 4) hydrogen bond with the backbone carbonyl oxygen of Glu^A4^; however, in the Class 2 structures, the amide hydrogen bonds to the backbone carbonyl of Val^A3^. In many cases, these two classes occur within the same crystallographic asymmetric unit structure, with 27 out of 30 Class 1 structures being from crystals that also contain within their asymmetric unit an insulin of Class 2 conformation.

Concomitant with these differences in helical conformation are differences in the relative azimuthal positioning of residues A1-A5 about the helix axis (Fig. 5f and Table S4). In addition, the corresponding mean Cα^A6^-to-Cα^A11^ distance of the Class 1 insulins (4.78 ± 0.13 Å) is slightly longer than the mean Cα^A6^-to-Cα^A11^ distance of the Class 2 insulins (4.55 ± 0.37 Å). Concomitantly, the mean Cα^A7^-to-Cα^B7^ distance of the Class 1 insulins (4.62 ± 0.12 Å) is slightly shorter than the mean Cα^A7^-to-Cα^B7^ distance of the Class 2 insulins (4.76 ± 0.11 Å) (Table S5).

**Figure 6:**
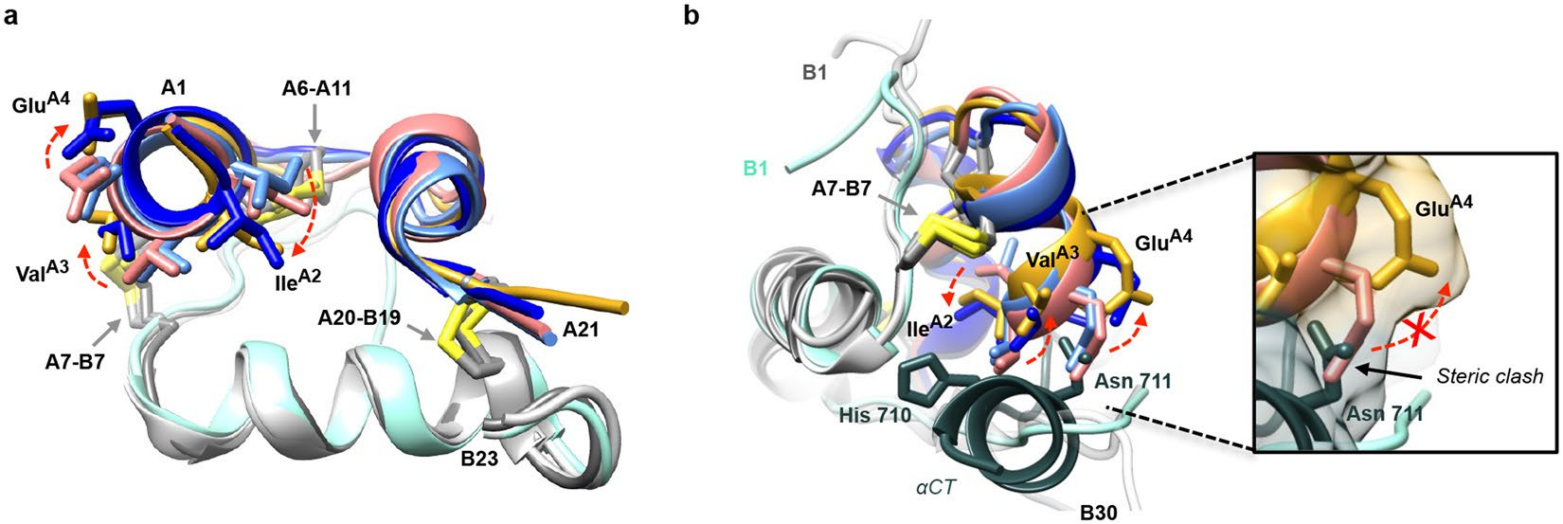
Structural comparison of the *trans* isomer with native insulin in its IR site-1 bound and receptor-free forms. (**a**) Overlay of *trans* isomer (*pink*/cyan) with T6 insulin (PDB entry 1MSO, Class 1 (*light blue/grey*), Class 2 (*dark blue/grey*)) and with IR site-1 bound insulin (PDB entry 4OGA, *gold*/*grey*). Differences in the A2, A3 and A4 side-chain positions arise through rotation of the A1-A8 helix. In the complex this allows accommodation of the IR αCT (not shown). (**b**) In the IR site-1 bound conformation, insulin’s A1-A8 helix adopts a Class 2 conformation and the A2-A4 residues rotate to enable engagement with IR αCT (*dark green*), with insulin A3 and A4 residues flanking Asn711 of IR αCT. This conformation and rotation does not occur in *trans* isomer. Colors are otherwise as in (**a**).

The crystal structure of the *trans* isomer corresponds to a Class 1 insulin conformation. Residues A1-A9 exhibit a classical α-helical geometry, with the azimuthal positioning of residues A1-A5 being closely similar to that of native insulins within Class 1 (Fig. 6a). In addition, the Cα^A6^-to-Cα^A11^ distance (5.17 Å) in the *trans* isomer is more similar to the corresponding average Cα^A6^-to-Cα^A11^ distance of the Class 1 insulins than the average Cα^A6^-to-Cα^A11^ distance of the Class 2 and IR-bound native insulin (PDB entries 4OGA, 3W12, 3W13) (Table S5).

Our analyses are consistent with the report of Kaarlsholm *et al*.^30^ of two forms of insulin that differ at residues A1-A5 through a rotation of 32° about the Cys^A6^ Cα-NH bond in the crystal structure of 2Zn porcine insulin^16^. The Class 2 conformation of the A chain *N*-terminal helix is also observed in insulin analogues synthetically engineered to reposition the B chain *C*-terminal segment away from the hormone core, *e.g*. des[23–30]-insulin (PDB 1DEI)^31^ and NMeAla-B26-DTI insulin analogue (PDB 2WRX)^32^. Our study is, however, the first to recognize the allosteric role played by the A6-A11 disulfide bond in this conformational switch without movement of the B chain *C*-terminal segment.

As far as we can ascertain from the structures of the insulin/IR Site 1-complexes (PDB entries 4OGA, 3W12 and 3W13, at resolution (3.5–3.9 Å)), the A-chain *N*-terminal helix of insulin (and insulin analogues) exhibits a Class 2-like structure when bound to IR (see Fig. 6). This is apparent in the larger-diameter A-chain N-terminal helix and shorter Cα^A6^-to-Cα^A11^ distance than those observed for Class 1 insulins, and in a similar azimuthal positioning of the constituent Cα atoms to those within Class 2 structures. The ability of the insulin A-chain *N*-terminal helix to adopt these two classes of structure indicates both (i) a degree of conformational flexibility in the three covalent bonds that connect that helix to the remainder of the hormone (*viz*., the A6-A11 and A7-B7 disulfide bonds and the downstream peptide bond), and (ii) rotameric plasticity of the side chains of the residues that form the interface between that helix and the remainder of the hormone (Fig. 5c,f).

### Molecular dynamics simulations

Molecular dynamics (MD) simulations were conducted to explore the significance of conformational flexibility in the A6-A11 linkage and the A1-A9 helix — in particular, the distinction between the Class 1 and Class 2 conformations. Three high-resolution X-ray crystal structures were selected, each having a different conformation of the A6-A11 disulfide linkage (PDB entry 1G7B chains E and F, Class 2; PDB entry 1MSO chains A and B, Class 1; and PDB entry 3I3Z chains A and B, Class 2). Each structure was also modified to contain *cis* and *trans* isomers, resulting in nine starting structures. Multiple 200 ns MD simulations were then conducted based on each starting structure. In all simulations, it was evident that the A-chain *N*-terminal helix was highly mobile and, in many cases, interchange between classes was also observed. Indeed, although each starting structure belonged initially to a particular Class, that distinction appeared not to survive the pre-optimization phase, and hence did not bias the simulations.

Root mean square deviations (RMSDs) with respect to a representative Class 2 structure were calculated for all atoms of residues A1 to A4 for each frame of each MD simulation. From the RMSD plots (Fig. 5g–i), it is seen that both insulin and the *cis* isomer can approach the Class 2 conformation (RMSD < ~1.5 Å), albeit rarely (Fig. 5g,i). In contrast, the *trans* isomer almost never adopted a Class 2 conformation, with RMSD values being generally > 2 Å and very rarely < 1.5 Å (two disparate frames out of 12,000 had an RMSD < 1.5 Å) (Fig. 5h). Interestingly, three different hydrogen-bonding partners for the backbone amide of Thr^A8^ were observed in the structures generated in the MD simulations: Val^A3^ (*i*, *i* + 5; π-helix), Glu^A4^ (*i*, *i* + 4; α-helix) and Gln^A5^ (*i*, *i* + 3; 3_10_-helix) (Table S6). For both insulin and the two dicarba isomers, Glu^A4^ and Gln^A5^ were the dominant hydrogen bonding partners. A backbone hydrogen bond between Thr^A8^ and Val^A3^ was seen only in ~4% of simulation frames for insulin and the *cis* isomer and never in any of the *trans* isomer MD runs. Cluster analysis suggests that structures with Thr^A8^-Glu^A4^ and Thr^A8^-Gln^A5^ backbone hydrogen bonds are not distinct conformations and transitions between them are part of a natural “breathing” mode of the helix, possibly explaining why Thr^A8^-Gln^A5^ backbone hydrogen bonds have not been found in extant crystal structures. The average Cα^A6^-to-Cα^A11^ distance for Class 1 insulin and for *cis* isomer structures were effectively the same within the MD trajectories and the X-ray structures (Table S5). Curiously, the average Cα^A6^-to-Cα^A11^ distances for Class 2 structures in the MD simulations (4.05 Å and 3.97 Å for the *cis* isomer and insulin, respectively) were found to be shorter than seen in the crystal structures (4.50 Å and 4.55 Å for the *cis* isomer and insulin, respectively), suggesting that the distance is possibly distorted from its optimal value in the crystalline phase, or that there are differences in the respective stereochemical libraries that underpin MD and crystallographic refinement methodologies.

It is important to note that the variations in the RMSDs through the time course of the simulations (Fig. 5g–i) are larger for the *cis* isomer (σ = 1.3 Å) than for either the *trans* isomer (σ = 1.0 Å) or insulin itself (σ = 0.9 Å). This indicates that the *N*-terminus of the *cis* isomer is more conformationally flexible than that of the other analogues, suggesting an explanation for the disorder of this helix in the X-ray crystallographic maps. Although the *cis* isomer is seen to access the Class 2 conformation in MD simulations and the *trans* isomer is not, their average RMSDs from the Class 2 conformation across all simulation frames are similar (3.4 and 3.5 Å, respectively) and are higher than for insulin (3.0 Å). These increased RMSDs for the dicarba insulin analogues correlate with statistically significant decreases in the helicity of the *N*-terminal A chain residues (as measured by the backbone conformations of the relevant resides) in comparison with that of insulin (see Table S7).

## Discussion

Whereas the role of individual insulin surface residues in receptor binding has been intensively investigated, there has been less examination of the physico-chemical nature of the disulfide bonds, their stereochemistry, and their structural contribution to insulin/IR engagement and resultant biological activities. Here, we have synthesized the two stereoisomers of an A6-A11 dicarba insulin, determined their crystal structures and characterized their biochemical and biophysical nature. Ruthenium-alkylidene catalyzed ring-closing metathesis of insulin A-chain sequences bearing a pair of allylglycine residues provided an expedient route to intra-chain dicarba insulin analogues with different stereochemical configurations.

The dicarba stereoisomers exhibit two intriguing biological characteristics. First, the *cis* isomer is active and the *trans* inactive, as evidenced in *in vitro* assays (receptor binding, receptor activation and glucose uptake by adipocytes). The *cis* isomer also demonstrates promising therapeutic potential, evident from its ability to lower blood glucose more rapidly than native insulin in both normal and insulin-resistant mice. Second, the *cis* isomer is significantly less potent than native insulin in stimulating DNA synthesis, indicating its reduced ability to promote cell growth/replication. The unique biological characteristics of the dicarba stereo-isomers reflect the structural outcomes of the respective rigid configurations of the dicarba linkages. These findings thus presented us with an opportunity to utilize the stereoisomers as tools to scrutinize further the mechanistic role of A6-A11 bond and its influence in mediating the structural transitions of insulin to its active conformation.

To this end we sought to identify structural features that might account for the differences in biological activities of the *cis* and *trans* isomers. Intriguingly, both isomers exhibit reduced helical content as assessed by CD and by MD. In the case of the *cis* isomer, such reduced helical content in solution is consistent with the disorder of the A-chain *N*-terminal helix observed in its crystal structure. In the case of the *trans* isomer, CD measurement indicates a lower helical content than the *cis* isomer. However, both molecules within the crystallographic asymmetric unit of the *trans* isomer show an ordered A chain *N*-terminal helix. Unlike in the structure of the *cis* isomer, the respective A-chain *N*-terminal helices of the two *trans* isomers are involved in crystal contacts, suggesting their crystallographic order may be brought about by these contacts: in solution, the *trans* isomer A chain is likely disordered. In support, our preliminary atomic force microscopy analysis (to be published elsewhere) shows an increased rate of fibrillation of both dicarba isomers compared to that of insulin. A key feature of fibril formation is proposed to be loss of helical structure as the A-chain *N*-terminal helix transitions to a β sheet^33^. Consistent with our CD and MD data, earlier studies show that the A-chain *N*-terminal helix is susceptible to perturbation through removal of the A6-A11 disulfide bond^29,34,35^ or residue substitution elsewhere within the helix^36–39^ and that it can undergo conformational fluctuation^36,40^.

An explanation for the *cis* isomer being active and the *trans* isomer being inactive arises from our analysis of extant T-state insulin structures. The existence of the two Classes (1 and 2) of T-state insulin structures suggests that the hormone is capable of transition between the two forms. As far as can be ascertained at the resolution of the IR-site-1-complexed structures of insulin, the IR-bound conformation of the hormone corresponds to Class 2. The crystal structure of the *trans* isomer indicates that it has a Class 1 conformation, and its inactivity suggests that it is incapable of transitioning to a Class 2 structure. This hypothesis is supported by the MD simulations, which show that, although the *trans* isomer is capable of transiently adopting a range of structures, the Class 2 conformation appears to be precluded. An overlay of *trans* isomer and Class 1 insulin X-ray structures onto those of Class 2 and receptor-bound (PDB entry 4OGA) insulins shows a distinction between the two sets of structures (Fig. 6a), with the *N*-terminal end of the helix axis adopting different rotational configurations with respect to the hormone core (highlighted in *Supplementary Information* Movie 1). The overlay also indicates that there would be a significant steric clash between side chain of Glu^A4^ of the Class 1/*trans* isomer structures and the side chains of Asn^711^ within the receptor αCT helix (Fig. 6b).

Hence, introduction of the *cis* and *trans* dicarba linkages is seen to affect the structural dynamics of the A chain *N*-terminal helix in three ways: (i) increased short-range motion: both *cis* and *trans* linkages disrupt the residue backbone conformations (*i.e*., helicity), as evidenced in both the CD spectroscopy and the MD simulations—this possibly due to the increased rigidity of the dicarba linkages transmitting more vibrational energy from the rest of the molecule into the helix in comparison with the less rigid disulfide bond of insulin; (ii) increased long-range motion in the *cis* isomer, evidenced by the increased range of RMSDs (Fig. 5g) and the corresponding increased standard deviation seen in the MD simulations, as well as in the disorder apparent in the *cis* isomer X-ray structure; and (iii) decreased conformational flexibility in the *trans* isomer: the longer *trans*-dicarba bond (Table S5) changes the conformation of the A6-A11 loop, preventing the formation of a Thr^A8^-Val^A3^ hydrogen bond and hence the adoption of a Class 2 conformation. The inability of the *trans* isomer to adopt a Class 2 conformation (as seen within the MD simulations) then precludes its engagement with IR site 1 and results in its observed biological inactivity.

The activity of the *cis* isomer demonstrates that reduction-oxidation of the native A6-A11 disulfide bond (if it occurs) does not play an obligatory role in IR binding and activation. Rather, we suggest that the A6-A11 disulfide bond is able to modulate the insulin conformation through allostery, key elements of which are revealed by our study. The ability of the A6-A11 cystine bridge to adopt a range of disulfide conformations (Table S3) allows switching of the *N*-terminal region of the insulin A chain between active (Class 2) and inactive (Class 1) conformations. The active conformation requires a short A6-A11 Cα-Cα distance and a concomitant lengthening of the distance between the Cα atoms of A7 and B7 (Table S5). The short A6-A11 Cα-Cα distance of the Class 2 structures appears to result in a “pulling” of the *N*-terminal end of A1-A8 helix away from the volume that would be occupied by IR αCT in a putative receptor complex (a conformation also adopted by mutation of B26 to NMeAla or NMeTyr^32^). In contrast, the longer A6-A11 linkage seen in Class 1 structures results in the base of the A chain *N-*terminal helix being closer to the B chain as a direct consequence of the A7-B7 disulfide bond length (Table S5). Critically, the short A6-A11 Cα-Cα distance seen in the active Class 2 conformations appears to be necessary to allow a precise rotation of the A-chain *N*-terminal helix, which in turn positions the side chains of residues A2 to A4 in an orientation compatible with receptor binding. In insulin, it is the adoption of a particular A6-A11 disulfide conformation that gives the required Cα-Cα distance and hence access to the active conformation. While both *cis* and *trans* A6-A11 dicarba bonds promote flexibility in the A1-A8 helix, it is only the *cis* dicarba linkage that can allow the A6 and A11 Cα atoms to come into the close proximity required to adopt a Class 2 structure. The increased interchain length resulting from the presence of a *trans* carbon-carbon double bond means that the active conformation is not accessible to the *trans* isomer.

## Conclusions and Outlook

Through detailed analysis of the structure and function of the two dicarba insulin isomers generated through novel chemistry, our investigation provides the first description of the key role that the evolutionarily-conserved A6-A11 cystine bridge plays as an allosteric switch of insulin activity. We reveal that both the configuration and flexibility of the A6-A11 disulfide linkage are essential elements in insulin’s adoption of an active conformation. Additionally, we conclude that the A6-A11 dicarba linkage of the *cis* isomer can also adopt a configuration that enables insulin receptor binding. This leads to effective lowering of blood glucose levels in mice and a significantly reduced ability to promote mitogenic signaling, a highly desirable biological property. Thus, the *cis* isomer represents an analogue with promising clinical potential. In conclusion, we have revealed a novel mechanism underlying insulin receptor engagement that dictates downstream biological outcomes. Using these findings as a basis we can now design improved insulin analogues for the treatment of diabetes.

## Methods

### Materials

Actrapid Insulin was purchased from Lyppard Australia Pty Ltd. Hybridoma cells expressing antibodies specific for the IR α subunit (83-7) and the IGF-1R α subunit (24–31) were a gift from Prof. K Siddle^41–43^. [^3^H]-Thymidine was purchased from PerkinElmer Life Sciences. hIR-A and hIR-B overexpressing R^-^fibroblast cells (derived from IGF-1R knockout mouse embryonic fibroblasts, a gift from Prof. R. Baserga (Philadelphia, USA)^44^, were produced according to Denley *et al*.^26^ hIR-A over-expressing L6 myoblasts were provided by Dr. B.F. Hansen (Novo Nordisk A/S, Denmark). P6 cells (BALB/c3T3 cells overexpressing the human IGF-1R) were from Prof. R. Baserga^45^.

### Synthesis of dicarba insulins

An interrupted solid-phase peptide synthesis (SPPS)-catalysis approach^24^ was developed to overcome deleterious aggregation and achieve quantitative ring-closing metathesis of the dicarba insulin A chain. Construction of the complementary insulin B chain was achieved through microwave-accelerated SPPS. The monocyclic A-B conjugates were prepared by combination of the dicarba insulin A chains with the insulin B chain under basic conditions resulting in spontaneous oxidation of the liberated free thiol groups to give the two target isomeric *trans* and *cis* dicarba peptides. The details of this method are provided in the *Supplementary Methods*.

### Receptor competition binding assays and kinase receptor activation assays (KIRA)

Human IR-A, IR-B and IGF-1R binding affinities and receptor phosphorylation were measured as described by Denley *et al*.^26^ Assays were performed in triplicate in at least three independent experiments.

### DNA synthesis assay

DNA synthesis was carried out as described in Gaugin *et al*.^46^. Briefly, L6 rat skeletal myoblasts overexpressing human IR-A, were plated in a 96-well flat bottom plate (1.5 × 10^4^ cells/well) and grown overnight at 37 °C, 5% CO_2_. Cells were starved in serum-free medium for 4 h before treatment with increasing ligand concentrations for 18 h in Dulbecco’s minimal essential medium with 1% bovine serum albumin. The cells were incubated with 0.13 μCi/well [^3^H]-thymidine for 4 h, shaken for 2 h with 50 μL disrupting buffer (40 mM Tris pH 7.5/10 mM EDTA/150 mM NaCl) and then harvested onto glass fibre filters (Millipore^®^) using a MICRO 96^TM^ Skatron harvester (Molecular Devices). The filters were counted in a Wallac MicroBeta counter (PerkinElmer Life Sciences). Assays were performed in triplicate in at least three independent experiments.

### Glucose uptake assay

Briefly, NIH3T3-L1 myoblasts (up to passage 20) grown in DMEM supplemented with 10% newborn calf serum, 2 mM L-glutamine, 100 U/l penicillin, 100 μg/L streptomycin at 37 °C were seeded into 24-well plates at 5 × 10^3^ cells/well and grown for 8 days to confluence and were then differentiated into adipocytes as described^47^. Glucose uptake in response to insulin and the *cis* isomer was measured essentially as described^48^. Briefly, 3T3-L1 adipocytes were serum starved in serum free DMEM/1% BSA for 4 h, washed twice with Krebs-Ringer phosphate buffer (KRP, 12.5 mM HEPES, 120 mM NaCl, 6 mM KCl, 1.2 mM MgSO_4_, 1 mM CaCl_2_, 0.4 mM Na_2_HPO_4_, 0.6 mM Na_2_HPO_4_ (pH 7.4)) containing 1%BSA and incubated for 15 min at 37 °C. Insulin or the *cis* isomer was added at decreasing concentrations (100-0.3 nM) for 30 min at 37 °C. For the final 10 min, 2-deoxyglucose (DOG) uptake was initiated by the addition of 50 μM cold deoxyglucose and 1 μCi ^3^H-deoxyglucose per well. The assay was terminated by rapidly washing the cells three times with ice-cold KRP buffer. Cells were solubilized in 0.5 M NaOH/0.1% SDS and ^3^H content was determined by scintillation counting. Nonspecific 2-DOG uptake was determined in the presence of 50 μM cytochalasin B.

### Insulin tolerance test

Eight-week-old C57BL6 male mice were fed either a standard rodent chow diet containing (wt/wt) 77% carbohydrate, 20% protein, and 3% fat from Ridley AgriProducts (Pakenham, Victoria, Australia) or a high fat diet (HFD) containing (wt/wt) 57% carbohydrate, 19% protein and 15% fat from Specialty Feeds (SF08-044, Glen Forrest, Western Australia, Australia) for 12 weeks. Mice (5 or 6 mice per group) were injected ip with 0.75I U/kg insulin or *cis* isomer under non-fasting conditions and tail vein blood glucose was measured via glucometer at indicated times^27^. Experimental procedures were carried out in accordance to the protocols approved by the Austin Health Animal Ethics Committee (AEC 2011/04396).

### Circular Dichroism (CD)

CD was carried out as previously described^29,49^. Briefly, CD spectra were recorded on a Jasco J-815 CD spectrometer; spectra were from 260 to 190 nm with a 1.0 nm step size using a 1.0 s response time and 1.0 nm bandwidth in a quartz cuvette with a 0.1 cm path length. Insulin and dicarba insulin analogues were diluted in 10 mM phosphate buffer (pH 7.4) to a concentration of 0.22 mg/mL (38 μM). Spectra were background-corrected by subtraction of the spectrum of buffer alone. Temperature denaturation was achieved by automated thermal control increasing by 2°/min at 1° intervals. Samples were diluted to 10 μM for equilibrium denaturation studies in guanidine hydrochloride (1.0–8.0 M).

### Bioinformatics

A dataset of insulin structures was constructed from all medium- and high-resolution X-ray crystal structures (better than 2.8 Å) in PDB Archive Version 4.0 (Jul 2011). 275 unique insulin structures were found in 105 PDB files; these included human, bovine and porcine insulins and synthetic mutants. 152 structures were found to exhibit the R (or R_f_) state (data not shown) and 123 the T state. (Note: structures with A6-A11 Sγ-Sγ bond lengths outside the range of 1.95 to 2.05 Å (indicative of poor modelling of the disulfide), as well as structures involving insulin in complex with the insulin degrading enzyme, were not included in the dataset.) Analyses were performed only on T-state insulins (*i.e*., T-state insulins from monomeric, T_2_, T_6_, T_3_R_3_ or T_3_R^f^_3_ structures). Of the T-state structures, 30 exhibited a Class 1 conformation and 93 a Class 2 conformation. Custom programs were used to extract the Cα^A6^-to-Cα^A11^ and Cα^A7^-to-Cα^B7^ distances and A6-A11 disulfide conformations, as well as the hydrogen bonding pairs, from each structure (see *Supplementary Information*). The approach used to determine the relative azimuthal positioning of residues within the segment A1-A10 of each insulin is also described in the *Supplementary Information*.

### Molecular Dynamics (MD)

Molecular dynamics simulations were performed using the AMBER14 program package^50^. Amber ff14SB force field parameters^51^ were used for all standard amino acid residues. RESP charges^52^ and force-field parameters for the dicarba linkages were determined using the PyRED program^53,54^ (see *Supplementary Information*). Initial atomic coordinates were taken from three high-resolution T-state insulin PDB entries: 1G7B E,F; 1MSO A,B; and 3I3Z A,B. Each of these structures has a different conformation for the A6-A11 disulfide bond. *Cis* and *trans* isomers were created from each entry, giving a total of nine different starting structures. After minimization and equilibration (see *Supplementary Information*), the system was heated to 400 K and the dynamics simulated for 10 ns. Each high-temperature simulation was analysed to identify distinct conformations of the insulin analogue. Four structures (or 8 in the case of the *cis* isomer, see *Supplementary Information*) were chosen from each run to use as starting geometries for subsequent 200 ns room-temperature MD simulations. Full details of simulation protocols are provided in the *Supplementary Information*. Processing of simulations and cluster analysis was performed using CPPTRAJ^55^. The same custom programs as described above under *Bioinformatics* were used to assess the backbone hydrogen-bonding patterns, interatomic distances and A6-A11 disulfide conformations in each MD simulation frame. DSSP^56^ was also used to identify residue backbone conformations.

### Crystallization

*trans* isomer: An initial sparse-matrix sitting-drop vapor-diffusion crystallization screen was conducted at the CSIRO Collaborative Crystallisation Centre (CSIRO C3; Parkville, Australia). Based on hit conditions determined, a *ca* 50 μm crystal was then grown at 20 °C in a 24-well Linbro plate by vapor diffusion from a hanging drop of 1 μL of 1 mg/ml protein dissolved in 10 mM HCl mixed with 2 μL crystallant solution (0.9 M potassium sodium tartrate, 0.1 M Tris HCl (pH 8.5), 0.5% PEG 5000 MME) on a siliconized coverslip (Hampton Research) placed over 0.4 mL crystallant. The crystal was placed in a second hanging drop vapor diffusion condition, a 2 μL drop of 1.0 M potassium sodium tartrate, 0.1 M Tris HCl pH 8.5 and equilibrated against 0.4 mL of 1.5 M potassium sodium tartrate overnight at 20 °C, after which the well solution was exchanged for saturated potassium sodium tartrate and incubated for 24 h. The crystal was cryo-cooled by direct plunging into liquid nitrogen (without addition of a cryo-stabilizing solution). *cis* isomer: Following an initial sparse-matrix screen identical to that described above for the *trans* isomer, a *ca* 25 μm crystal was subsequently grown at CSIRO C3 in a 96-well additive screen using a SWISSCI plate in vapor diffusion format at 20 °C using a sitting drop of 150 nL of 1 mg/ml protein dissolved in 10 mM HCl mixed with 150 nL 0.8 M potassium sodium tartrate, 0.1 M Tris-HCl pH 8.5, 0.5% PEG 5000 MME, 4% acetonitrile. This crystal was briefly dipped in paraffin oil (Hampton Research) and cryo-cooled by direct plunging into liquid nitrogen.

### Diffraction data collection and processing

Diffraction data for both crystals were collected at the MX2 beamline at Australian Synchrotron^57^ at ~100 K and at λ = 0.9537 Å. Diffraction data were processed and merged using XDS^58^; statistics are presented in Table 1.

### Crystallographic structure solution and refinement

Molecular replacement solutions for both isomers were obtained using PHASER^59^, with the starting model in both cases being a porcine insulin monomer obtained from PDB entry 1B2A^60^. Structure refinement was performed with PHENIX^59^ iterated with manual model building within COOT^61^. In the case of the *cis* isomer, the density remained exceptionally poor for the A chain *N*-terminal helix and for residues in vicinity of the dicarba bond. Attempts were made to refine the structure as an ensemble^62^, but without success. Final refinement statistics are presented in Table 1.

### Statistical Analyses

Statistical analysis of receptor binding, receptor activation and DNA synthesis assays were performed using a 2-way ANOVA with a Dunnett’s multiple comparison. Insulin tolerance test and glucose uptake assay data were analysed with a paired *T-test*. Significance was accepted at P < 0.05.

### Data Availability Statement

All data generated or analyzed during this study are included in this published article (and its *Supplementary Information* files). Protein Data Bank (PDB) coordinates for the crystal structure of trans isomer have been deposited with accession code PDB 5T7R. The model coordinates and associated crystallographic structure factors of the cis isomer are included as cis_isomer.cif; the unreliability of the model precludes deposition in the PDB.

## Electronic supplementary material

Supplementary information

Supplementary Movie 1
