# Supplementary material for: Insulin in motion: The A6-A11 disulfide bond allosterically modulates structural transitions required for insulin activity
Source: Sci Rep. 2017 Dec 8;7:17239. doi: 10.1038/s41598-017-16876-3 (PMC5722942; doi:10.1038/s41598-017-16876-3)
Supplement: Supplementary file 1 — Supplementary information [file 41598_2017_16876_MOESM1_ESM.pdf]

Supplementary Information

Insulin in motion: The A6-A11 disulfide bond allosterically modulates structural transitions required for insulin activity

Bianca van Lierop[1,#], Shee Chee Ong[2,#], Alessia Belgi[1], Carlie Delaine[2], Sofianos Andrikopoulos[3], Naomi L. Haworth[1,4,5], John G. Menting[6], Michael C. Lawrence[6,7], Andrea J. Robinson[1,*] and Briony E. Forbes[2,*]

[1]School of Chemistry, Monash University, Clayton, Victoria, 3800, Australia.
[2]College of Medicine & Public Health, Flinders University of South Australia, Bedford Park, 5042, Australia.
[3]University of Melbourne, Department of Medicine, Parkville, Victoria, 3010, Australia.
[4]Research School of Chemistry, Australian National University, Acton, ACT 2601, Australia.
[5]School of Life and Environmental Sciences, Deakin University, Waurn Ponds, Victoria 3216, Australia.
[6]The Walter and Eliza Hall Institute of Medical Research, 1G Royal Parade, Parkville, Victoria 3052, Australia.
[7]Department of Medical Biology, University of Melbourne, Royal Parade, Parkville, Victoria 3050, Australia.
[#]Contributed equally as first authors.
*Contributed equally as corresponding authors and request of materials should be addressed to A.J.R (andrea.robinson@monash.edu) or B.E.F. (briony.forbes@flinders.edu.au)

Supplementary Methods

Peptide materials and reagents

4-Amino-2-pentenoic acid (allylglycine, Agl) was used as supplied by Peptech. Trifluoroacetic acid (TFA) and *N*,*N*-dimethylformamide (DMF) were supplied by Auspep and the latter was stored over 4 Å molecular sieves. Dichloromethane (DCM) and piperidine were supplied by Merck and stored over 4 Å molecular sieves. *N*,*N'*-Diisopropylcarbodiimide (DIC), diisopropylethylamine (DIPEA), 2,2'-dipyridyl disulfide (2,2'-DPDS), *N*-methylmorpholine (NMM), trifluoromethanesulfonic acid (TfOH) and triisopropylsilane (TIPS) were used as supplied by Aldrich. *O*-(7-Azabenzotriazol-1-yl)-*N*,*N*,*N'*,*N'*-tetramethyluronium hexafluorophosphate (HATU), *O*-(benzotriazol-1-yl)-*N*,*N*,*N'*,*N'*-tetramethyluronium hexafluorophosphate (HBTU), *O*-(benzotriazol-1-yl)-tripyrrolidinophosphonium hexafluorophosphate (PyBOP) and *N*-hydroxybenzotriazol (HOBt) were used as supplied by GL Biochem. Fmoc-amino acids were also used as supplied by GL Biochem and reactive sidechains were protected with the Acm, Boc, Pbf, ^{t}Bu, and Trt protection groups. Fmoc-Asp-O^{t}Bu loaded Rink-amide resin was used as supplied by Applied Biosystems.

Catalysts and materials. Tricyclohexylphosphine[1,3-bis(2,4,6-trimethylphenyl)-4,5-dihydro-imidazol-2-ylidene](benzylidene)ruthenium(II) dichloride (2nd generation Grubbs' catalyst) was used as supplied by Aldrich. The solvents used in all metal-catalysed metathesis reactions (DCM and a 0.4 M solution of LiCl in DMF) were degassed prior to use with high purity argon and subjected to a general freeze-pump-thaw procedure. CEM Benchmate™ microwave reactor vessels, equipped with stirrer beads, were employed for ring closing metathesis reactions.

Synthesis of dicarba insulins.

c[Δ^4A6,11]-Dicarba human insulin

1. des$_{A1\text{-}5}$-[A6,11]-Agl-[A7]-Cys(^{t}Bu)-[A20]-Cys(Acm) human insulin A chain **1**. An automated, microwave-accelerated procedure (outlined below and in Robinson *et al.*[1]) was used for the synthesis of peptide **1** (Fig. 2) on Fmoc-Asp-O^{t}Bu loaded Rink-amide resin (164 mg, 100 µmol). Quantities of HATU, DIPEA, piperidine and each Fmoc-amino acid were used as described by the automated protocols of the instrument. After sequence completion, the resin-bound peptide was transferred into a fritted syringe and treated with an acetic anhydride solution (7 mL; DMF : acetic anhydride : NMM; 94 : 5 : 1) for 2 h. The resin was washed with DMF (7 mL; 3 × 1 min), DCM (7 mL; 3 × 1 min) and MeOH (7 mL; 3 × 1 min), then left to dry *in vacuo* for 1 h. Prior to treatment with MeOH, a small aliquot of the resin-bound peptide was removed and subjected to Fmoc-deprotection and to TFA-mediated cleavage (outlined below) for RP-HPLC and mass spectral analysis (Fig. S1a). This supported formation of the desired peptide **1** in 90% purity. Mass spectrum (ESI^+, MeCN : H_2O : TFA): *m/z* 658.3 $[M + 3H]^{3+}$, ⅓($C_{87}H_{137}N_{20}O_{28}S_2$) requires 658.0; 986.6 $[M + 2H]^{2+}$, ½($C_{87}H_{136}N_{20}O_{28}S_2$) requires 986.5; 1315.7 $[2M + 3H]^{3+}$, ⅓($C_{174}H_{271}N_{40}O_{56}S_4$) requires 1314.9. RP-HPLC (Agilent: Vydac C18 analytical column, 15 → 50% buffer B over 35 min): t_R = 18.4 min. Buffer A: 0.1% Aqueous TFA; Buffer B: 0.1% TFA in MeCN.

2. *des$_{A1\text{-}5}$*-*c*[Δ^4A6,11]-Dicarba-[A7]-Cys(^{t}Bu)-[A20]-Cys(Acm) human insulin A chain **2**. Resin-bound peptide **1** was subjected to the general microwave-accelerated RCM procedure[2,3] under the following conditions: Resin-bound **1** (420 mg, 100 µmol), DCM (4 mL), 0.4 M LiCl in DMF (0.2 mL), 2nd generation Grubbs' catalyst (17 mg, 20 µmol), 100 W µwave, 100 °C, 2 h, >95% conversion into **2** (Fig. 2). Post metathesis, a small aliquot of resin-bound peptide was subjected to Fmoc-deprotection and to TFA-mediated cleavage for RP-HPLC and mass spectral analysis (Fig. S1b). RP-HPLC (Agilent: Vydac C18 analytical column, 15 → 50% buffer B over 35 min): t_R = 17.3 min. ESI^+, (MeCN : H_2O : TFA): *m/z* 649.0 $[M + 3H]^{3+}$, ⅓($C_{85}H_{133}N_{20}O_{28}S_2$) requires 648.6; 972.5 $[M + 2H]^{2+}$, ½($C_{85}H_{132}N_{20}O_{28}S_2$) requires 972.4; 1296.6 $[2M + 3H]^{3+}$, ⅓($C_{170}H_{263}N_{40}O_{56}S_4$) requires 1296.3.

3. *c*[Δ^4A6,11]-Dicarba-[A7]-Cys(^{t}Bu)-[A20]-Cys(Acm) human insulin A-chain **3**.

Method A: The automated, microwave-accelerated procedure described above was used to attach the remaining five residues on resin-bound peptide **2** (1.46 g, 200 μmol). Quantities of HATU, DIPEA, piperidine and each Fmoc-amino acid were used as described by the automated protocols of the instrument. After sequence completion, the resin-bound peptide was transferred into a fritted syringe and subjected to Fmoc-deprotection in the presence of 20% v/v piperidine in DMF (7 mL; 1 × 1 min, 2 × 10 min). The resin was washed with DMF (7 mL; 5 × 1 min), DCM (7 mL; 3 × 1 min) and MeOH (7 mL; 3 × 1 min), then left to dry *in vacuo* for 1 h. The resin-bound peptide was subjected to TFA-mediated cleavage. RP-HPLC and mass spectral analysis of the resultant off-white solid supported formation of the desired peptide **3** as *cis-* and *trans* isomers in a 3 : 1 ratio (Fig. S1c and S1d). *Cis* isomer 3: Mass spectrum (ESI$^+$, MeCN : H_2O : TFA): *m/z* 824.5 $[M + 3H]^{3+}$, ⅓($C_{108}H_{171}N_{26}O_{36}S_2$) requires 824.1; 1235.8 $[M + 2H]^{2+}$, ½($C_{108}H_{170}N_{26}O_{36}S_2$) requires 1235.6. RP-HPLC (Agilent: Vydac C18 analytical column, 15 → 50% buffer B over 35 min): t_R = 17.9 min. *Trans*-**3**: Mass spectrum (ESI$^+$, MeCN : H_2O : TFA): *m/z* 824.2 $[M + 3H]^{3+}$, ⅓($C_{108}H_{171}N_{26}O_{36}S_2$) requires 824.1; 1235.5 $[M + 2H]^{2+}$, ½($C_{108}H_{170}N_{26}O_{36}S_2$) requires 1235.6. RP-HPLC (Agilent: Vydac C18 analytical column, 15 → 50% buffer B over 35 min): t_R = 18.9 min.

Method B: Peptide **3** was synthesised with the automated, microwave-accelerated procedure outlined above using Fmoc-Asp-O^{t}Bu loaded Rink-amide resin (164 mg Rink-amide resin, 100 μmol). Quantities of HATU, DIPEA, piperidine and each Fmoc-amino acid were used as described by the automated protocols of the instrument. After construction of the 10-mer (i.e. SLYQLENYCN), the pre-fabricated residue (2*S*,7*S*)-2-((((9*H*-Fluoren-9-yl)methoxy)carbonyl)amino)-8-((4-nitrobenzyl)oxy)-7-((((4-nitrobenzyl)oxy)carbonyl)amino)-8-oxooct-4-enoic acid[4] (*cis*-**S1**, 2 eq, *E/Z ratio* = 1:25) was manually coupled into the sequence. Analysis of the filtrate following coupling showed unreacted acid *cis*-**S1** in unchanged isomeric ratio. After mass spectral analysis of an aliquot of resin-cleaved peptide supported incorporation of the bridging unit, three additional residues were incorporated (*i.e.*, TSI) before the *p*Nb and *p*Nz protecting groups were removed *via* treatment with $SnCl_2$ (6 M) and HCl/DMF (5 mM) in DMF (3 x 2 mL) for 0.5 h. The resin-tethered peptide (12.5 μmol) was then subjected to cyclisation using a solution of PyBOP (65 mg, 125 μmol), HOBt (16.9 mg, 125 μmol) and NMM (27.5 μL, 250 μmol) in DMF (3 mL) and shaken for 6 h at RT. The automated, microwave-accelerated

procedure described above was then used to attach the remaining five residues (i.e. GIVEQ) to afford resin-bound peptide **3**. After sequence completion, the peptide was transferred into a fritted syringe and subjected to Fmoc-deprotection in the presence of 20% v/v piperidine in DMF (7 mL; 1 × 1 min, 2 × 10 min). The resin was washed with DMF (7 mL; 5 × 1 min), DCM (7 mL; 3 × 1 min) and MeOH (7 mL; 3 × 1 min), then left to dry *in vacuo* for 1 h. The resin-bound peptide was subjected to TFA-mediated cleavage and RP-HPLC and mass spectral analysis (Fig. S1l). The *E*/*Z* ratio of the two insulin A-chain isomers, *cis*-**3** and *trans*-**3**, was found to be ~ 1:25 which is consistent with the original isomeric ratio of the incorporated bridging unit *cis*-**S1**. Additionally, the retention times of the two peaks were consistent with previously prepared dicarba insulin A chains prepared *via* Method A described above.

4. *c*[Δ^4A6,11]-Dicarba-[A7]-Cys(Pyr)-[A20]-Cys(Acm) human insulin A chain **4**. The concerted *tert*-butyl-deprotection and pyridinyl-reprotection of Cys_7 was carried out according to a procedure described by Bullesbach *et al.*[5] A solution of ice-cold TFA : TfOH (13.4 mL; 4 : 1) was added to a stirred solution of the cyclic peptide **3** (516.1 mg, 0.2 mmol) and 2,2'-DPDS (227.1 mg, 1.0 mmol) in TFA : anisol (13.4 mL; 9 : 1) at 0 °C. After 1.5 h, the reaction mixture was reduced under a constant stream of air and ice-cold Et_2O (70 mL) was added to induce peptide precipitation. The resultant solid was then collected by centrifugation (3 × 6 min) and analysed by RP-HPLC and mass spectrometry (Fig. S1e and S1f). This supported formation of the *S*-activated peptide **4** as two isomers, *cis*-**4** and *trans*-**4**, in a 3 : 1 ratio. The solid was lyophilised and purified by RP-HPLC (Agilent: Vydac C18 preparative column, 15 → 45% buffer B over 40 min, t_R = 22.8 and 24.4 min). Selected fractions were combined and lyophilised to give two isomers, *cis*-**4** and *trans*-**4**, of the desired peptide as colourless solids (*cis*-**4**: 43.6 mg, 11.3% and *trans*-**4**: 14.7 mg, 11.6%) in >95% purity. *Cis*-**4**: Mass spectrum (ESI$^+$, MeCN : H_2O : TFA): *m/z* 842.1 $[M+3H]^{3+}$, ⅓($C_{109}H_{166}N_{27}O_{36}S_3$) requires 841.7; 1262.7 $[M + 2H]^{2+}$, ½($C_{109}H_{165}N_{27}O_{36}S_3$) requires 1262.1. RP-HPLC (Agilent: Vydac C18 analytical column, 15 → 50% buffer B over 35 min): t_R = 16.7 min. *Trans*-**4**: Mass spectrum (ESI$^+$, MeCN : H_2O : TFA): *m/z* 842.1 $[M+3H]^{3+}$, ⅓($C_{109}H_{166}N_{27}O_{36}S_3$) requires 841.7; 1262.7 $[M + 2H]^{2+}$, ½($C_{109}H_{165}N_{27}O_{36}S_3$) requires 1262.1. RP-HPLC (Agilent: Vydac C18 analytical column, 15 → 50% buffer B over 35 min): t_R = 18.2 min.

5. [B19]-Cys(Acm) human insulin B chain **5**. The synthesis of the peptide was performed according to the automated, microwave-accelerated SPPS procedure described above on Fmoc-Thr(^{t}Bu)-PEG-PS resin (667 mg, 100 μmol). Quantities of DIPEA (N,N-Diisopropylethylamine), HBTU (2-(1*H*-benzotriazol-1-yl)-1,1,3,3-tetramethyluronium hexafluorophosphate), HOBt (1-hydroxy-benzotriazole), piperidine and each Fmoc-amino acid were used as described by the automated protocols of the instrument. A small aliquot of resin-bound peptide was subjected to the cleavage procedure outlined above (Fmoc-deprotection followed by TFA-mediated cleavage) for RP-HPLC and mass spectral analysis (Fig. S1g). This supported formation of the desired peptide **5**. Following global Fmoc-deprotection and TFA-mediated cleavage of the remaining peptide **5** from the resin (1.11 g), the resultant pale yellow solid was purified by RP-HPLC (Agilent: Vydac C18 preparative column, 25 → 45% buffer B over 30 min, t_R = 16.0 min). Selected fractions were combined and lyophilised to give the desired peptide **5** as a colourless solid (106 mg, 30%) in 90% purity. Mass spectrum (ESI$^+$, MeCN : H_2O): *m/z* 876.0 $[M + 4H]^{4+}$, ¼($C_{161}H_{243}N_{41}O_{43}S_2$) requires 875.7; 1167.6 $[M + 3H]^{3+}$, ⅓($C_{161}H_{242}N_{41}O_{43}S_2$) requires 1167.2. RP-HPLC (Agilent: Vydac C18 analytical column, 25 → 45% buffer B over 30 min): t_R = 15.0 min.

6. Monocyclic A-B heterodimer of *c*[Δ^4A6,11]-dicarba human insulin **6**.

cis Isomer: The modified insulin B chain **5** (14.6 mg, 4.17 μmol) in H_2O (12 mL) was added dropwise to a stirred solution of the insulin A chain *cis*-**4** (13.2 mg, 5.23 μmol) in 50 mM NH_4HCO_3 solution (8 mL). Reaction progress was monitored by RP-HPLC and mass spectrometry and after 45 min (Fig. S1h and S1i) the oxidation was terminated by addition of AcOH and lyophilised to give a crude pale yellow solid (51.2 mg) which was used without purification in the next step. *Cis*-**6**: Mass spectrum (ESI$^+$, MeCN : H_2O : TFA): *m/z* 1183.4 $[M + 5H]^{5+}$, ⅕($C_{265}H_{402}N_{67}O_{79}S_4$) requires 1183.0; 1479.0 $[M + 4H]^{4+}$, ¼($C_{265}H_{401}N_{67}O_{79}S_4$) requires 1478.5. RP-HPLC (Vydac C18 analytical column, 0 → 25% buffer B over 5 min then 25 → 50% buffer B over 30 min): t_R = 18.9 min.

trans Isomer: The modified insulin B chain **5** (3.53 mg, 1.00 μmol) in H_2O (6 mL) was added dropwise to a stirred solution of the modified insulin A chain *trans*-**4** (4.94 mg, 1.96 μmol) in 50 mM NH_4HCO_3 (4 mL). Reaction progress was monitored by RP-HPLC and mass spectrometry and after 45 min (Fig. S1), the oxidation was terminated by addition of AcOH and lyophilised to give a crude pale yellow solid (8.3 mg) which was used without

purification in the next step. *trans*-**6**: Mass spectrum (ESI$^+$, MeCN : H_2O : TFA): *m/z* 1183.8 $[M + 5H]^{5+}$, ⅕($C_{265}H_{402}N_{67}O_{79}S_4$) requires 1183.0; 1478.7 $[M + 4H]^{4+}$, ¼($C_{265}H_{401}N_{67}O_{79}S_4$) requires 1478.5. RP-HPLC (Vydac C18 analytical column, 0 → 25% buffer B over 5 min then 25 → 50% buffer B over 30 min): t_R = 18.5 min.

7. *c*[Δ^4A6,11]-Dicarba human insulin **7**.

<u>*cis* Isomer:</u> A 10 mM solution of iodine in glacial acetic acid (22.5 mL) was added to a stirred solution of the crude reaction mixture above (51.2 mg) containing the monocyclic peptide *cis*-**6** (4.17 µmol (max.)) in glacial acetic acid (22.5 mL) and 60 mM HCl (1.5 mL). Reaction progress was monitored by RP-HPLC and after 2.75 h, ice-cold Et_2O (245 mL) was added to induce peptide precipitation. The resultant yellow solid was collected by centrifugation (1 × 10 min) and 20 mM ascorbic acid (2 mL) was then added to quench any excess iodine before analysis *via* RP-HPLC and mass spectrometry (Fig. S1j and S1k). The resultant solid was then purified by RP-HPLC (Agilent: Vydac C18 preparative column, 0 → 30% buffer B over 5 min then 30 → 40% buffer B over 60 min: t_R = 16.4 min). Selected fractions were combined and lyophilised to give *cis*-**7** of the desired *c*[Δ^4A6,11]-dicarba human insulin analogue as a colourless solid (6.1 mg, 26%) in >99% purity. *Cis*-**7**: Mass spectrum (ESI$^+$, MeCN : H_2O : TFA): *m/z* 1154.6 $[M + 5H]^{5+}$, ⅕($C_{259}H_{390}N_{65}O_{77}S_4$) requires 1154.1; 1443.1 $[M + 4H]^{4+}$, ¼($C_{259}H_{389}N_{65}O_{77}S_4$) requires 1442.4. RP-HPLC (Agilent: Vydac C18 analytical column, 0 → 30% buffer B over 5 min then 30 → 40% buffer B over 30 min): t_R = 18.7 min.

<u>*trans* Isomer:</u> A 10 mM solution of iodine in glacial acetic acid (7.5 mL) was added to a stirred solution of the crude reaction mixture above (8.3 mg) containing the monocyclic peptide *trans*-**6** (1.00 µmol (max.)) in glacial acetic acid (7.5 mL) and 60 mM HCl (0.5 mL). Reaction progress was monitored by RP-HPLC and after 2.75 h, ice-cold Et_2O (70 mL) was added to induce peptide precipitation. The resultant solid was collected by centrifugation (1 × 10 min) and 20 mM ascorbic acid (1 mL) was then added to quench any excess iodine before purification by RP-HPLC (Agilent: Vydac C18 preparative column, 0 → 30% buffer B over 5 min then 30 → 40% buffer B over 60 min: t_R = 17.3 min). Selected fractions were combined and lyophilised to give *trans*-**7** of the desired *c*[Δ^4A6,11]-dicarba human insulin analogue as a colourless solid (0.52 mg, 9%) in >99% purity (Fig. S1). *trans*-**7**: Mass spectrum (ESI$^+$, MeCN : H_2O : TFA): *m/z* 1154.8 $[M + 5H]^{5+}$, ⅕($C_{259}H_{390}N_{65}O_{77}S_4$) requires 1154.1; 1443.3 $[M + 4H]^{4+}$, ¼($C_{259}H_{389}N_{65}O_{77}S_4$) requires

1442.4. RP-HPLC (Agilent: Vydac C18 analytical column, 0 → 30% buffer B over 5 min then 30 → 40% buffer B over 30 min): t_R = 14.6 min.

Automated microwave-accelerated peptide synthesis.

Automated microwave-accelerated SPPS was carried out using a CEM Liberty-Discover™ synthesiser. This involved the flow of dissolved reagents from external nitrogen pressurised bottles to a resin-containing microwave reactor vessel fitted with a porous filter. Coupling and deprotection reactions were carried out within this vessel and were aided by microwave energy. Each reagent delivery, wash and evacuation step was carried out according to automated protocols of the instrument controlled by PepDriver software. In a 50 mL centrifuge tube, the resin was swollen with DMF : DCM (10 mL; 1 : 1; 1 × 60 min) and connected to the Liberty™ resin manifold. The Fmoc-amino acids (0.2 M in DMF), activators (0.5 M HBTU/HOBt or HATU in DMF), activator base (2 M DIPEA in NMP) and deprotection agent (20% v/v piperidine in DMF) were measured out and solubilised in an appropriate volume of specified solvent as calculated by the PepDriver software program. The default microwave conditions used in the synthesis of each linear peptide included: Initial deprotection (40 W, 75 ºC, 0.5 min), deprotection (40 W, 75 ºC, 3 min) and coupling (20 W, 75 ºC, 5 min). Cysteine and histidine residues were subjected to modified and lower temperature microwave conditions including: Initial deprotection (40 W, 75 ºC, 0.5 min), deprotection (40 W, 75 ºC, 3 min), pre-activation (0 W, 50 ºC, 2 min) and coupling (25 W, 50 ºC, 4 min). On synthesis completion, the resin-bound peptides were automatically returned to the Liberty™ resin manifold as a suspension in DMF : DCM (1 : 1) and filtered through fritted plastic syringes (5 or 10 mL) prior to acid-mediated cleavage described below. Prior to catalysis, the resin-bound peptides were optionally capped with an acetic anhydride solution (4 mL; 94 : 5 : 1; DMF : acetic anhydride : NMM) for 2 hr, filtered through fritted plastic syringes (5 or 10 mL), washed with DMF (3 x 1 min), DCM (3 x 1 min) then MeOH (3 x 1 min), and dried *in vacuo* for 30 min.

TFA cleavage procedures. A small aliquot of resin-bound peptide (approx. 5 mg) was suspended in cleavage solution (1 mL; TFA : TIPS : water : thioanisol; 95 : 2 : 2 : 1) and shaken gently for 2 h. The mixture was filtered through a fritted syringe and the beads rinsed with TFA (1 × 0.2 mL). The filtrate was concentrated under a constant stream of air

and the resultant oil was induced to precipitate in ice-cold Et_2O (1 mL). Cleaved peptides were collected by centrifugation (3 × 5 min) and dried for analysis by analytical RP-HPLC and mass spectrometry. For full scale resin cleavages, 20 mL of cleavage solution was used and after 4 h, the resin was rinsed with TFA (3 × 2 mL). The filtrate was concentrated under a constant stream of air and the resultant oil was induced to precipitate in ice-cold Et_2O (35 mL). Collection by centrifugation was carried out over 5 × 6 min spin times.

Molecular Dynamics

Molecular dynamics simulations were performed using the AMBER14 software package.[6] Amber ff14SB force-field parameters[7] were used for all standard amino-acid residues. RESP charges[8] and force-field parameters for the dicarba linkages were determined using the PyRED program[9,10] from R.E.D. Server Development,[11] available at http://q4md-forcefieldtools.org/REDServer-Development/, in conjunction with Gaussian 09.[12] In all simulations, the Particle-Mesh Ewald (PME) technique (non-bonded cutoff 9.0 Å) was used to calculate long-range electrostatic interactions. Temperature was maintained with a Langevin thermostat, and bonds involving hydrogen atoms were constrained using the SHAKE algorithm. Initial atomic coordinates were taken from three high-resolution T-state insulin PDB entries: entry 1G7B chains E and F, entry 1MSO chains A and B and entry 3I3Z chains A and B. Each of these structures has a different conformation for the A6-A11 disulfide bond. *cis* and *trans* isomers were created from each entry, giving a total of nine different starting structures. Charges were balanced by the addition of Na^+ ions and the structures solvated with a truncated octahedral box of TIP3P water molecules with a buffer of 9.0 Å surrounding the solute.

The following simulation protocol was used for all starting structures: (i) Atomic coordinates of the two residues of each dicarba linkage were minimised for 500 cycles using the steepest decent algorithm followed by 500 cycles with the conjugate gradient algorithm. All other atomic coordinates were held fixed with a force constant of 500 kcal mol^{-1} $Å^{2}$ (dicarba insulin simulations only). (ii) Solvent coordinates were minimised (500 cycles steepest descent, 500 cycles conjugate gradient). All other atomic coordinates were held fixed with a force constant of 500 kcal mol^{-1} $Å^{2}$. (iii) Coordinates of all atoms were minimised (2000 cycles steepest descent, 3000 cycles conjugate gradient). (iv) The system was then heated to 400 K over 20 ps with constant volume periodic boundary conditions. In this phase the solute coordinates restrained with a force constant of 10 kcal

mol^{-1} Å^2 and the solvent left unrestrained. (v) The entire system was then allowed to equilibrate at 400 K for a further 100 ps with constant pressure periodic boundary conditions. Isotropic position scaling with a relaxation time of 2 ps was used to maintain the pressure at 1 atm. (vi) Finally, the simulation was allowed to run for a further 10 ns at 400 K, again with constant pressure dynamics, with snapshots being recorded every 2 ps.

Each of the high-temperature simulations was analysed to identify distinct conformations of the insulin analogue. Four structures were chosen from each run to use as starting geometries for room-temperature simulations. Note: eight structures were chosen from the *cis* isomer simulation based on PDB entry 1MSO as these had a different orientation of the dicarba bond to those based on PDB entries 1G7B and 3I3Z, giving a total of eight simulations with the *cis* dicarba bond in each orientation.

For each of the resulting 40 structures, the following protocol was used for the production MD runs: (i) The solvent was cooled to 300 K over 10 ps with constant volume periodic boundary conditions and the solute coordinates restrained with a force constant of 10 kcal mol^{-1} Å^2. (ii) The entire system was equilibrated at 300 K for a further 100 ps with constant pressure periodic boundary conditions. Isotropic position scaling with a relaxation time of 2 ps was used to maintain the pressure at 1 atm. (iii) MD runs of 200 ns were carried out with constant pressure dynamics at 300 K. Snapshots were saved every 2 ps.

Processing of simulations and cluster analysis was performed using CPPTRAJ.[13] The same custom programs as described above under *Bioinformatics* were used to assess the backbone hydrogen bonding patterns, interatomic distances and A6-A11 disulfide conformations in each MD simulation frame. DSSP[14] was also used to identify residue backbone conformations.

Bioinformatics and Molecular Dynamics Structure Analysis

A custom program was also used to assess the backbone hydrogen-bonding pattern within each insulin structure, the program being based on the energy of NH-CO interaction as calculated from the electrostatic function within the DSSP algorithm.[14]

Equation 1:

$$E = q_1 q_2 \left[\frac{1}{r_{ON}} + \frac{1}{r_{CH}} - \frac{1}{r_{OH}} - \frac{1}{r_{CN}}\right] \times 1390 \text{ kJ mol}^{-1}$$

Interactions with $E < -2.1$ kJ mol^{-1}, r(H…O) ≤ 2.45 Å, a(N-H…O) ≥ 125° and a(H…O-C) ≥ 90° were identified as hydrogen bonds.

The relative azimuthal positioning of residues within the segment A1-A10 of each insulin was computed as follows. First, the direction vectors $\vec{a}$ and $\vec{b}$ of the helices defined by residues A1-A8 and B9-B19 respectively were computed using the program HELIXANG within the CCP4 suite,[15] as well as the vector $\vec{d}$ defining the line of closest approach between the two helical axes $\vec{a}$ and $\vec{b}$. The azimuthal angle ϕ_n of the n'th residue within the segment A1-A10 was then defined as the angle between the vector $\vec{d}$ and the vector $\vec{n}$, where $\vec{n}$ is defined as the vector defining the line and direction of closest approach of the Cα atom of residue n and the helical axis $\vec{a}$ (see Fig. S7).

Supplementary Table S1. Inhibition of europium-labeled insulin and IGF-I for binding to the IR-B and IGF-1R, respectively, by insulin, dicarba insulin isomers and IGF-I.

	**IR-B**[a]		**IGF-1R**[a]	
	IC_{50} (nM)	IC_{50} relative to insulin	IC_{50} (nM)	IC_{50} relative to insulin
Insulin	0.86 ± 0.10	1.0	>100	1.0
cis isomer	0.75 ± 0.16[c]	0.87[ns]	>100[b]	~1.0
trans isomer	40.4 ± 7.2	47****	-	-
IGF-I	-	-	0.30 ± 0.08	<0.003

[a] n=3 or more; [b] single experiment; [c] n=2; - not performed; [ns] non-statistically significant, $P > 0.05$, **** $P \leq 0.0001$. Errors shown are S.E.M.

Supplementary Table S2. Secondary structure content of insulin and dicarba insulin isomers from CD analysis

	**Helix (%)**	**Sheet (%)**	**Turn (%)**	**Unordered (%)**
Insulin	48	12	14	26
cis isomer	37	16	20	27
trans isomer	23	24	22	31

Secondary structure content was calculated using the CONTINLL algorithm for deconvolution against the protein database reference set SP43. The program is available on the DICROWEB website (http://dichroweb.cryst.bbk.ac.uk/html/home.shtml).

Supplementary Table S3. Bioinformatics study of T state structures within medium- to high-resolution (<2.8 Å) X-ray crystal structures of insulins, including T_2 (TT), T_6 (TT), T_3R_3 (TR) and $T_3R^f_3$ (TR) assemblies and T monomers (T_M)) grouped into two classes based on the conformation of the A chain *N*-terminal helix. Chain identifiers are used to distinguish between different insulin molecules within the same crystallographic asymmetric unit. Structures used as starting points for the MD simulations are highlighted in **bold**. Amino acid sequences are given for each polypeptide chain of each structure; hyphens are used to represent amino acids that are missing or not resolved in the X-ray crystallographic maps as well as non-standard residues. For the critical A6-A11 disulfide bond, both the conformation and the distance between the cysteine Cα atoms (r(C_α-C_α) /Å) are reported; mean Cα-Cα distances for each structural class are in **Supplementary Table S5**. The hydrogen-bonding pattern in the *N*-terminal region of the A chain is reported in terms of the hydrogen-bonding partners of the carbonyl oxygens of residues Gly^{A1} to Gln^{A5}. The relative backbone azimuthal angle ϕ of each residue in the A chain *N*-terminal helix is also given; the method of calculation of ϕ is as described in the **Supplementary Methods** and illustrated in **Supplementary Figure S7**. The mean ϕ values for each class are in **Supplementary Table S4**. Diagrams of the disulfide bond conformations are provided in **Supplementary Figure S6**.

PDB Code	Chain IDs in PDB		Context	A Chain Sequence	B Chain Sequence	A6-A11		H bond partner of CO					Azimuthal angles ϕ/°										Class
						r(C_α-C_α) /Å	Conformation	A1	A2	A3	A4	A5	A1	A2	A3	A4	A5	A6	A7	A8	A9	A10	
1IZB	A	B	TR	GIVEQCCTSICSLYQLENYCN	FVNQHLCGSHLVQALYLVCGERGFFYTPKT	4.6	right-handed G'GT	5	6		8	9	17.4	262.7	170.1	95.4	340.4	250.2	170.5	50.2	333.2	287.7	1
1M5A	A	B	TT	GIVEQCCTSICSLYQLENYCN	FVNQHLCGSHLVEALYLVCGERGFFYTPKA	4.6	right-handed G'GT	5	6		8	9	25.6	265.1	169.0	91.6	338.6	250.6	169.4	49.9	329.7	286.2	1
**1MSO**	A	B	TT	GIVEQCCTSICSLYQLENYCN	FVNQHLCGSHLVEALYLVCGERGFFYTPKT	4.9	right-handed G'GT	5	6		7,8	9	24.8	266.8	169.3	91.8	337.8	247.1	167.9	47.9	328.7	284.2	1
1OS3	A	B	TT	GIVEQCCTSICSLYQLENYCN	FVNQHLCGSHLVEALYLVCGERGFFYTPK-	4.8	right-handed G'GT	5	6		7,8	9	43.0	289.3	188.8	107.4	347.2	250.9	174.5	55.3	339.9	296.0	1
1OS4	A	B	TT	GIVEQCCTSICSLYQLENYCN	FVNQHLCGSHLVEALYLVCGERGFFYTPK-	4.6	right-handed G'GT	5	6	7	8	9	58.1	288.3	193.1	109.3	348.1	254.2	178.8	58.2	342.8	296.4	1
1OS4	E	F	TT	GIVEQCCTSICSLYQLENYCN	FVNQHLCGSHLVEALYLVCGERGFFYTP--	4.6	right-handed G'GT	5	6		8	9	46.4	286.0	186.8	101.2	345.0	251.2	172.5	53.4	336.0	291.6	1
1OS4	I	J	TT	GIVEQCCTSICSLYQLENYCN	FVNQHLCGSHLVEALYLVCGERGFFYTP--	4.7	right-handed G'GT	5	6		8	9	45.9	292.7	189.4	101.8	344.8	249.9	174.1	56.7	336.5	291.7	1
2A3G	A	B	TT	GIVEQCCASVCSLYQLENYCN	FVNQHLCGSHLVEALYLVCGERGFFYTPKA	4.7	right-handed G'GT	5	6		7,8	9	28.1	265.5	168.1	89.2	339.7	249.4	172.5	51.5	333.6	287.9	1
2R35	C	D	TR	GIVEQCCTSICSLYQLENYCN	FVNQHLCGSHLVEALYLVCGERGFFYTPKT	4.4	right-handed hook			7			18.4	203.1	140.4	50.6	316.6	246.4	165.1	49.7	324.7	283.8	1
2VK0	C	D	TT	GIVEQCCTSICSLYQLENYCN	--NQHLCGSHLVEALYLVCGERGFFYTPK-	4.7	left-handed GGT	5	6		8	9	29.2	266.0	173.1	97.0	338.9	247.7	169.7	44.7	330.1	294.5	1
2ZP6	A	B	TT	GIVEQCCASVCSLYQLENYCN	FVNQHLCGSHLVEALYLVCGERGFFYTPKA	4.8	right-handed G'GT		6		8	9	19.8	259.3	163.6	78.1	338.1	244.8	170.8	48.2	333.2	286.3	1
3BRR	A	B	TT	GIVEQCCTSICSLYQLENYCN	FVNQHLCGSHLVEALYLVCGERGFFYTPKA	4.8	right-handed G'GT	5	6		8	9	23.7	263.8	169.6	92.7	338.3	249.2	171.6	53.1	334.3	289.8	1
3BXQ	A	B	TT	GIVEQCCTSICSLYQLENYCN	FVNQRLCGSHLVEALYLVCGERGFFYTPKT	4.9	right-handed G'GT	5	6		7,8	9	27.5	267.8	170.7	92.3	339.3	249.1	172.5	51.8	331.4	286.2	1
3E7Y	C	D	TT	GIVEQCCTSICSLYQLENYCN	FVNQHLCGSHLVEALYLVCGERGFFYTPK-	4.8	right-handed G'GT	4,5	6		7,8	9	31.8	279.5	173.8	95.3	340.0	249.8	170.1	50.7	333.1	288.6	1
3E7Z	C	D	TT	GIVEQCCTSICSLYQLENYCN	FVNQHLCGSHLVEALYLVCGERGFFYTPK-	4.8	right-handed G'GT	5	6		8	9	28.5	259.3	169.6	89.5	335.7	249.6	171.0	50.6	332.8	287.8	1
3EXX	A	B	TT	GIVEQCCTSICSLYQLENYCN	FVNQHLCGSHLVEALYLVCGERGFFYTPKT	4.9	right-handed G'GT	5	6		8	9	23.9	266.6	265.3	169.9	92.8	337.4	248.4	169.1	48.8	329.9	1
3FHP	A	B	TT	GIVEQCCTSICSLYQLENYCN	FVNQHLCGSHLVEALYLVCGERGFFYTPKA	4.8	right-handed G'GT	5	6		8	9	31.9	269.1	171.4	92.5	338.5	248.0	169.8	49.0	328.8	284.6	1
3FQ9	A	B	TT	-IVEQCC-SICSLYQLENYCN	FVNQHLCGSHLVEALYLVCGERGFFYTPKT	4.8	right-handed G'GT		6				28.4	268.6	172.3	94.0	339.3	249.8	173.8	53.9	334.8	290.3	1
3ILG	A	B	TT	GIVEQCCTSICSLYQLENYCN	FVNQHLCGSHLVEALYLVCGERGFFYTPKT	4.8	right-handed G'GT	5	6		8	9	23.7	264.4	169.4	91.2	338.8	248.9	168.7	49.7	334.6	287.7	1
3INC	A	B	TT	GIVEQCCTSICSLYQLENYCN	FVNQHLCGSHLVEALYLVCGERGFFYTPKT	4.8	right-handed G'GT	5	6		8	9	26.7	264.8	170.7	93.1	336.7	248.7	168.5	49.9	333.3	288.2	1
3INS	A	B	TT	GIVEQCCTSICSLYQLENYCN	FVNQHLCGSHLVEALYLVCGERGFFYTPKA	4.8	right-handed G'GT	5	6		8	9	23.1	266.4	171.0	93.8	340.6	249.6	171.5	50.3	332.3	288.1	1
3IR0	A	B	TT	GIVEQCCTSICSLYQLENYCN	FVNQHLCGSHLVEALYLVCGERGFFYTPKT	5.0	right-handed G'GT	5	6		7,8	9	14.9	260.1	168.2	88.1	346.9	252.0	171.7	45.7	326.8	284.5	1
3IR0	E	F	TT	-IVEQCCTSICSLYQLENYCN	FVNQHLCGSHLVEALYLVCGERGFFYTPKT	4.8	right-handed G'GT		6		8												1

3IR0	I	J	TT	GIVEQCCTSICSLYQLENYCN	FVNQHLCGSHLVEALYLVCGERGFFYTPKT	4.9	right-handed G'GT	5	6		7,8	9	12.0	256.2	160.6	82.7	336.0	240.5	164.6	40.4	320.3	275.8	1
3IR0	M	N	TT	GIVEQCCTSICSLYQLENYCN	FVNQHLCGSHLVEALYLVCGERGFFYTPKT	5.0	right-handed G'GT	5	6		7,8	9	20.0	264.9	169.2	91.0	344.8	248.5	172.1	47.4	325.8	282.2	1
3IR0	R	S	TT	GIVEQCCTSICSLYQLENYCN	FVNQHLCGSHLVEALYLVCGERGFFYTPKT	4.7	right-handed G'GT	5	6		8	9	31.9	246.0	160.8	79.7	338.5	252.6	179.4	53.0	331.5	291.3	1
3IR0	V	W	TT	GIVEQCCTSICSLYQLENYCN	-VNQHLCGSHLVEALYLVCGERGFFYTPKT	4.9	right-handed G'GT	5	6		7,8	9	18.3	260.2	167.1	88.4	344.1	250.6	177.4	51.7	332.1	286.3	1
3Q6E	A	B	TT	GIVEQCCTSICSLYQLENYCN	FVNQHLCGSHLVEALYLVCGERGFFYTPK-	4.9	left-handed GGT	5	6		7,8	9	17.1	262.0	169.8	91.9	345.8	257.3	172.0	52.8	331.6	288.8	1
3RTO	A	B	TT	GIVEQCCTSICSLYQLENYCN	FVNQHLCGSHLVEALYLVCGERGFFYTPKA	4.8	right-handed G'GT	5	6		8	9	22.2	259.7	257.9	169.3	88.2	338.4	248.5	171.8	53.2	333.5	1
4INS	A	B	TT	GIVEQCCTSICSLYQLENYCN	FVNQHLCGSHLVEALYLVCGERGFFYTPKA	4.8	right-handed G'GT	5	6		8	9	22.4	264.2	170.2	93.8	341.6	249.4	171.9	48.7	332.0	287.5	1
1B17	A	B	T_M	GIVEQCCTSICSLYQLENYCN	FVNQHLCGSHLVEALYLVCGERGFFYTPKA	3.8	right-handed hook	5	6	7,8			52.4	213.6	139.7	44.1	314.1	247.3	169.2	65.9	340.3	289.7	2
1B18	A	B	T_M	GIVEQCCTSICSLYQLENYCN	FVNQHLCGSHLVEALYLVCGERGFFYTPKA	3.9	right-handed hook	5	6	7,8			54.0	212.7	140.7	44.0	313.9	247.5	169.7	66.4	340.4	289.8	2
1B19	A	B	T_M	GIVEQCCTSICSLYQLENYCN	FVNQHLCGSHLVEALYLVCGERGFFYTPKA	3.9	right-handed hook	5	6	7,8			53.1	213.2	141.5	45.2	313.9	248.2	169.9	66.1	341.1	290.7	2
1B2A	A	B	T_M	GIVEQCCTSICSLYQLENYCN	FVNQHLCGSHLVEALYLVCGERGFFYTPKA	3.8	right-handed hook	5	6	7,8			52.3	212.6	141.0	44.9	314.3	248.0	170.2	66.5	341.0	290.5	2
1B2B	A	B	T_M	GIVEQCCTSICSLYQLENYCN	FVNQHLCGSHLVEALYLVCGERGFFYTPKA	3.9	right-handed hook	5	6	7,8			52.9	213.1	141.8	45.5	313.9	248.1	170.2	66.3	341.0	290.5	2
1B2C	A	B	T_M	GIVEQCCTSICSLYQLENYCN	FVNQHLCGSHLVEALYLVCGERGFFYTPKA	3.9	right-handed hook	5	6	7,8			55.0	213.1	141.5	45.3	314.1	248.5	170.0	66.6	341.8	291.1	2
1B2D	A	B	T_M	GIVEQCCTSICSLYQLENYCN	FVNQHLCGSHLVEALYLVCGERGFFYTPKA	3.9	right-handed hook	5	6	7,8			52.6	213.0	141.8	45.3	314.6	248.1	169.7	65.8	340.9	290.2	2
1B2E	A	B	T_M	GIVEQCCTSICSLYQLENYCN	FVNQHLCGSHLVEALYLVCGERGFFYTPKA	3.9	right-handed hook	5	6	7,8			54.0	212.6	141.6	46.4	315.2	248.9	171.4	67.5	341.7	291.3	2
1B2F	A	B	T_M	GIVEQCCTSICSLYQLENYCN	FVNQHLCGSHLVEALYLVCGERGFFYTPKA	3.9	right-handed hook	5	6	7,8			53.8	212.0	141.7	46.1	315.3	249.1	170.7	67.3	339.9	290.0	2
1B2G	A	B	T_M	GIVEQCCTSICSLYQLENYCN	FVNQHLCGSHLVEALYLVCGERGFFYTPKA	3.9	right-handed hook	5	6	7,8			57.3	214.6	141.6	47.4	316.3	248.6	172.2	67.8	340.6	290.7	2
1B9E	A	B	TT	GIVEQCCTSICSLYQLENYCN	FVNQHLCGEHLVEALYLVCGERGFFYTPKT	4.8	right-handed G'GT	5	6	7,8	9		38.6	213.5	139.3	47.3	316.7	246.4	168.8	61.3	337.5	288.0	2
1B9E	C	D	TT	GIVEQCCTSICSLYQLENYCN	FVNQHLCGEHLVEALYLVCGERGFFYTPKT	4.8	right-handed G'GT	5	6	7,8			36.7	212.3	139.7	49.1	315.1	246.3	168.8	60.6	337.2	287.4	2
1BEN	A	B	TR	GIVEQCCTSICSLYQLENYCN	FVNQHLCGSHLVEALYLVCGERGFFYTPKT	4.7	left-handed GGT	5	6	7,8	9		39.9	210.1	137.7	45.4	316.5	248.5	171.4	63.9	337.8	286.0	2
1BPH	A	B	T_M	GIVEQCCASVCSLYQLENYCN	FVNQHLCGSHLVEALYLVCGERGFFYTPKA	3.9	right-handed hook	5	6	7,8			68.0	236.1	156.7	64.9	334.6	265.8	189.3	83.0	355.0	305.0	2
1DEI	A	B	TT	GIVEQCCTSICSLYQLENYCN	FVNQHLCGSHLVEALYLVCGERG-------	4.7	right-handed G'GT	5	6	7,8	9		36.7	217.7	139.5	43.5	315.0	248.5	168.9	65.6	340.7	287.2	2
1DEI	C	D	TT	GIVEQCCTSICSLYQLENYCN	FVNQHLCGSHLVEALYLVCGERG-------	4.7	right-handed G'GT	5	6	7,8	9		26.8	215.8	142.1	48.1	313.7	246.5	167.6	60.7	339.2	289.6	2
1DPH	A	B	T_M	GIVEQCCASVCSLYQLENYCN	FVNQHLCGSHLVEALYLVCGERGFFYTPKA	4.0	right-handed hook	5	6	7,8			65.1	242.5	171.4	78.7	346.2	277.8	201.1	92.3	6.7	317.9	2
**1G7A**	A	B	TR	GIVEQCCTSICSLYQLENYCN	FVNQHLCGSHLVEALYLVCGERGFFYTPKT	4.6	left-handed GGT	5	6	7,8			52.3	205.0	133.0	41.7	313.3	246.9	170.7	64.0	334.2	283.6	2
1G7A	E	F	TR	GIVEQCCTSICSLYQLENYCN	FVNQHLCGSHLVEALYLVCGERGFFYTPKT	4.6	left-handed GGT	5	6	7,8	9		45.3	210.7	137.6	45.9	317.4	316.2	248.9	171.1	64.1	338.5	2
1G7B	A	B	TR	GIVEQCCTSICSLYQLENYCN	FVNQHLCGSHLVEALYLVCGERGFFYTPKT	4.5	left-handed GGT	5	6	7,8			53.1	205.9	133.7	42.5	313.6	247.8	170.9	65.1	334.3	284.6	2
1G7B	E	F	TR	GIVEQCCTSICSLYQLENYCN	FVNQHLCGSHLVEALYLVCGERGFFYTPKT	4.6	left-handed GGT	5	6	7,8			44.1	211.8	137.7	45.5	316.4	248.6	171.0	63.9	338.4	338.3	2
1GUJ	A	B	TT	GIVEQCCTSICSLYQLENYCN	FVNQHLCGSHLVEALYLVCGERGFFYTPKT	4.6	right-handed G'GT	5	6	7,8	9		29.0	212.5	138.5	48.0	316.4	247.5	170.1	62.7	339.3	288.6	2
1GUJ	C	D	TT	GIVEQCCTSICSLYQLENYCN	FVNQHLCGSHLVEALYLVCGERGFFYTPKT	4.7	right-handed G'GT	5	6	7,8	9		27.6	210.3	135.9	42.2	311.5	244.9	165.2	60.2	336.9	285.0	2
1IZA	C	D	TR	GIVEQCCTSICSLYQLENYCN	FVNQHLCGSHLVQALYLVCGERGFFYTPKT	5.0	right-handed G'GT	5	6	8			29.5	204.7	133.6	42.4	313.9	252.4	170.7	66.9	340.2	287.0	2
1IZB	C	D	TR	GIVEQCCTSICSLYQLENYCN	FVNQHLCGSHLVQALYLVCGERGFFYTPKT	4.9	right-handed G'GT	5	6	7,8	9		56.4	214.2	142.6	44.8	314.2	246.4	168.7	61.9	341.0	289.2	2
1J73	A	B	TR	GIVEQCCTSICSLYQLENYCN	FVNQHLCGSHLVEALYLVCGERGFFYTPKT	4.8	left-handed GGT	5	6	7			24.5	213.2	138.0	49.9	316.7	246.5	170.3	58.1	330.9	279.9	2
1JCA	A	B	TR	GIVEQCCKSICSLYQLENYCN	FVNQHLCGSHLVEALYLVCGERGFFYTPKT	4.7	left-handed GGT	4	6	8			39.0	215.6	133.3	39.4	310.6	242.4	167.0	58.2	329.1	276.9	2
1LPH	A	B	TR	GIVEQCCTSICSLYQLENYCN	FVNQHLCGSHLVEALYLVCGERGFFYTKPT	4.7	left-handed GGT		6	7,8			45.2	213.6	137.9	43.6	314.6	246.7	166.8	65.2	337.4	279.6	2
1M5A	C	D	TT	GIVEQCCTSICSLYQLENYCN	FVNQHLCGSHLVEALYLVCGERGFFYTPKA	4.6	right-handed G'GT	5	6	7,8	9		39.0	217.2	141.3	47.0	315.0	249.5	172.8	65.6	345.7	288.8	2
1MPJ	C	D	TR	GIVEQCCTSICSLYQLENYCN	FVNQHLCGSHLVEALYLVCGERGFFYTPKA	4.7	right-handed G'GT	5	6	7,8			45.1	204.0	137.7	45.7	315.6	249.6	170.2	61.4	335.3	284.2	2
1MSO	C	D	TT	GIVEQCCTSICSLYQLENYCN	FVNQHLCGSHLVEALYLVCGERGFFYTPKT	4.8	right-handed G'GT	5	6	7,8	9		51.9	214.5	140.7	47.3	316.0	314.9	249.5	171.4	65.8	344.8	2
1OS3	C	D	TT	GIVEQCCTSICSLYQLENYCN	FVNQHLCGSHLVEALYLVCGERGFFYTP--	4.7	right-handed G'GT	5	6	7,8	9		56.5	213.7	142.5	48.1	315.4	248.4	169.5	63.2	342.1	287.0	2
1OS4	C	D	TT	GIVEQCCTSICSLYQLENYCN	FVNQHLCGSHLVEALYLVCGERGFFYTP--	4.8	right-handed G'GT		6	7,8			70.5	210.8	146.0	50.2	317.2	251.0	170.5	66.4	346.5	292.0	2
1OS4	G	H	TT	-IVEQCCTSICSLYQLENYCN	FVNQHLCGSHLVEALYLVCGERGFFYTP--	4.6	right-handed G'GT		6	8													2

1OS4	K	L	TT	GIVEQCCTSICSLYQLENYCN	FVNQHLCGSHLVEALYLVCGERGFFYTP--	4.8	right-handed G'GT		6	7,8		48.0	215.9	146.6	44.3	307.3	247.0	164.6	62.8	341.5	285.6	2
1PID	A	B	TT	GIVEQCCASVCSLYQLENYCN	FVNQHLCGSHLVEALYLVCGERGFF-----	4.7	right-handed G'GT	5	6	7,8	9	23.1	218.6	146.0	52.8	318.6	248.3	171.8	63.9	342.5	292.0	2
1PID	C	D	TT	GIVEQCCASVCSLYQLENYCN	FVNQHLCGSHLVEALYLVCGERGFF-----	4.7	right-handed G'GT	5	6	7,8		48.4	214.6	139.6	47.0	315.7	248.1	170.9	64.9	339.0	290.5	2
1Q4V	A	B	TR	GIVEQCCTSICSLYQLENYCN	FVNQHLCGSHLVEALYLVCGERGFFYTPKT	4.7	left-handed GGT	5		7,8		8.2	200.9	134.5	41.8	311.0	243.3	169.5	57.3	333.0	286.3	2
1QJ0	C	D	TR	GIVEQCCTSICSLYQLENYCN	FVNQYLCGSHLVEALYLVCGERGFFYTPKT	5.0	right-handed G'GT	5	6	7,8		45.6	199.7	138.9	47.6	312.2	248.8	168.5	60.2	334.4	287.0	2
1RWE	A	B	TR	GIVEQCCHSICSLYQLENYCN	FVNQHLCGSHLVEALYLVCGERGFFYTPKT	4.7	left-handed GGT	5	6	7,8		49.2	215.6	138.1	46.4	317.4	247.4	170.6	63.3	332.3	280.9	2
1SDB	A	B	T_M	GIVEQCCTSICSLYQLENYCN	--NQHLCGSHLVEALYLVCGERGFF-----	4.9	right-handed G'GT	5	6	7,8	9	29.2	210.6	137.9	45.5	316.2	246.8	168.4	61.1	336.4	285.0	2
1TRZ	A	B	TR	GIVEQCCTSICSLYQLENYCN	FVNQHLCGSHLVEALYLVCGERGFFYTPKT	4.8	left-handed GGT	5	6	7,8	9	63.6	226.1	149.5	57.3	327.8	259.4	180.7	72.6	347.7	297.4	2
1TYL	A	B	TR	GIVEQCCTSICSLYQLENYCN	FVNQHLCGSHLVEALYLVCGERGFFYTPKT	4.8	left-handed GGT		6	7,8		31.3	214.7	139.0	47.4	317.7	248.1	171.4	64.2	336.6	285.6	2
1TYM	A	B	TR	GIVEQCCTSICSLYQLENYCN	FVNQHLCGSHLVEALYLVCGERGFFYTPK-	4.6	left-handed GGT	5	6	7,8	9	24.0	212.3	138.4	46.5	315.8	249.3	171.8	63.7	340.0	287.5	2
1ZNI	C	D	TR	GIVEQCCTSICSLYQLENYCN	FVNQHLCGSHLVEALYLVCGERGFFYTPKA	4.5	left-handed GGT	5	6	7,8		34.6	208.7	136.6	45.2	314.6	248.9	170.3	63.6	336.9	284.4	2
2A3G	C	D	TT	GIVEQCCASVCSLYQLENYCN	FVNQHLCGSHLVEALYLVCGERGFFYTPKA	4.8	right-handed G'GT	5	6	7,8	9	51.8	218.2	146.8	53.8	318.3	246.8	170.4	61.4	340.2	287.9	2
2BN3	A	B	T_M	GIVEQCCTSVCSLYQLENYCN	FVNQHLCGSHLVEALYLVCGERGFFYTPKA	3.9	right-handed hook	5	6	7,8		38.8	215.1	140.3	45.5	315.7	248.6	171.4	66.5	338.5	338.2	2
2C8Q	A	B	T_M	GIVEQCCTSICSLYQLENYCN	FVNQHLCGSHLVEALYLVCGERGFFYTPK-	3.9	right-handed hook	5	6	7,8		38.5	215.6	141.0	47.2	316.3	248.1	171.8	66.2	337.3	288.8	2
2CEU	A	B	TT	GIVEQCCTSICSLYQLENYCN	-VNQHLCGSHLVEALYLVCGERGFF-----	4.6	right-handed G'GT	5	6	7,8	9	39.3	217.9	145.2	50.0	315.0	247.1	168.0	60.3	339.0	288.8	2
2CEU	C	D	TT	GIVEQCCTSICSLYQLENYCN	-VNQHLCGSHLVEALYLVCGERGFF-----	4.6	right-handed G'GT	5	6	7,8	9	38.1	217.3	145.2	50.0	314.6	247.1	167.9	60.4	339.0	288.8	2
2EFA	A	B	T_M	GIVEQCCTSICSLYQLENYCN	FVNQHLCGSHLVEALYLVCGERGFFYTPKA	3.8	right-handed hook	5	6	7,8		44.0	215.7	140.4	46.8	316.6	250.2	173.7	69.6	340.8	287.5	2
2G4M	A	B	T_M	GIVEQCCTSICSLYQLENYCN	FVNQHLCGSHLVEALYLVCGERGFFYTPKA	3.9	right-handed hook	5	6	7,8		37.1	215.1	140.6	46.9	317.6	248.0	171.7	65.9	337.5	288.6	2
2QIU	C	D	TR	GIVEQCCTSICSLYQLENYCN	FVNQHLCGSHLVEALYLVCGERGFFYTPKT	5.0	right-handed G'GT	5	6	7,8		47.2	198.0	141.8	48.5	314.9	249.2	172.5	61.7	330.9	287.7	2
2R34	C	D	TR	GIVEQCCTSICSLYQLENYCN	FVNQHLCGSHLVEALYLVCGERGFFYTPK-	4.5	left-handed GGT	5	6	7,8	9	50.0	206.2	139.7	51.3	316.4	251.2	175.5	59.5	338.2	290.4	2
2R36	A	B	TT	GIVEQCCTSICSLYQLENYCN	FVNQHLCGSHLVEALYLVCGERGFFYTPKT	5.1	right-handed G'GT		6	7,8		33.9	206.4	139.8	50.2	318.3	246.8	168.6	59.2	332.8	285.4	2
2R36	C	D	TT	GIVEQCCTSICSLYQLENYCN	FVNQHLCGSHLVEALYLVCGERGFFYTPKT	4.9	right-handed G'GT		6	7,8		44.7	210.1	140.2	51.2	318.7	248.3	171.5	60.9	338.7	291.3	2
2TCI	C	D	TR	GIVEQCCTSICSLYQLENYCN	FVNQHLCGSHLVEALYLVCGERGFFYTPKA	4.7	right-handed G'GT	5	6	7,8		59.3	210.0	137.6	46.6	317.3	250.2	172.8	65.7	338.2	287.7	2
2VJZ	A	B	TR	GIVEQCCTSICSLYQLENYCN	FVNQHLCGSHLVEALYLVCGERGFFYTPK-	4.5	left-handed GGT	5	6	7,8	9	38.4	207.5	136.0	44.0	314.6	314.2	248.5	170.7	63.4	337.6	2
2VK0	A	B	TT	GIVEQCCTSICSLYQLENYCN	--NQHLCGSHLVEALYLVCGERGFFYTPKT	4.7	left-handed hook	5	6	7,8		65.8	206.2	136.8	42.4	311.3	248.2	169.2	64.9	337.9	289.0	2
2WRU	A	B	T_M	GIVEQCCTSICSLYQLENYCN	-VNQHLCGSHLVEALYLVCGERGFF-----	4.6	right-handed G'GT	5	6	7,8	9	35.8	212.8	137.0	45.2	313.6	247.1	168.6	62.3	339.4	288.3	2
2WRV	A	B	T_M	GIVEQCCTSICSLYQLENYCN	-VNQHLCGSHLVEALYLVCGERGFF-----	4.7	right-handed G'GT	5	6	7,8	9	40.1	210.7	137.1	45.6	314.4	247.6	169.9	62.1	337.8	290.2	2
2WRW	A	B	T_M	GIVEQCCTSICSLYQLENYCN	-VNQHLCGSHLVEALYLVCGERGFF-----	4.6	right-handed G'GT	5	6	7,8	9	48.2	212.2	137.9	45.5	315.9	247.8	170.1	64.5	338.6	288.6	2
2WRX	A	B	TT	GIVEQCCTSICSLYQLENYCN	-VNQHLCGSHLVEALYLVCGERGFF-TP--	4.7	right-handed G'GT	5	6	7,8	9	33.3	214.3	137.0	44.9	313.8	246.1	168.5	62.1	338.5	288.5	2
2WRX	C	D	TT	GIVEQCCTSICSLYQLENYCN	-VNQHLCGSHLVEALYLVCGERGFF-TP--	4.6	right-handed G'GT	5	6	7,8	9	33.9	215.0	137.6	46.1	315.0	247.8	168.7	62.9	339.6	288.9	2
2WS0	A	B	T_M	GIVEQCCTSICSLYQLENYCN	FVNQHLCGSHLVEALYLVCGERGFF-T---	4.7	right-handed G'GT	5	6	7,8	9	46.2	210.8	136.9	45.7	315.5	247.3	171.8	63.5	337.8	289.1	2
2WS1	A	B	T_M	GIVEQCCTSICSLYQLENYCN	-VNQHLCGSHLVEALYLVCGERGFF-TP--	4.6	right-handed G'GT	5	6	7,8	9	33.8	212.0	136.5	43.6	312.8	245.7	167.2	60.9	337.6	286.8	2
2WS4	A	B	T_M	GIVEQCCTSICSLYQLENYCN	-VNQHLCGSHLVEALYLVCGERGFFP----	4.6	right-handed G'GT	5	6	7,8	9	42.5	215.5	141.3	47.2	316.4	248.6	170.6	63.3	339.5	285.8	2
2ZP6	C	D	TT	GIVEQCCASVCSLYQLENYCN	FVNQHLCGSHLVEALYLVCGERGFFYTPKA	4.9	right-handed G'GT	4,5	6	7,8	9	4.8	215.4	141.1	41.1	312.2	241.2	164.9	60.7	335.8	284.2	2
2ZPP	A	B	TT	GIVEQCCTSICSLYQLENYCN	FVNQHLCGSHLVEALYLVCGERGFFYTPKA	3.9	right-handed hook	4,5	6	7,8		61.8	216.5	143.3	47.8	314.3	249.0	171.4	67.7	342.8	291.0	2
3BXQ	C	D	TT	GIVEQCCTSICSLYQLENYCN	FVNQRLCGSHLVEALYLVCGERGFFYTPKT	4.8	right-handed G'GT			6,7		56.8	217.9	148.8	50.1	318.6	243.5	168.2	62.2	337.3	288.4	2
3E7Y	A	B	TT	GIVEQCCTSICSLYQLENYCN	FVNQHLCGSHLVEALYLVCGERGFFYTPK-	4.6	right-handed G'GT	5	6	7,8		45.4	218.5	142.9	47.1	313.1	248.3	171.1	64.8	342.2	288.5	2
3E7Z	A	B	TT	GIVEQCCTSICSLYQLENYCN	FVNQHLCGSHLVEALYLVCGERGFFYTPK-	4.6	right-handed G'GT	5	6	7,8		45.3	219.3	143.3	47.0	311.1	248.8	170.6	63.7	342.2	288.7	2
3FHP	C	D	TT	GIVEQCCTSICSLYQLENYCN	FVNQHLCGSHLVEALYLVCGERGFFYTPKA	4.8	right-handed G'GT	5	6	7,8	9	57.0	215.4	144.6	49.6	317.3	247.2	169.2	64.2	342.2	289.5	2
3FQ9	C	D	TT	-IVEQCC-SICSLYQLENYCN	FVNQHLCGSHLVEALYLVCGERGFFYTPKT	4.6	right-handed G'GT		6	7		41.0	203.2	130.5	36.6	304.4	234.3	157.7	51.1	330.4	277.5	2
3GKY	A	B	TR	GIVEQCCHSICSLYQVENYCN	FVNQHLCGSHLVEALYLVCGERGFFYTPKA	4.8	right-handed G'GT	5	6	7,8		28.9	191.8	115.4	24.9	293.5	221.5	145.7	36.2	305.2	256.7	2

**3I3Z**	A	B	T_M	GIVEQCCTSICSLYQLENYCN	FVNQHLCGSHLVEALYLVCGERGFFYTPKA	3.9	right-handed hook	5	6	7,8		50.4	213.7	140.5	46.7	315.8	250.1	171.9	67.1	338.2	288.6	2
3I40	A	B	T_M	GIVEQCCTSICSLYQLENYCN	FVNQHLCGSHLVEALYLVCGERGFFYTPKA	3.9	right-handed hook	5	6	7,8		47.1	216.0	140.9	44.9	314.1	248.3	169.4	65.5	338.5	288.5	2
3ILG	C	D	TT	GIVEQCCTSICSLYQLENYCN	FVNQHLCGSHLVEALYLVCGERGFFYTPKT	4.7	right-handed G'GT	5	6	7,8	9	44.2	214.7	139.7	45.4	312.5	248.1	170.9	65.6	344.3	287.9	2
3INC	C	D	TT	GIVEQCCTSICSLYQLENYCN	FVNQHLCGSHLVEALYLVCGERGFFYTPKT	4.7	right-handed G'GT	5	6	7,8	9	50.6	212.8	140.4	46.9	313.4	248.2	171.0	64.4	341.7	287.8	2
3INS	C	D	TT	GIVEQCCTSICSLYQLENYCN	FVNQHLCGSHLVEALYLVCGERGFFYTPKA	4.7	right-handed G'GT	5	6	7,8	9	64.1	229.7	156.2	60.5	328.5	262.4	185.0	79.4		303.9	2
3IR0	C	D	TT	GIVEQCCTSICSLYQLENYCN	FVNQHLCGSHLVEALYLVCGERGFFYTPKT	4.9	right-handed G'GT	4,5	6	8		76.9	204.8	142.1	38.2	310.4	254.1	175.6	66.1	348.4	294.9	2
3IR0	G	H	TT	GIVEQCCTSICSLYQLENYCN	FVNQHLCGSHLVEALYLVCGERGFFYTPKT	4.9	right-handed G'GT	5	6	8	9	56.5	205.1	140.0	44.5	312.7	247.2	173.5	63.9	340.9	287.7	2
3IR0	K	L	TT	GIVEQCCTSICSLYQLENYCN	FVNQHLCGSHLVEALYLVCGERGFFYTPKT	4.9	right-handed G'GT	5	6	7,8		52.6	194.1	128.0	36.5	307.2	245.4	164.4	56.8	335.4	288.1	2
3IR0	O	P	TT	GIVEQCCTSICSLYQLENYCN	FVNQHLCGSHLVEALYLVCGERGFFYTPKT	4.9	right-handed G'GT	5	6	7,8		45.8	195.1	130.1	37.7	308.6	245.8	165.5	56.8	335.9	288.5	2
3IR0	T	U	TT	GIVEQCCTSICSLYQLENYCN	FVNQHLCGSHLVEALYLVCGERGFFYTPKT	4.9	right-handed G'GT	4,5	6	8		54.3	214.6	147.0	52.2	317.9	252.4	182.5	70.1	346.2	293.3	2
3IR0	X	Y	TT	GIVEQCCTSICSLYQLENYCN	FVNQHLCGSHLVEALYLVCGERGFFYTPKT	5.0	right-handed G'GT	4	5,6			31.3	228.2	145.5	55.2	317.2	252.0	172.0	61.9	347.6	296.2	2
3JSD	A	B	TR	GIVEQCCTSICSLYQLENYCN	FVNQHLC-SHLVEALYLVCGERGFFYTPKT	4.9	left-handed GGT	5	6	7,8		45.7	218.0	140.7	50.9	314.8	245.7	170.9	55.6	329.4	286.1	2
3KQ6	A	B	TR	GIVHQCCHSICSLYQLENYCN	FVNQHLCGSHLVEALYLVCGERGFFYTPKT	4.8	left-handed GGT	5	6	7,8	9	54.9	221.0	144.0	53.0	318.9	250.7	170.3	62.2	336.8	287.0	2
3MTH	C	D	TR	GIVEQCCTSICSLYQLENYCN	FVNQHLCGSHLVEALYLVCGERGFFYTPKA	4.4	left-handed GGT	5	6	7,8		49.8	206.7	135.9	45.2	315.7	249.4	170.8	63.8	337.3	287.0	2
3Q6E	C	D	TT	GIVEQCCTSICSLYQLENYCN	FVNQHLCGSHLVEALYLVCGERGFFYTPKT	4.7	right-handed G'GT	5	6	7,8	9	39.2	212.3	138.1	43.9	312.6	247.1	167.4	63.0	338.9	288.7	2
3RTO	C	D	TT	GIVEQCCTSICSLYQLENYCN	FVNQHLCGSHLVEALYLVCGERGFFYTPKA	4.7	right-handed G'GT	5	6	7,8	9	36.6	213.7	141.9	49.0	315.4	246.1	170.0	60.5	341.2	289.6	2
4INS	C	D	TT	GIVEQCCTSICSLYQLENYCN	FVNQHLCGSHLVEALYLVCGERGFFYTPKA	4.7	right-handed G'GT	5	6	7,8	9	40.6	214.2	141.1	46.4	313.2	247.6	170.9	63.7	341.4	288.0	2
9INS	A	B	T_M	GIVEQCCTSICSLYQLENYCN	FVNQHLCGSHLVEALYLVCGERGFFYTPKA	3.9	right-handed hook	5	6	7,8		44.1	213.3	140.8	46.6	317.1	248.3	171.4	65.7	340.3	290.3	2

Supplementary Table S4. Average azimuthal angles ϕ (in degrees) for each conformation class. Values are mean over all unique X-ray crystal structures used in the bioinformatics study (**Supplementary Table S3**) for each of the ten residues of the A-chain *N*-terminal helix.

	Residue									
	**A1**	**A2**	**A3**	**A4**	**A5**	**A6**	**A7**	**A8**	**A9**	**A10**
**Class 1**	27±10	265±16	177±25	97±23	323±65	256±23	177±20	59±31	313±73	291±12
**Class 2**	45±12	213±7	140±6	47±6	315±6	250±13	173±15	67±20	326±60	291±13

Supplementary Table S5. Mean distances (Å) between the Cα atoms of the A6-A11 linkage from X-ray crystal structures and MD simulations.

**A6-A11**	**Class 1**	**Class 2**
X-ray – insulin	4.78 ± 0.13	4.55 ± 0.37
X-ray – insulin bound to μIR	-	4.50 ± 0.15
X-ray – *trans* isomer	5.17 ± 0.13	-
MD – insulin	4.86 ± 0.46	3.97 ± 0.18
MD – *cis* isomer	4.42 ± 0.31	4.05 ± 0.27
MD – *trans* isomer	5.18 ± 0.22	-

**A7-B7**		
X-ray – insulin	4.62 ± 0.13	4.76 ± 0.11
X-ray – insulin bound to μIR	-	5.53
X-ray – *trans* isomer	4.36	-
MD – insulin	4.36±0.28	4.76±0.20
MD – *cis* isomer	4.47±0.30	4.81±0.21
MD – *trans* isomer	4.42±0.27	-

Supplementary Table S6. The percentage of frames in the molecular dynamics simulations where the NH of residue Thr^{A8} forms hydrogen bonds with the carbonyl group of residues Val^{A3}, Glu^{A4} and Gln^{A5}. Detail of the hydrogen bond formation criterion is provided in **Supplementary Methods** (q.v.).

Thr^{A8} hydrogen bonding partner (C=O)	Insulin	*cis* isomer	*trans* isomer
Val^{A3}	4 ± 8	4 ± 8	0
Glu^{A4}	55 ± 9	48 ± 20	52 ± 20
Gln^{A5}	32 ± 10	29 ± 15	32 ± 16

Supplementary Table S7. The conformation of various regions of structure, based on MD simulations. Secondary structure types were assigned to each residue in all simulation frames using DSSP (Kabsch, W. and Sander, C. (1983) Dictionary of protein secondary structure - pattern recognition of hydrogen-bonded and geometrical features. Biopolymers, 22(12) 2577-2637). The percentage of frames in which each residue adopted each structure type was determined across all simulations, then the values averaged over all residues in the relevant region to obtain the final results. Where the deviations of the values for the dicarba insulin isomers from those of insulin are statistically significant at the 95% confidence limit (Student's t-test), these numbers are highlighted in **bold**. HB=hydrogen bonded; nHB= non-hydrogen bonded.

		Insulin	*cis* isomer	*trans* isomer
A3-A8	αhelix	50	**39**	**32**
	3_{10} helix	3	7	5
	π helix	0	0	0
	HB turn	38	39	**45**
	nHB turn	7	9	**11**
	other	1	5	7
A13-A19	α helix	38	**31**	36
	3_{10} helix	16	19	17
	π helix	0	0	0
	HB turn	34	**39**	38
	nHB turn	11	11	10
	other	0	0	0
B29-B40	α helix	72	**64**	**69**
	3_{10} helix	3	5	4
	π helix	0	0	0
	HB turn	16	**22**	18
	nHB turn	9	10	10
	other	0	0	0

Supplementary Figure S1a

***des$_{A1\text{-}5}$*-[A6,11]-Agl-[A7]-Cys(^{t}Bu)-[A20]-Cys(Acm) human insulin A chain 1**

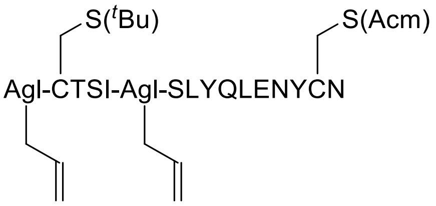

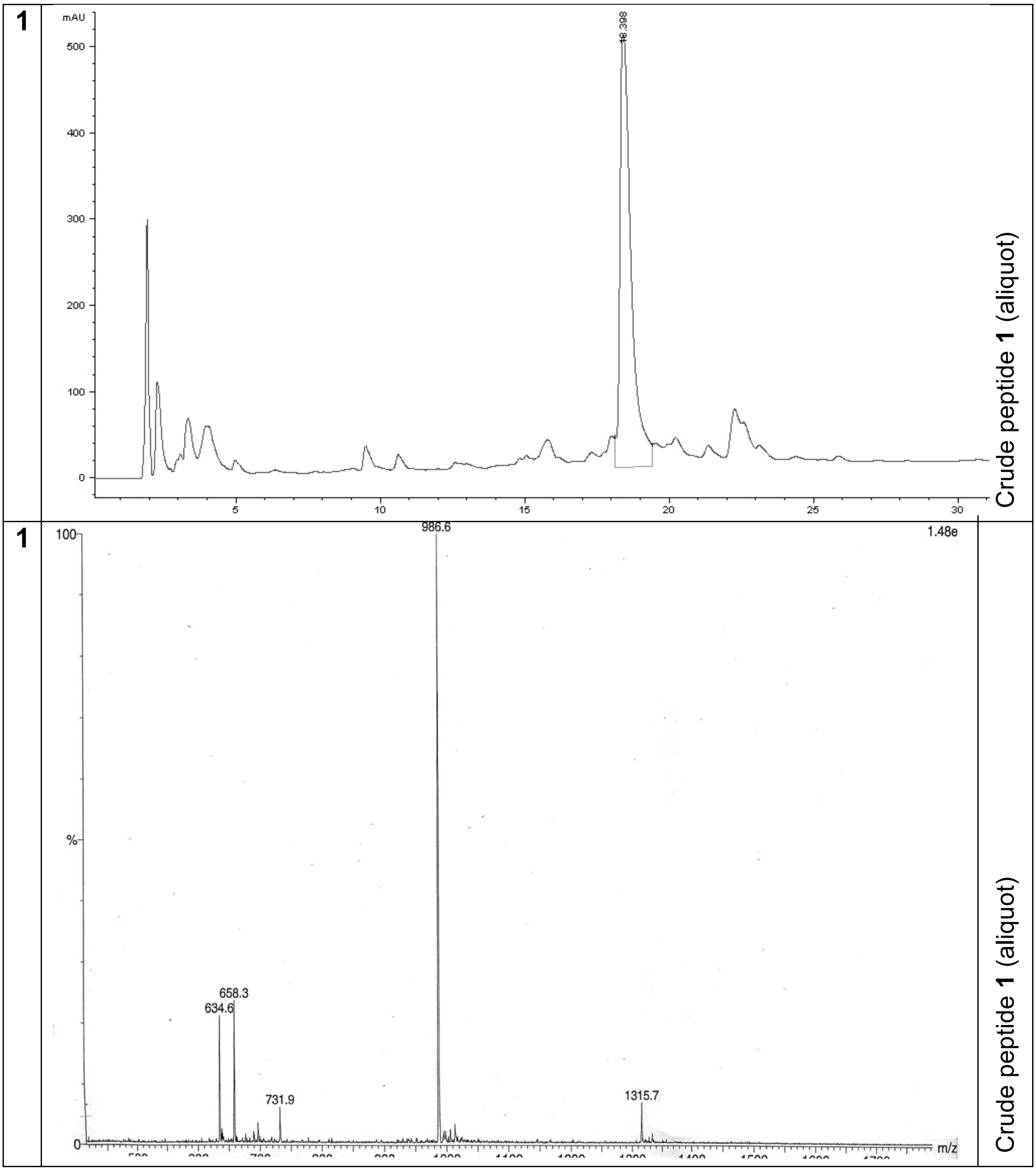

Supplementary Figure S1b

***des$_{A1-5}$-c*[Δ^{4}A6,11]-Dicarba-[A7]-Cys(^{t}Bu)-[A20]-Cys(Acm) human insulin A chain 2**

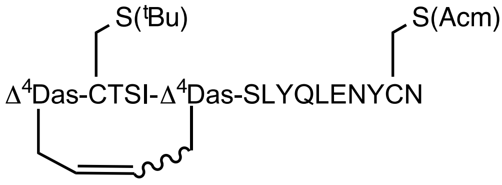

2

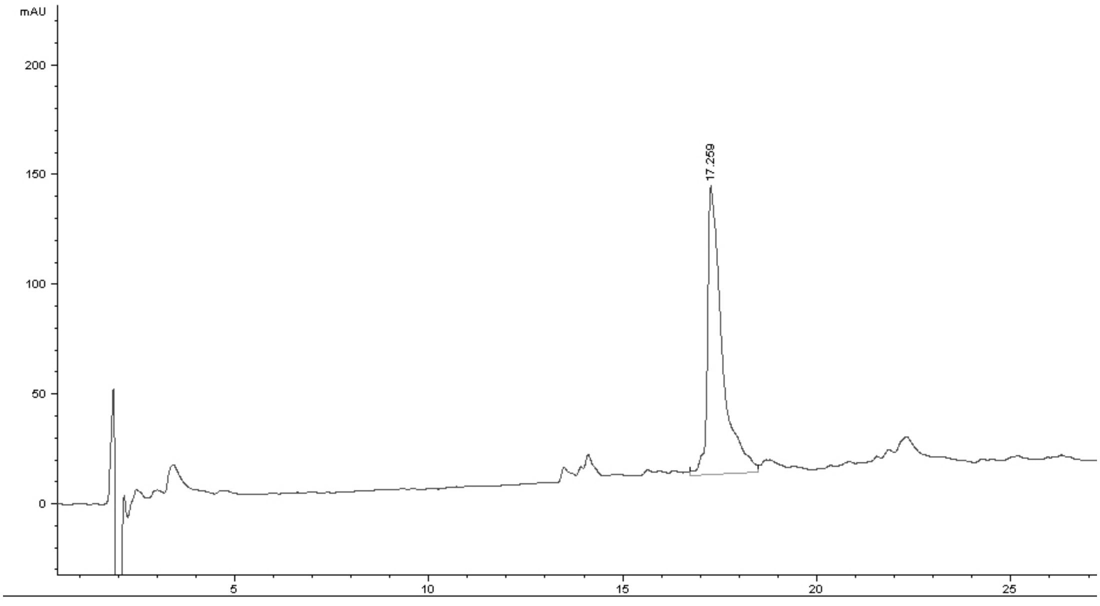

Crude peptide **2** (aliquot)

2

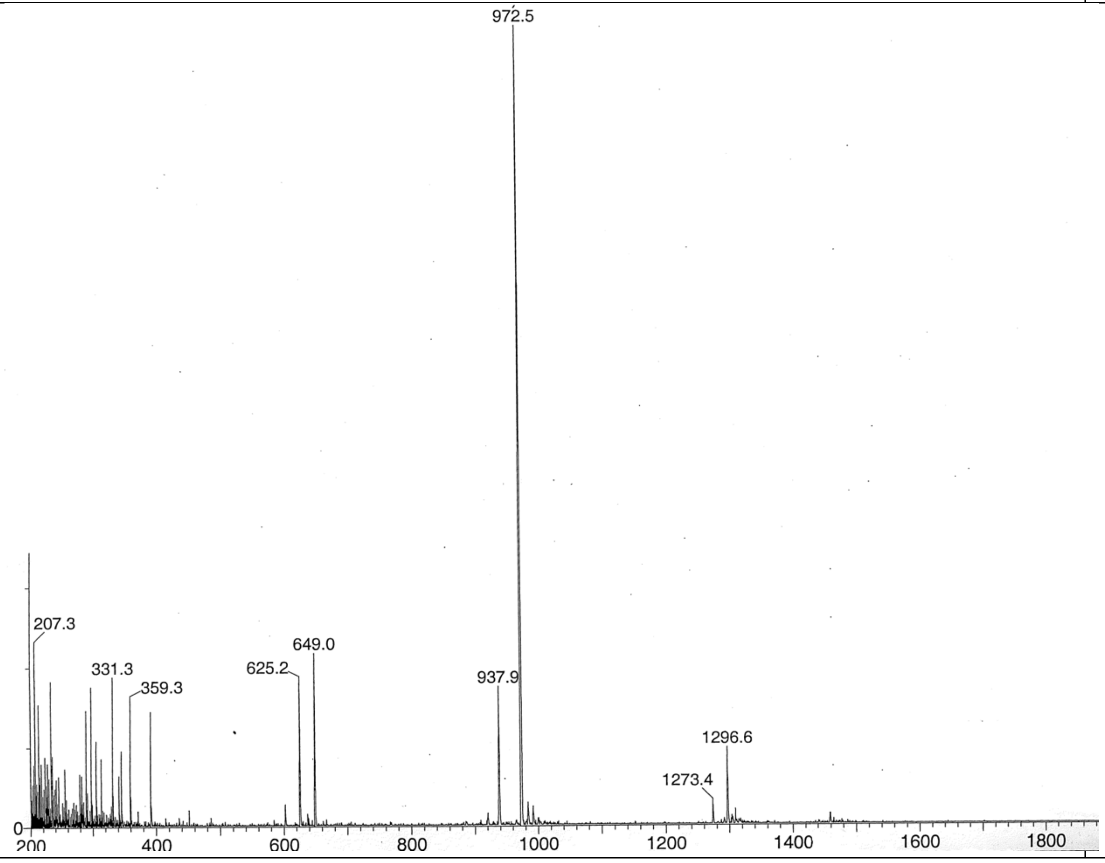

Crude peptide **2** (aliquot)

Supplementary Figure S1c

***c*[Δ^4A6,11]-Dicarba-[A7]-Cys(^{t}Bu)-[A20]-Cys(Acm) human insulin A chain *Z*-3**

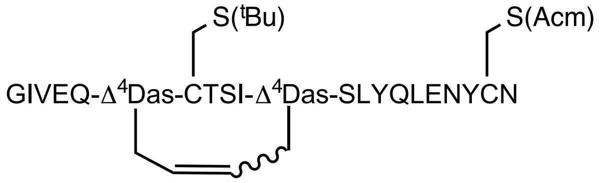

**3**	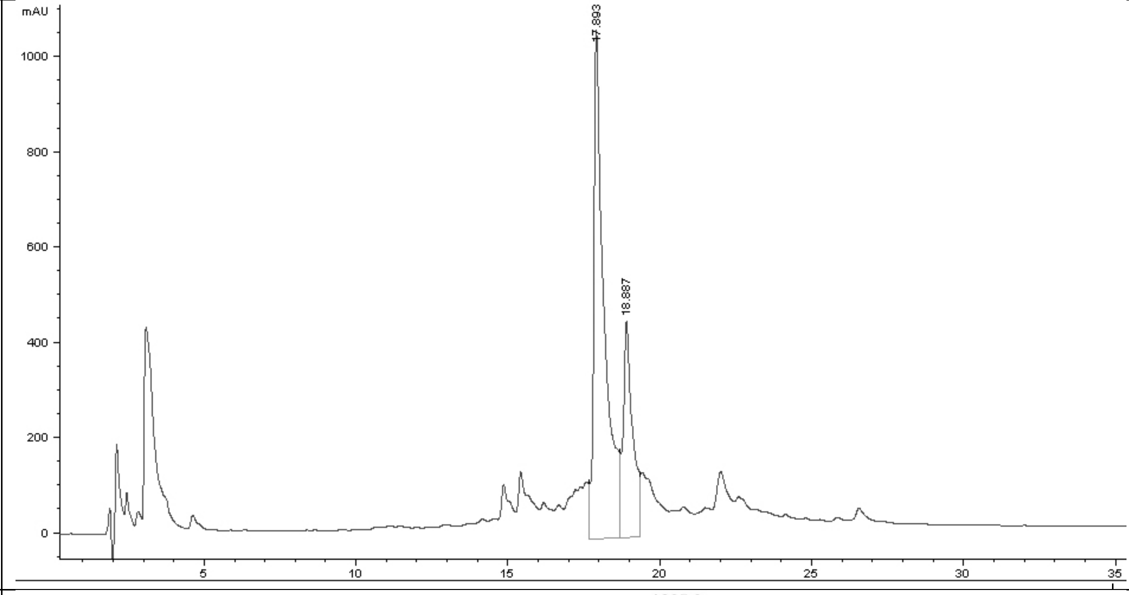 Crude ***E/Z*-3** (aliquot); t_R = 17.9 min (***Z*-3**)
**3**	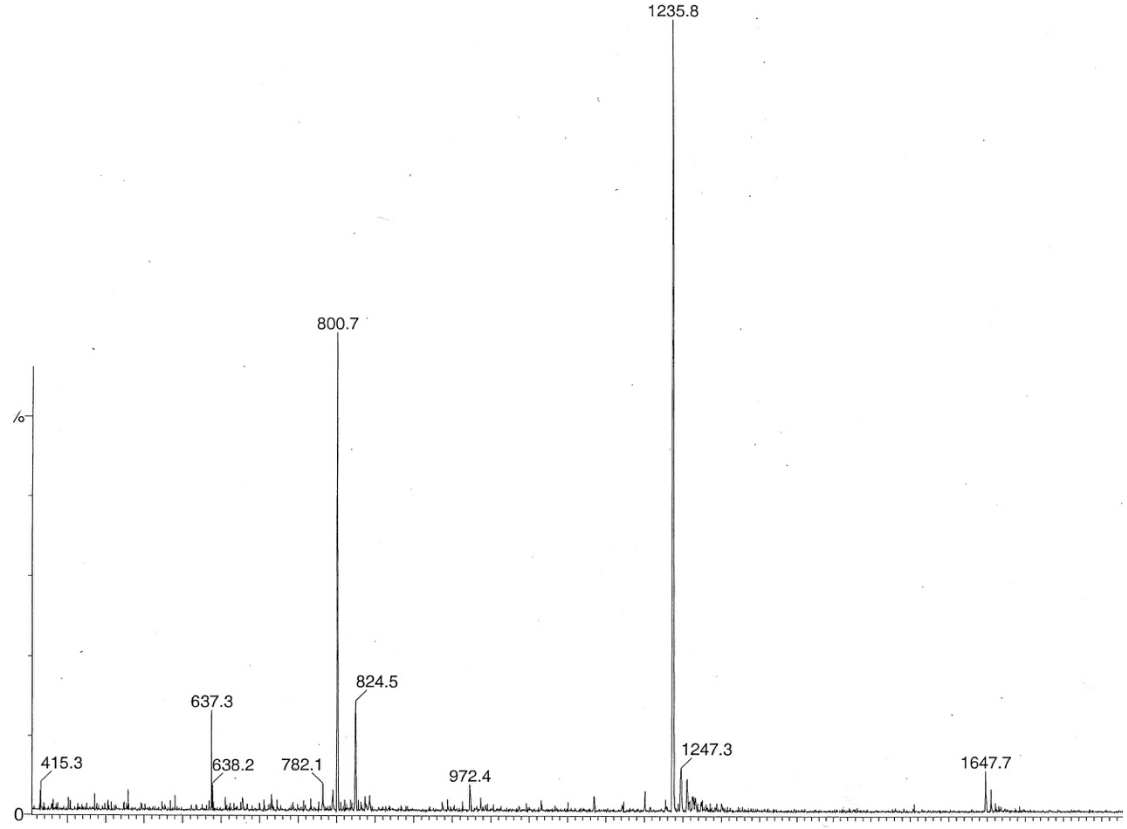 Crude peptide ***Z*-3** (aliquot)

Supplementary Figure S1d

***c*[Δ^{4}A6,11]-Dicarba-[A7]-Cys(^{t}Bu)-[A20]-Cys(Acm) human insulin A chain *E*-3**

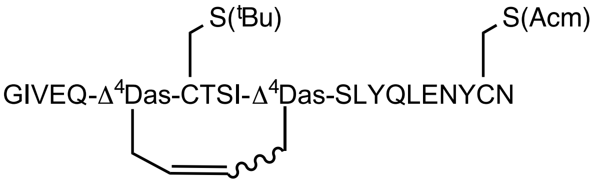

3

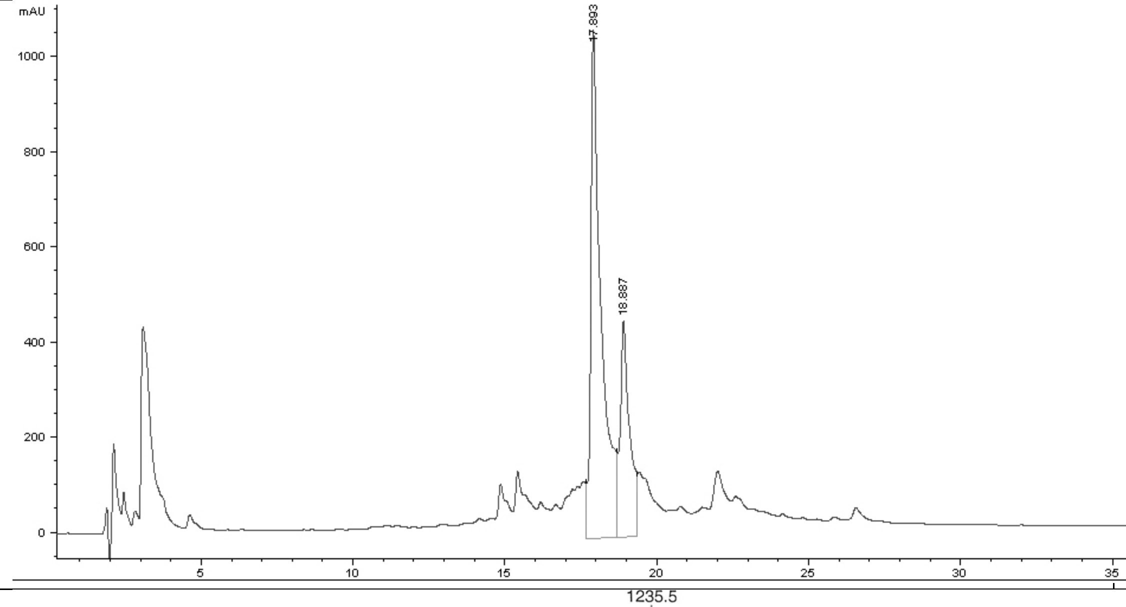

Crude ***E/Z*-3** (aliquot); t_R = 18.9 min (***E*-3**)

3

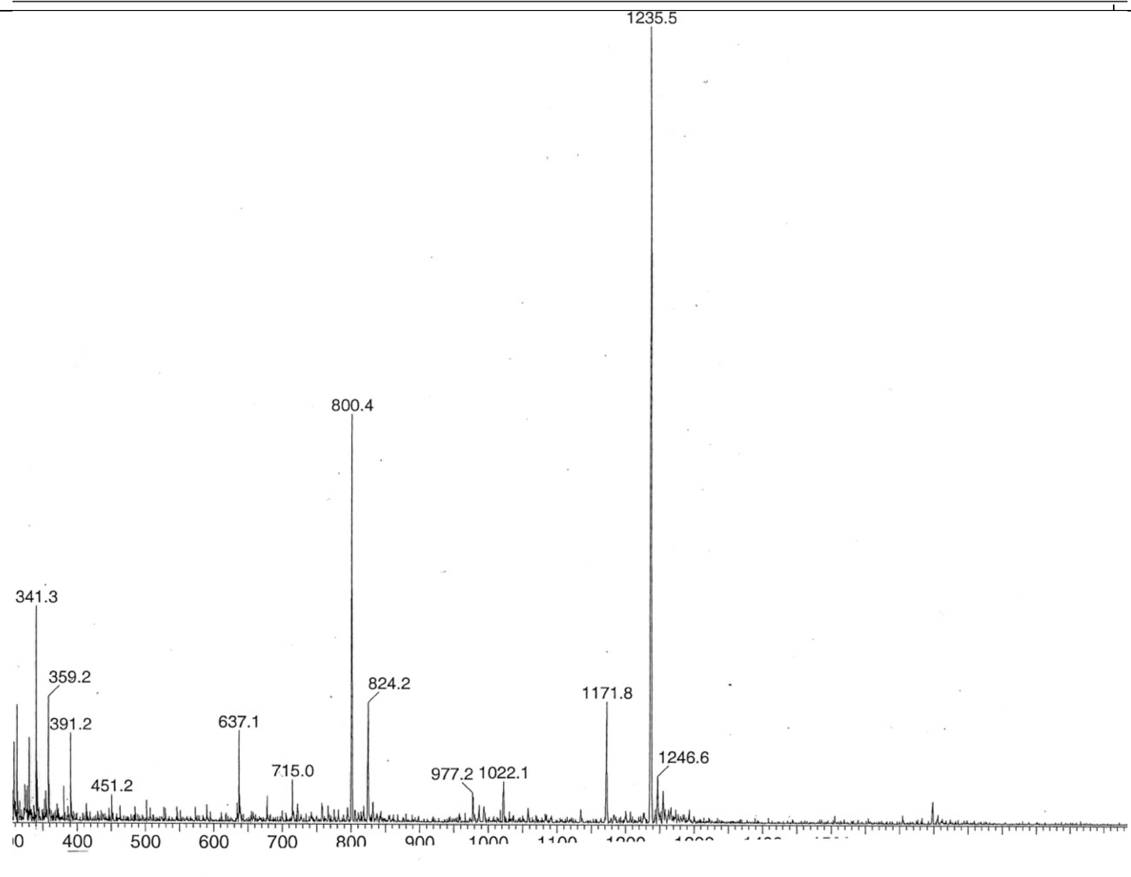

Crude peptide ***E*-3** (aliquot)

Supplementary Figure S1e

***c*[Δ^{4}A6,11]-Dicarba-[A7]-Cys(Pyr)-[A20]-Cys(Acm) human insulin A chain *Z*-4**

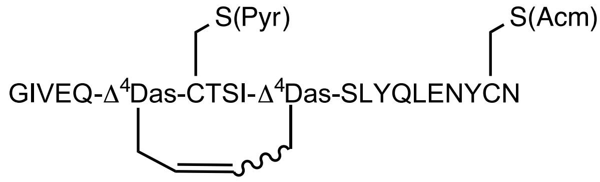

4

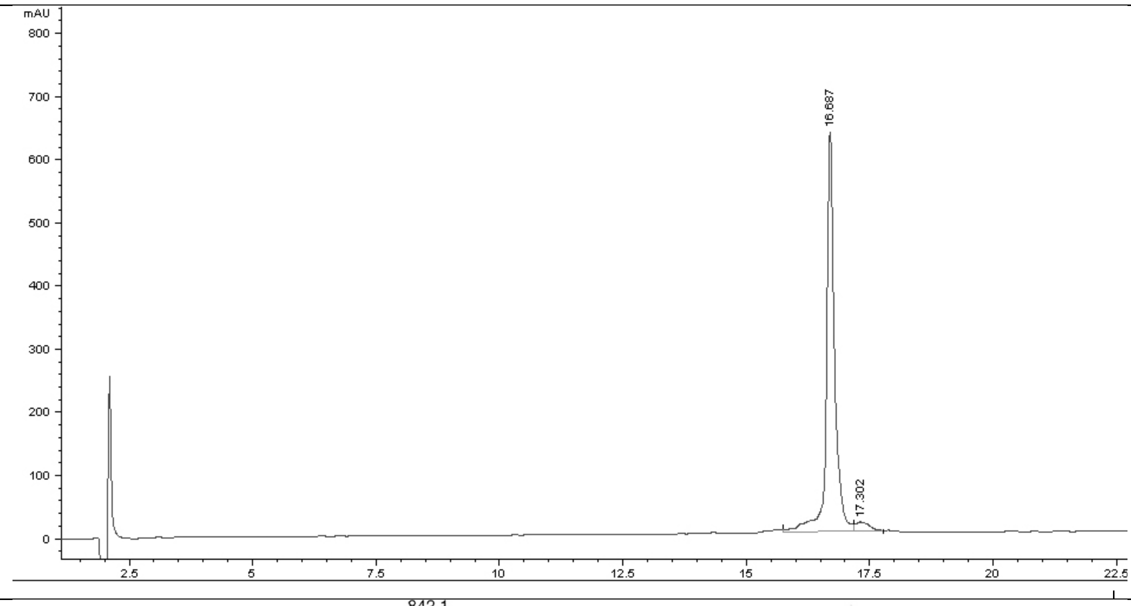

Purified **Z-4**; t_R = 16.7 min

4

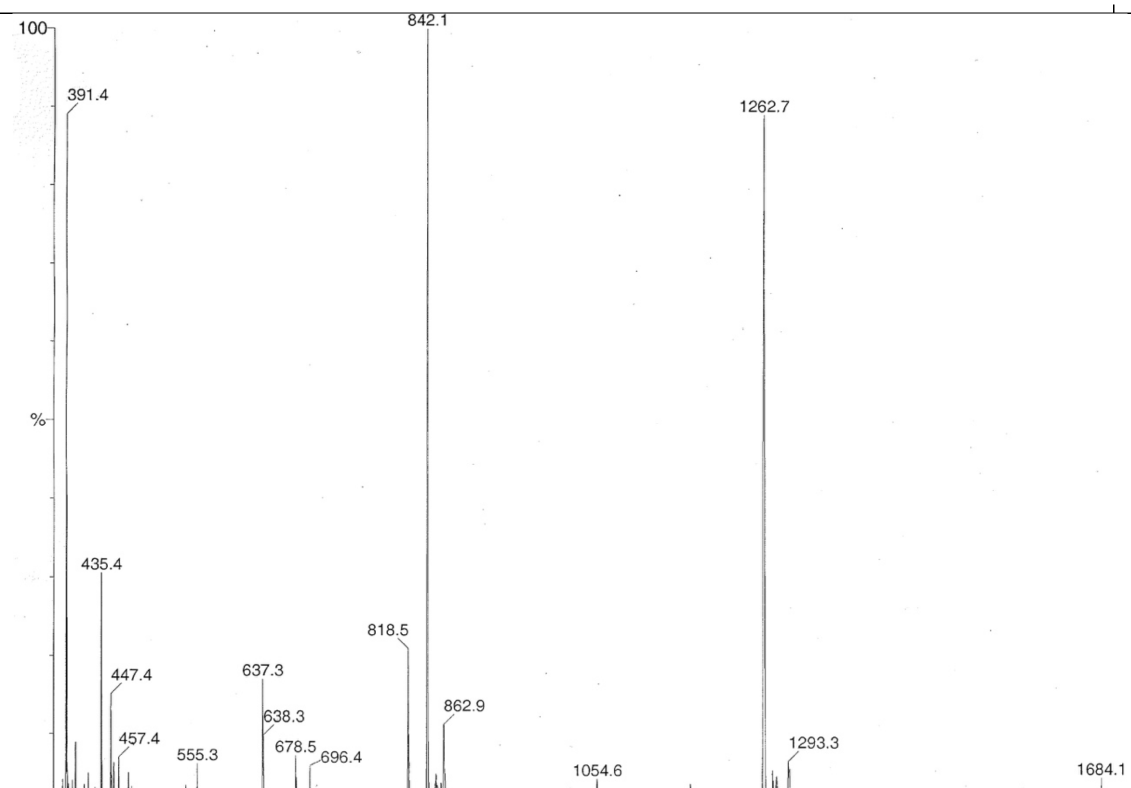

Purified **Z-4**

Supplementary Figure S1f

***c*[Δ^{4}A6,11]-Dicarba-[A7]-Cys(Pyr)-[A20]-Cys(Acm) human insulin A chain *E*-4**

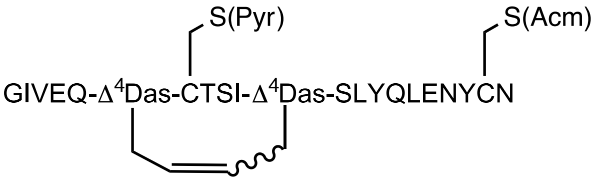

4

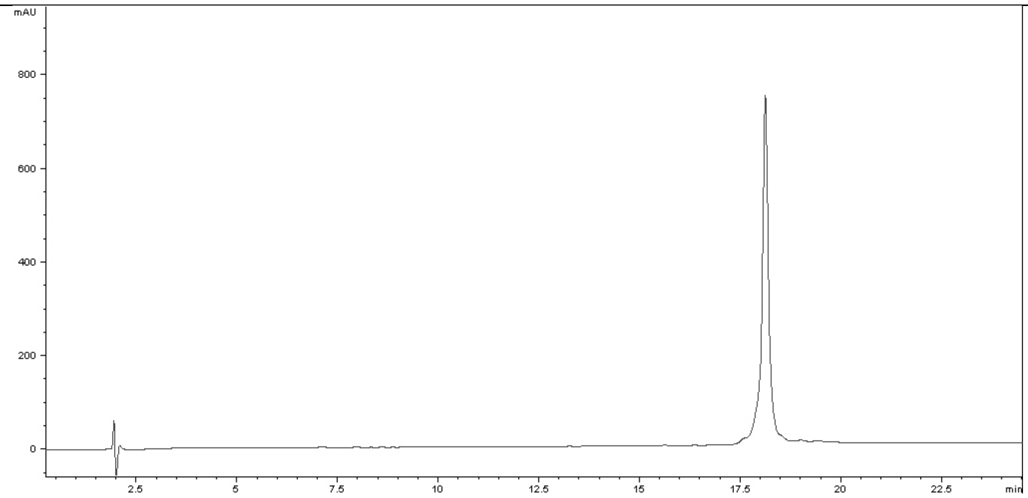

Purified ***E-4***; t_R = 18.2 min

4

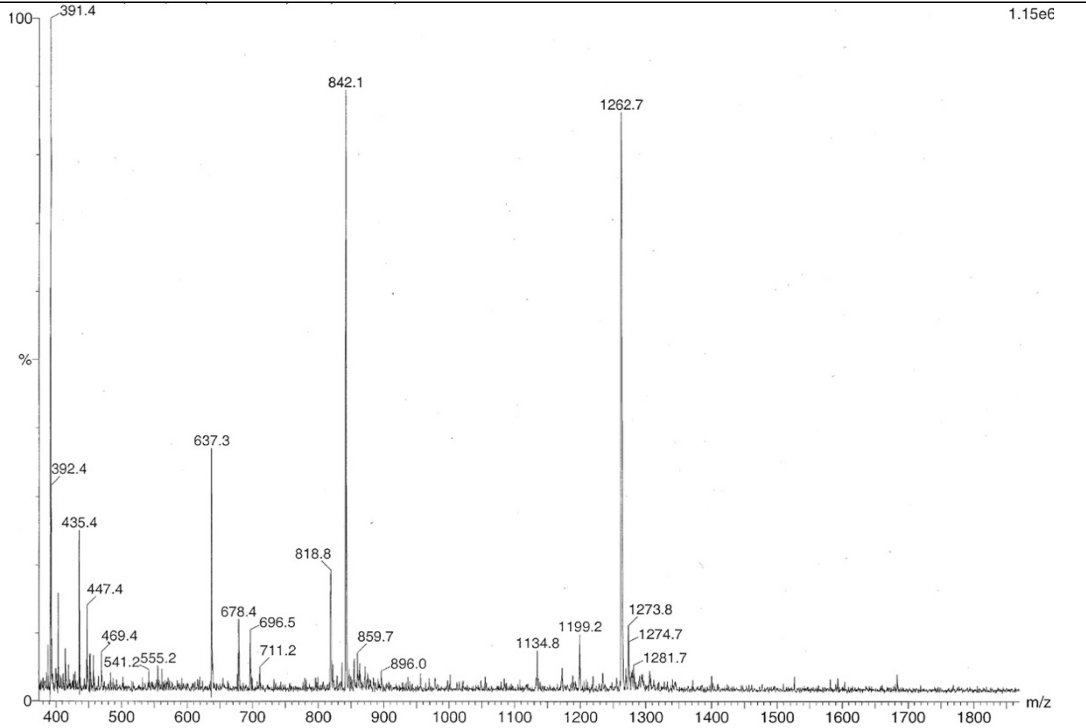

Purified peptide ***E-4***

Supplementary Figure S1g

[B19]-Cys(Acm) human insulin B chain 5

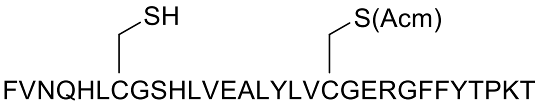

**5**	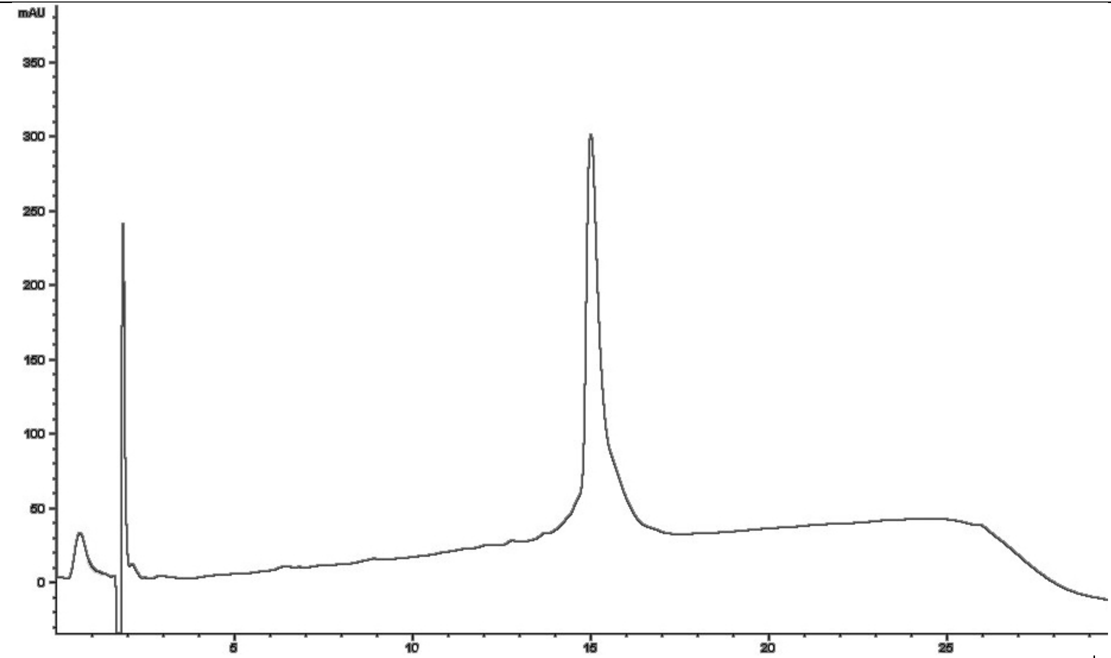	Purified peptide **5**; t_R = 15.0 min.
**5**	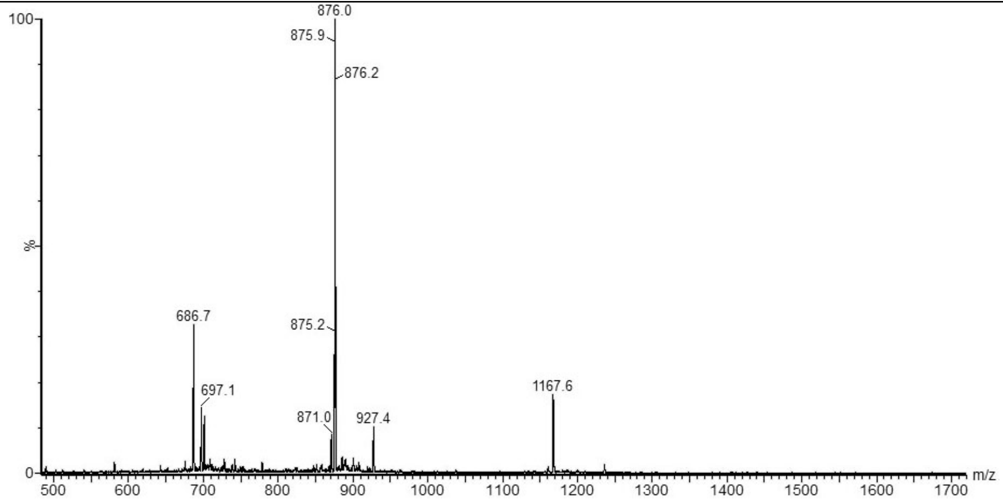	Purified peptide **5**

Supplementary Figure S1h

Monocyclic A-B heterodimer of *c*[Δ^4A6,11]-dicarba human insulin *Z*-6

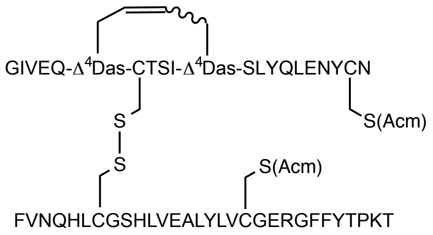

6

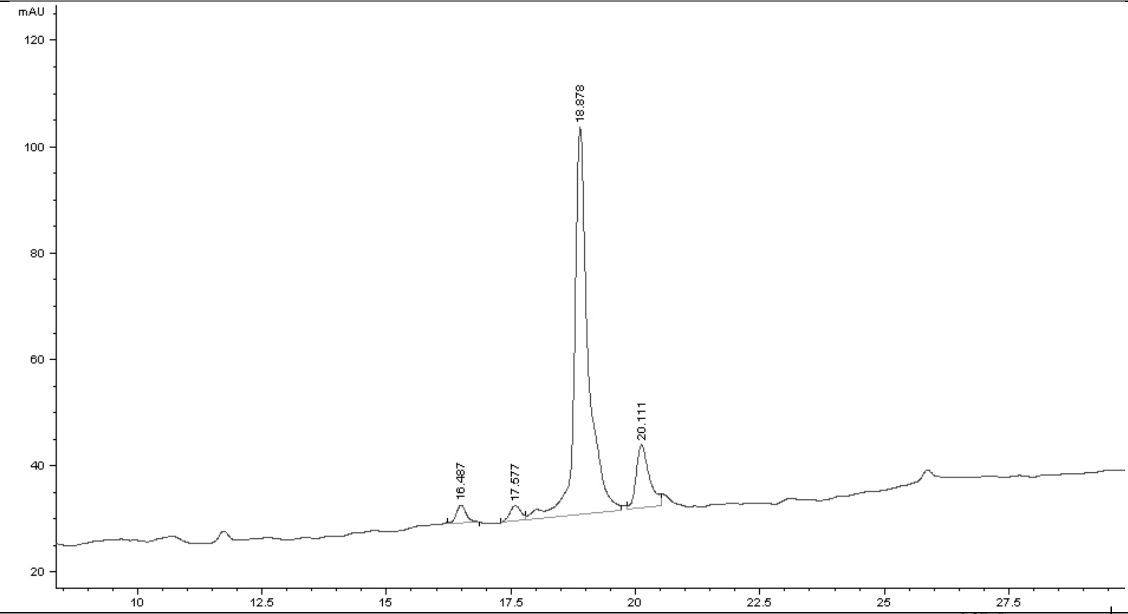

Crude peptide **Z-6**; t_R = 18.9 min

6

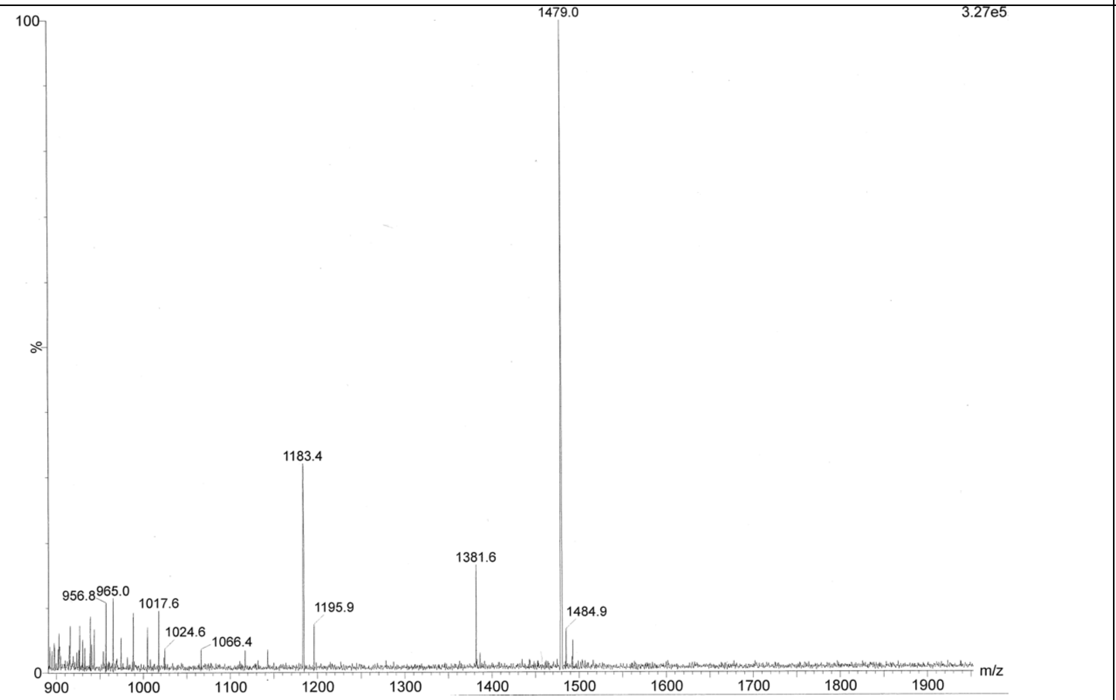

Peptide **Z-6**

Supplementary Figure S1i

Monocyclic A-B heterodimer of *c*[Δ[4]A6,11]-dicarba human insulin chain *E*-6

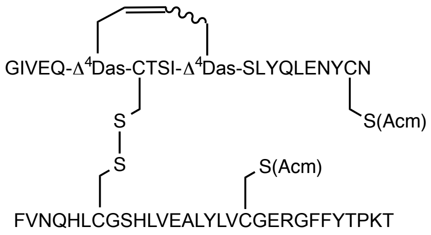

**6**	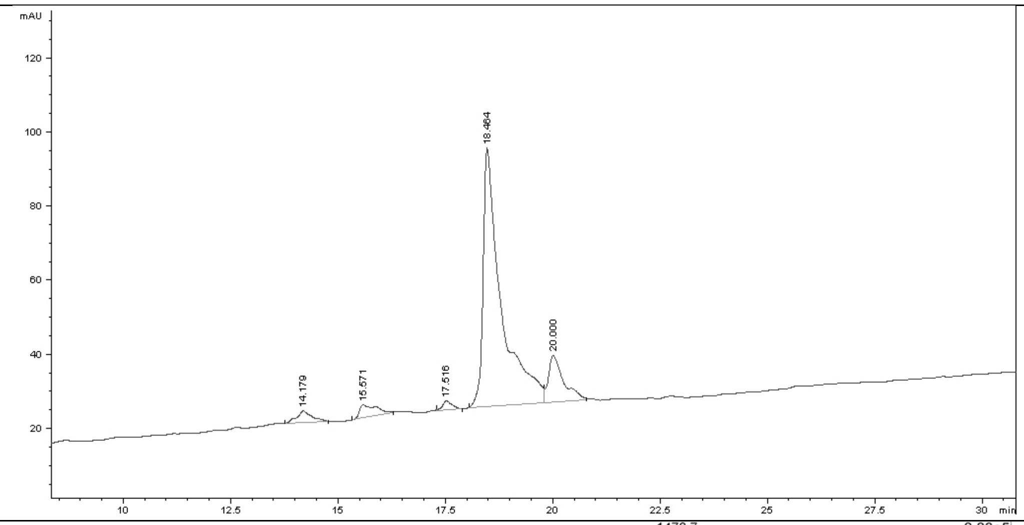	Crude peptide ***E*-6**; t_R = 18.5 min
**6**	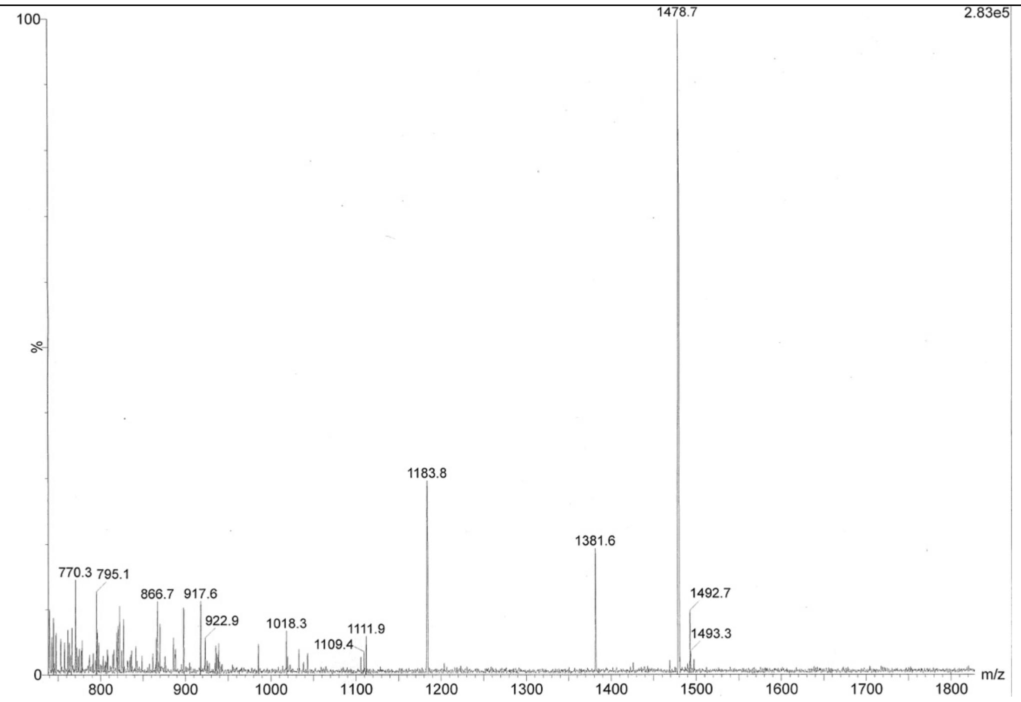	Purified peptide ***E*-6**

Supplementary Figure S1j

***c*[Δ^{4}A6,11]-Dicarba human insulin *Z*-7**

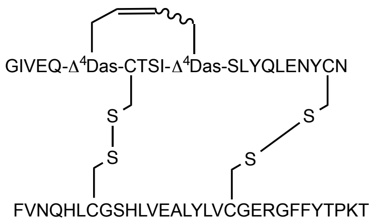

**7**	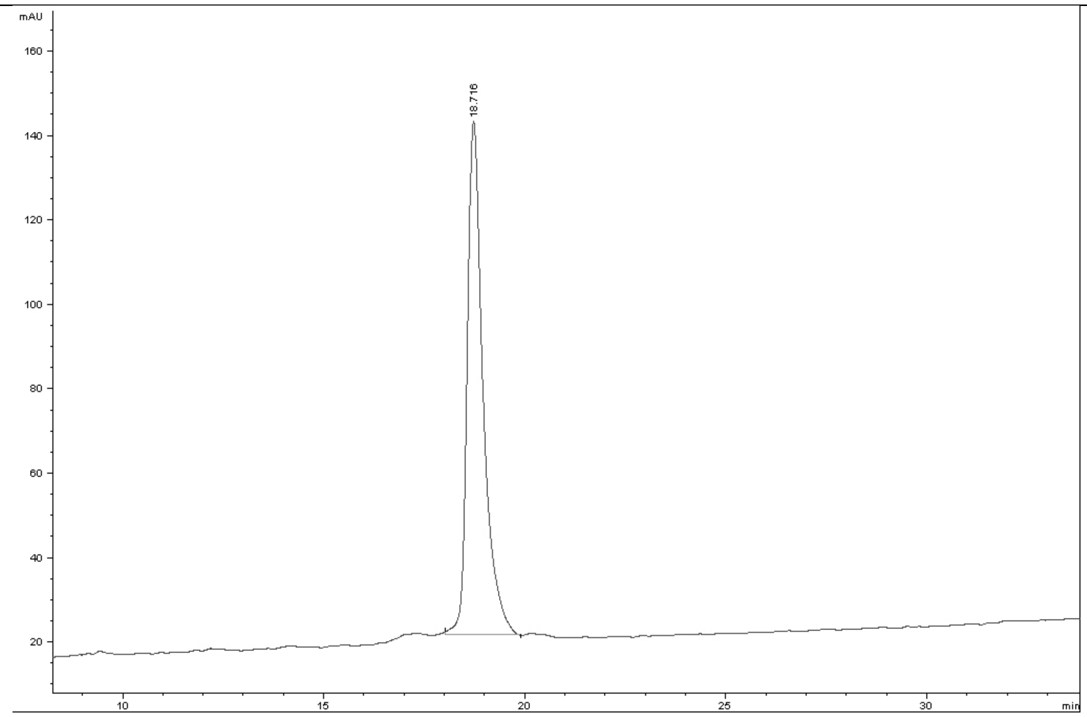	Purified peptide ***Z*-7**; t_R = 18.7 min
**7**	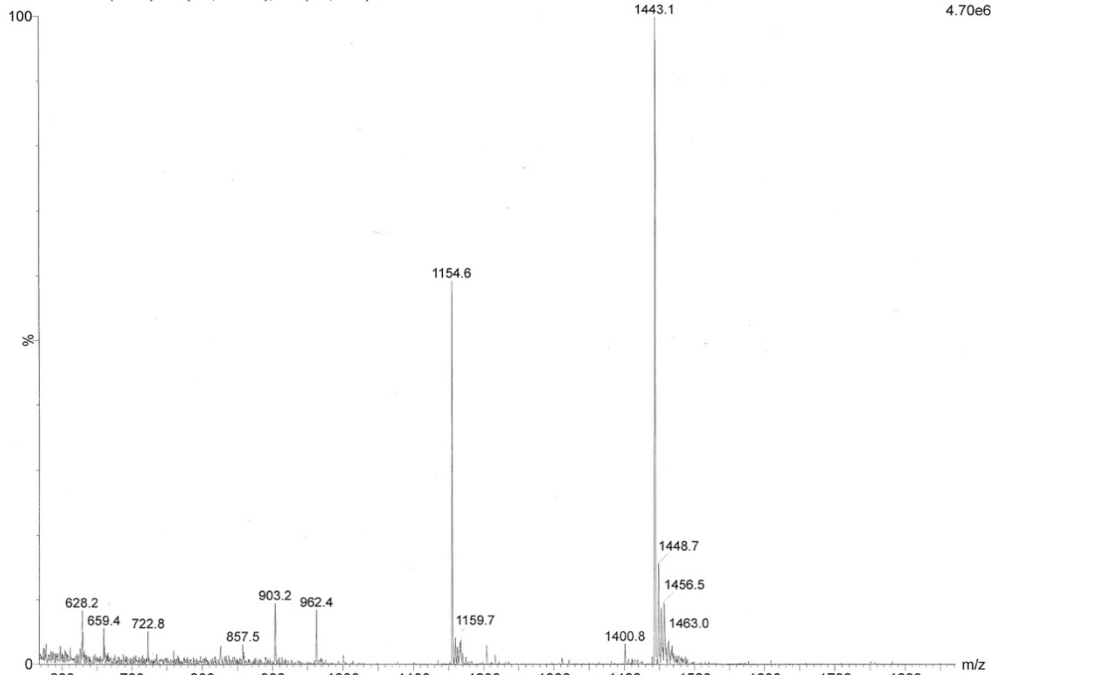	Purified ***Z*-7**

Supplementary Figure S1k

c[Δ^{4}A6,11]-Dicarba human insulin *E*-7

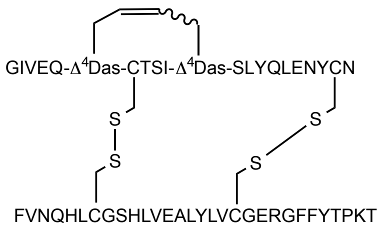

7

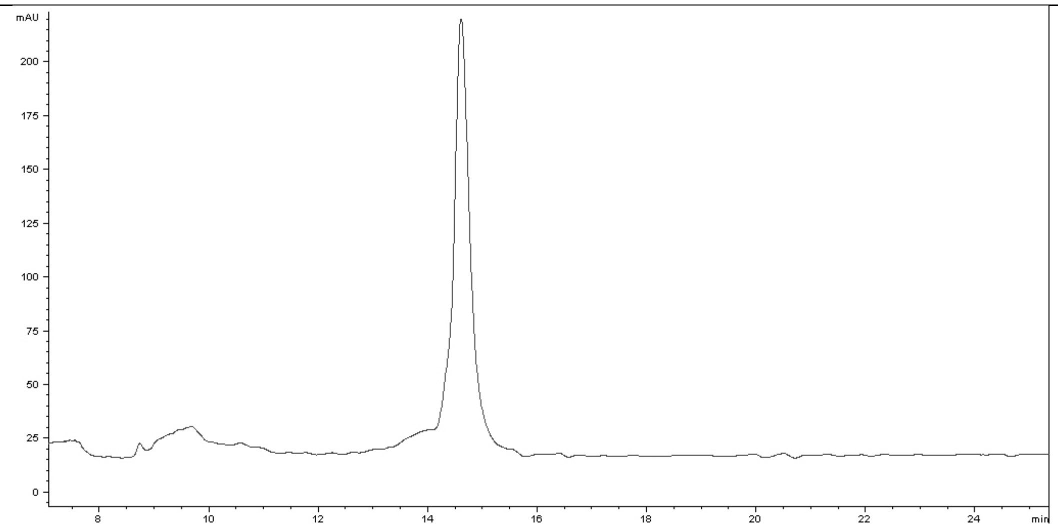

Purified peptide ***E*-7**; t_R = 14.6 min

7

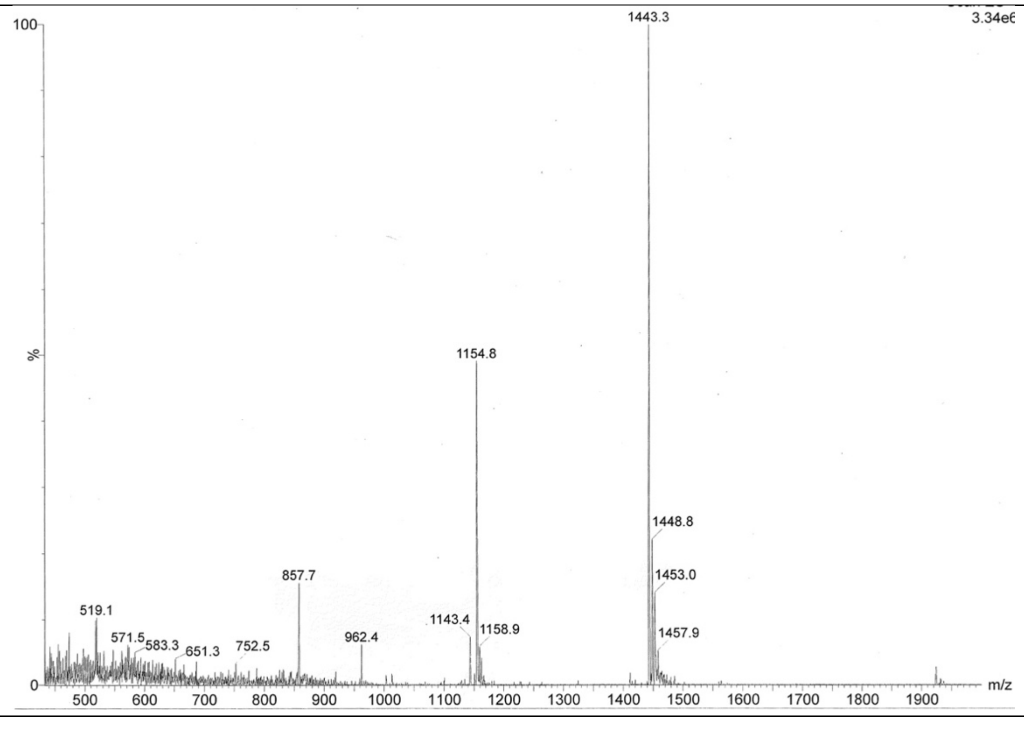

Purified peptide ***E*-7**

Supplementary Figure S1l

c[Δ^4A6,11]-Dicarba-[A7]-Cys(^{t}Bu)-[A20]-Cys(Acm) human insulin A-chain 3 *via Cis*-S1

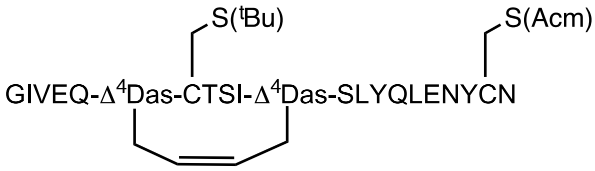

**3.1**	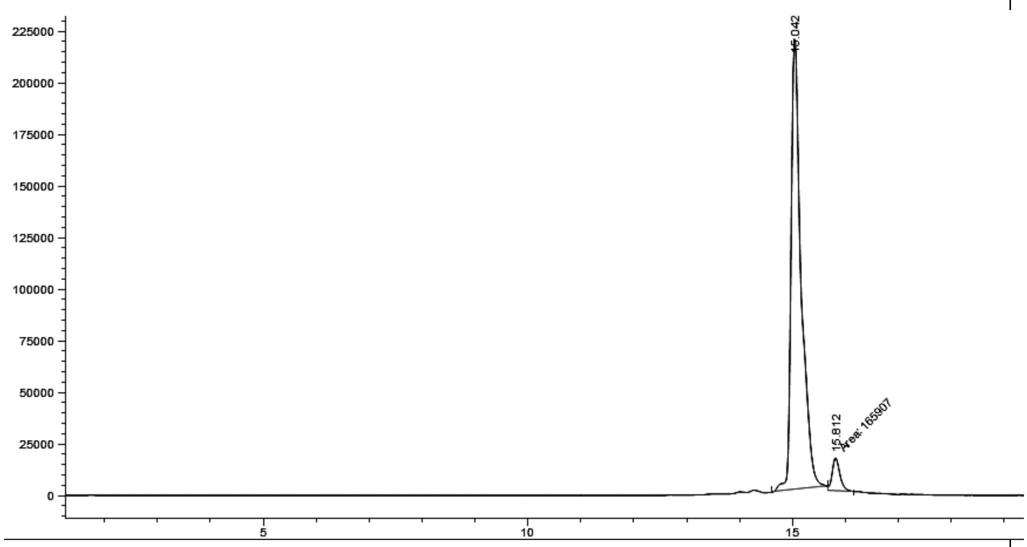	A-chain **3** synthesised using *Cis*-**S1**; t_R = 15.0 (***Z*-3**) and 15.8 (***E*-3**), *25:1.*
**3.1**	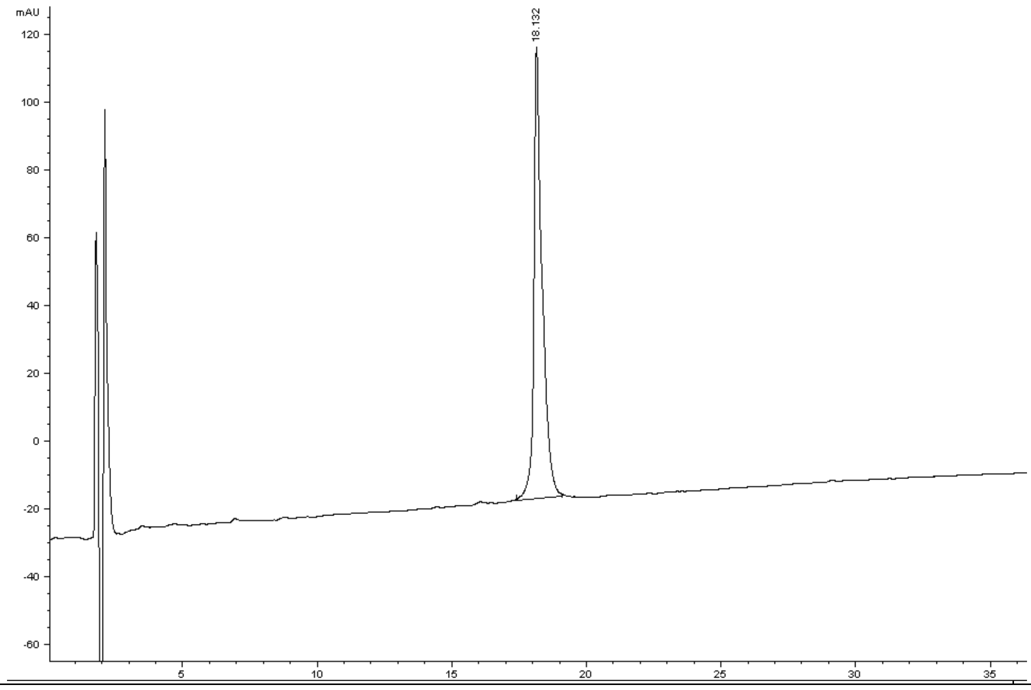	Co-injection of A-chain **3** (t_R = 15.0 (above)) with *cis*-isomer ***Z*-3** acquired *via* RCM method

Figure S1. Mass spectrum (ESI+, MeCN : H2O : TFA) monitoring synthesis of dicarba insulins.

Supplementary Figure S2

a

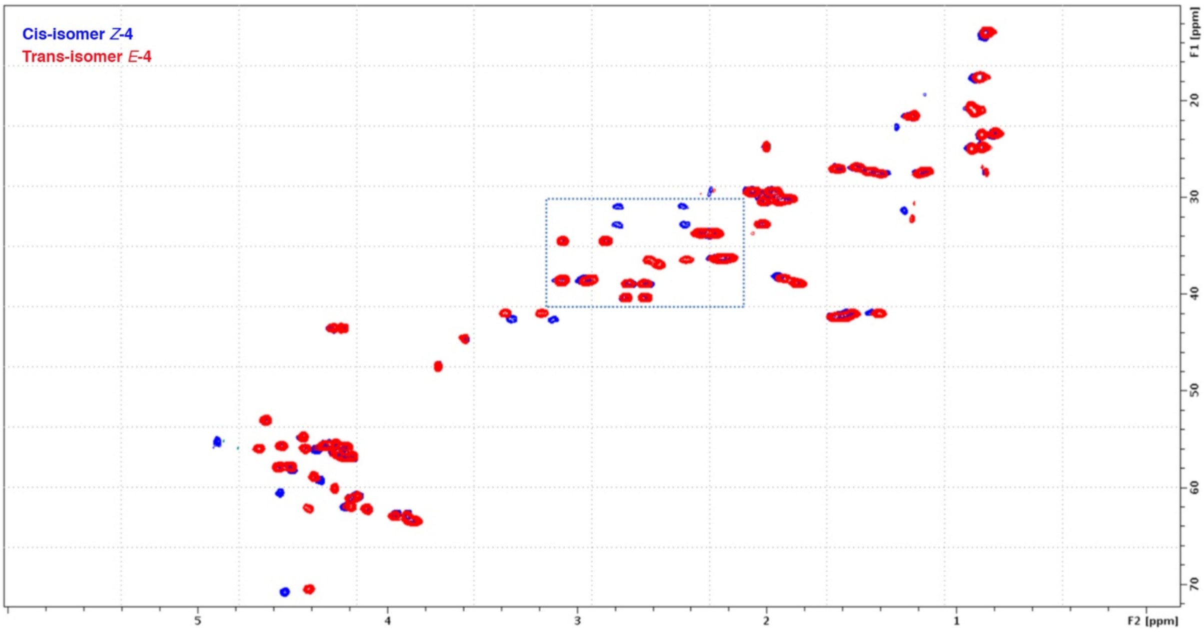

b

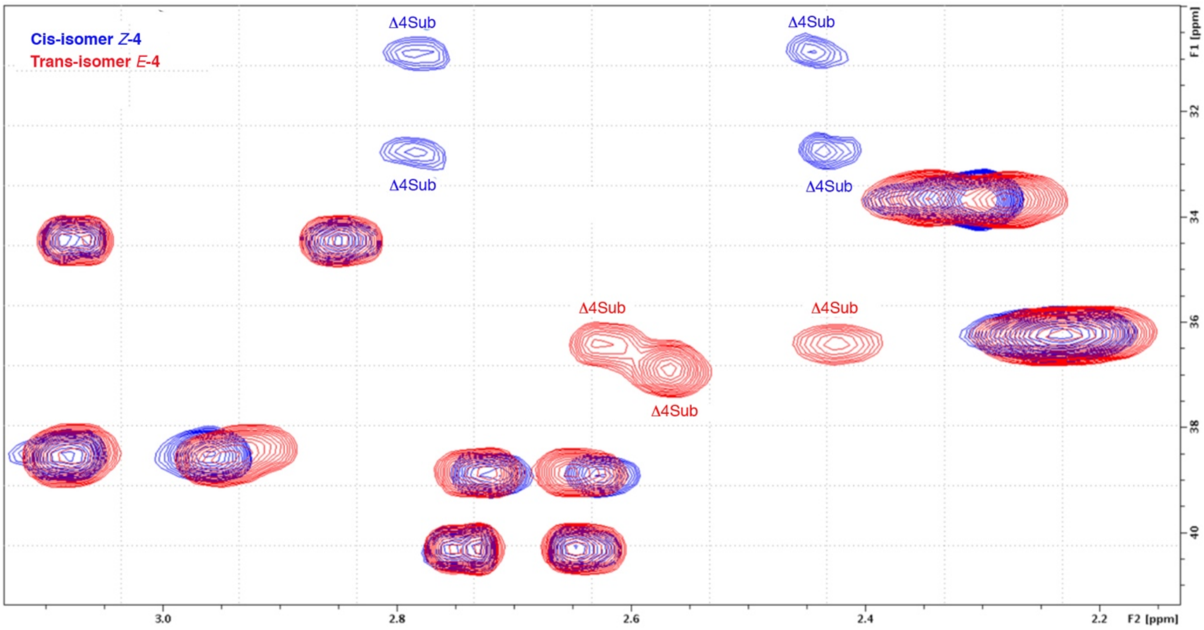

Figure S2a. ^{13}C-HSQC spectra of *cis* dicarba (blue, ***Z*-4**) and *trans* dicarba (red, ***E*-4**) insulin A chain isomers. Differences in Δ^{4}Sub C^β chemical shift are highlighted by the box. **b:** Crop of ^{13}C-HSQC spectra showing the difference between Δ^{4}Sub C^β chemical shifts of *E*- and *Z*-isomers of the dicarba insulin A chain **4**.

Supplementary Figure S3

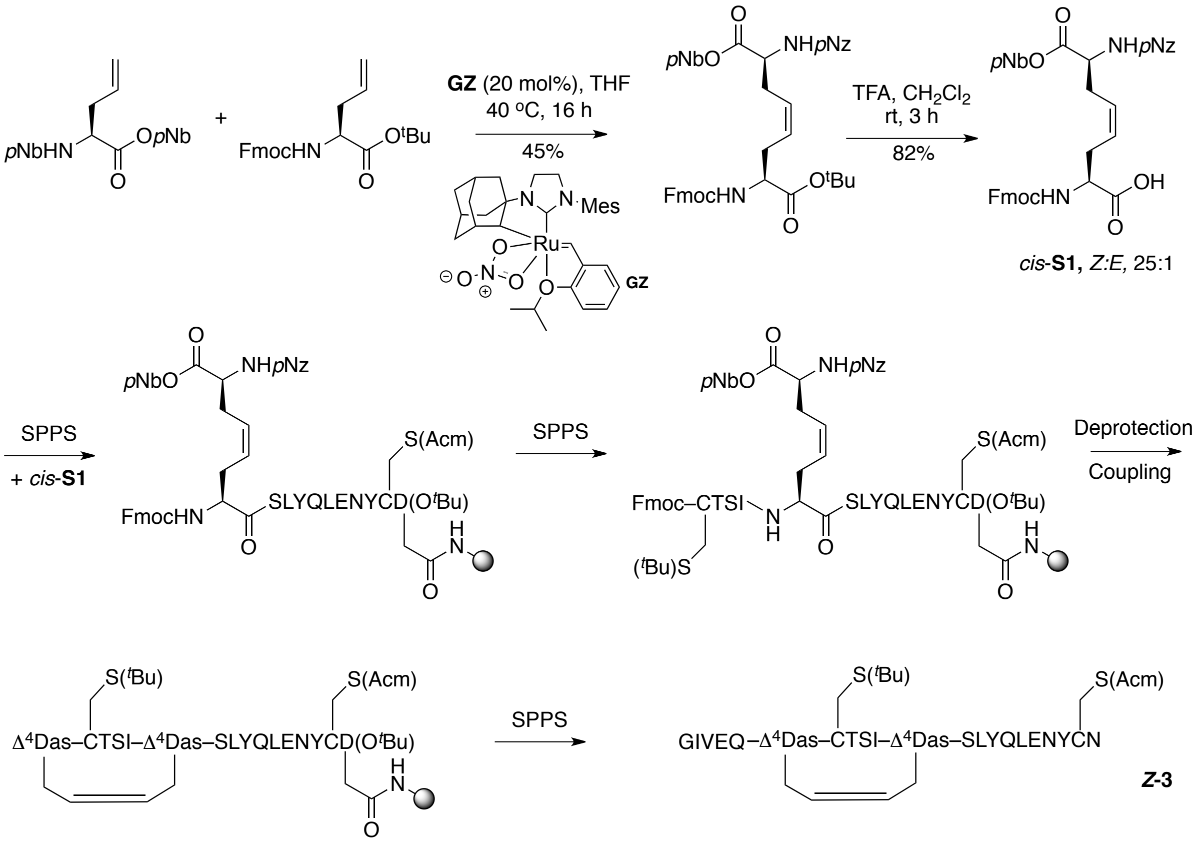

Figure S3. Stereochemical synthesis of insulin A-chain peptide *Z*-**3** using a preformed *Z*-configured, orthogonally protected Δ^4-diaminosuberic acid residue, *cis* **S1**.

Supplementary Figure S4

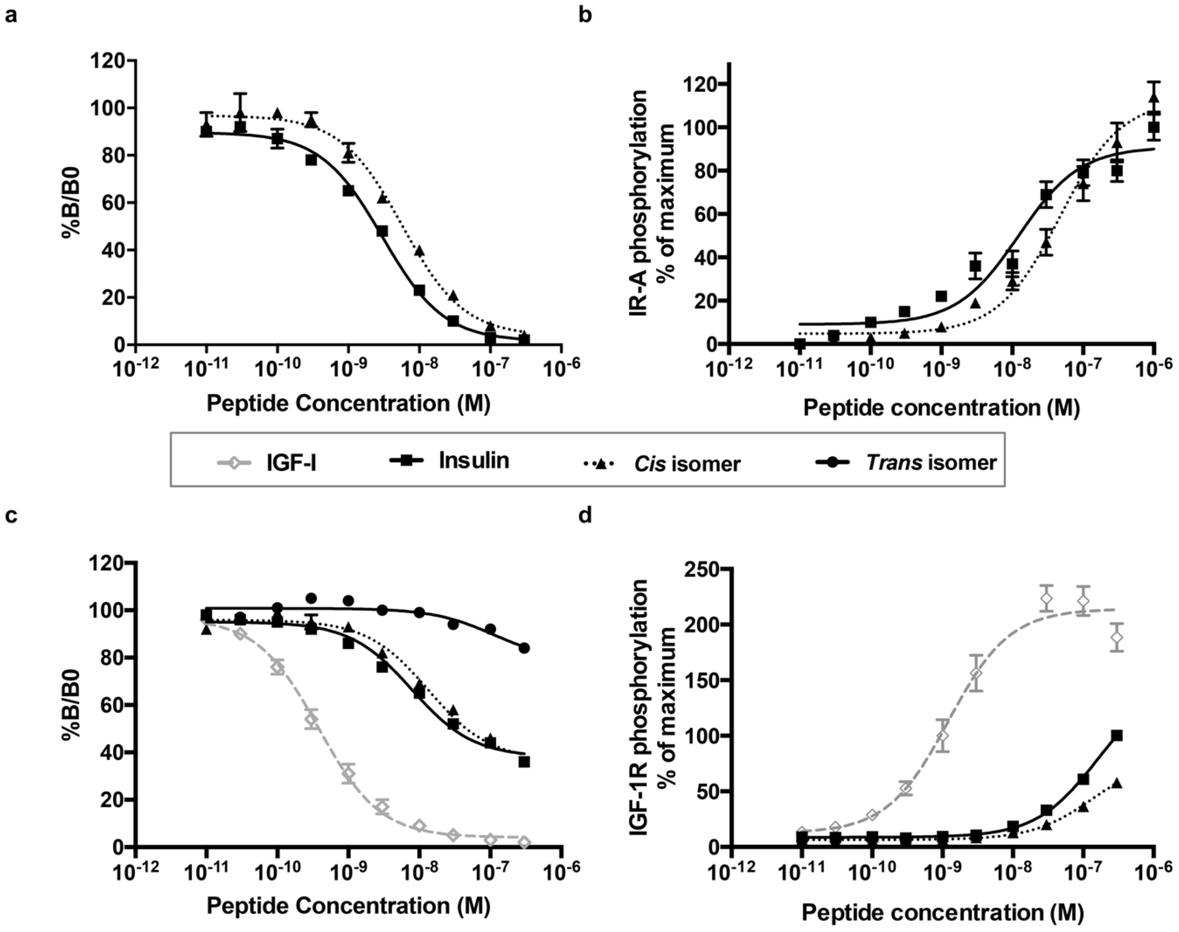

Figure S4. Insulin receptor isoform A and IGF-1R binding and activation activities of *cis-* and *trans* isomers. (**a**) Competition binding of insulin (squares) and *cis* isomer (triangles) with europium-labelled insulin. Results are expressed as a percentage of binding in the absence of competing ligand (%B/B0). (**b**) Activation of IR-A by increasing concentrations of dicarba insulins (10 min stimulation) is expressed as receptor phosphorylation as a percentage of the maximal phosphorylation induced by insulin. (**c**) Competition binding of IGF-I (diamonds), insulin (squares), *cis-* (triangles) and *trans* (circles) isomers with europium-labelled IGF-I. Results are expressed as a percentage of binding in the absence of competing ligand (%B/B0). (**d**) Activation of IGF-1R by increasing concentrations of dicarba insulins (10 min stimulation) is expressed as receptor phosphorylation as a percentage of the maximal phosphorylation induced by insulin. All data are the mean ± S.E.M. n = at least 3 independent experiments.

Supplementary Figure S5

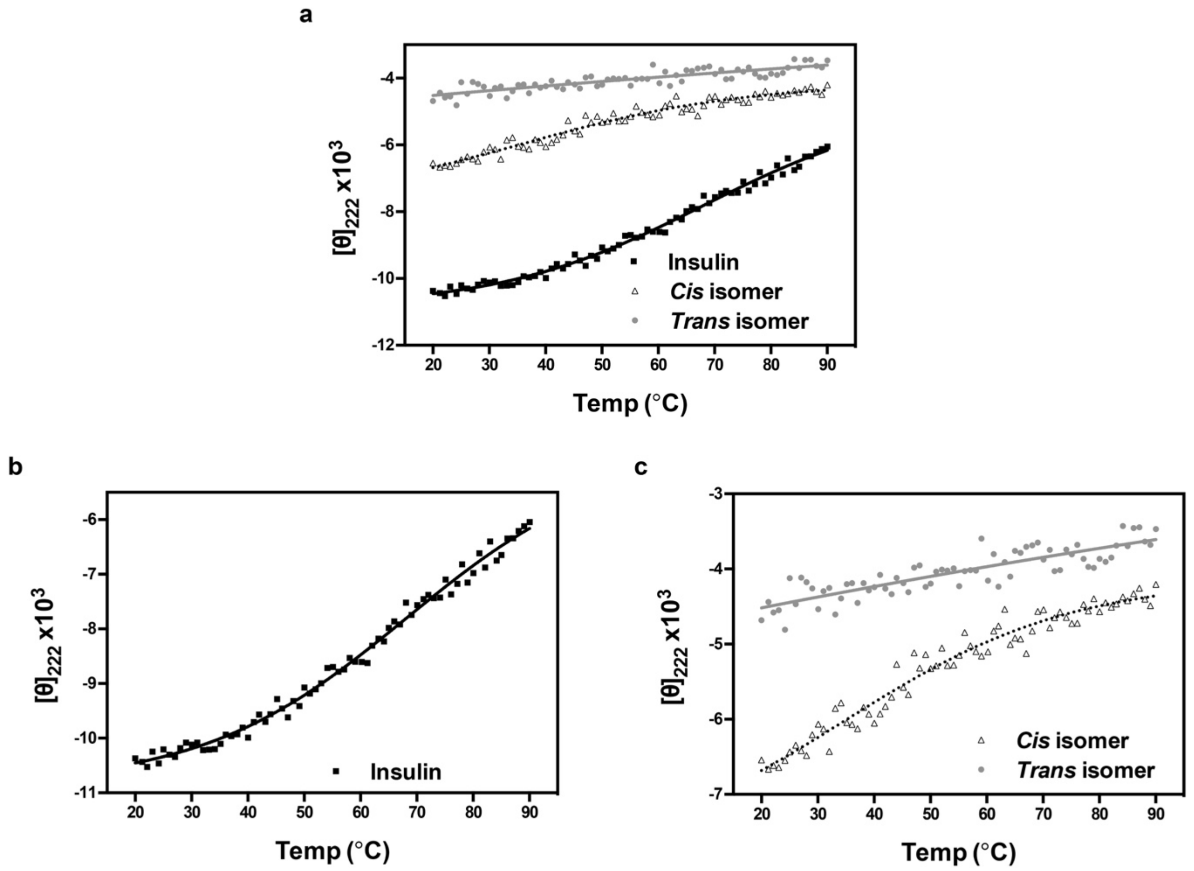

Figure S5. Thermal stability of dicarba insulin isomers in detail. (**a**) Circular dichroism far-UV spectra reveal lower helical propensities in both *cis*- and *trans* isomers. θ = ellipticity. Differences in thermal unfolding are monitored by ellipticity at 222 nm and show *trans* isomer is less stable than *cis* isomer. (**b**) and (**c**) are plotted on expanded scales to show detail.

Supplementary Figure S6

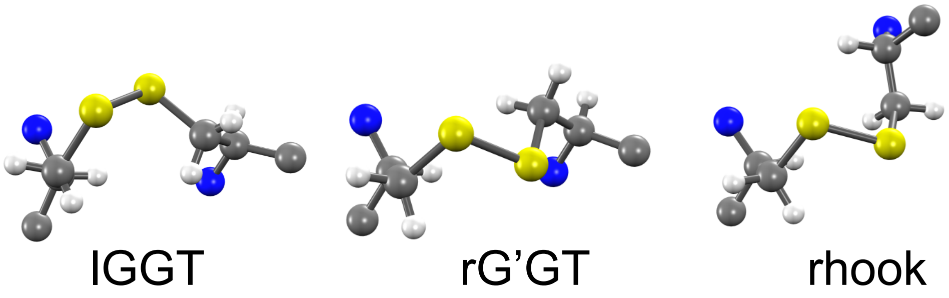

Figure S6. Schematic diagrams of the different disulfide bond conformations present in insulin structures listed in **Supplementary Table S3**. lGGT = left-handed GGT, rG’GT = right-handed G'GT, rhook = right-handed hook.

Supplementary Figure S7

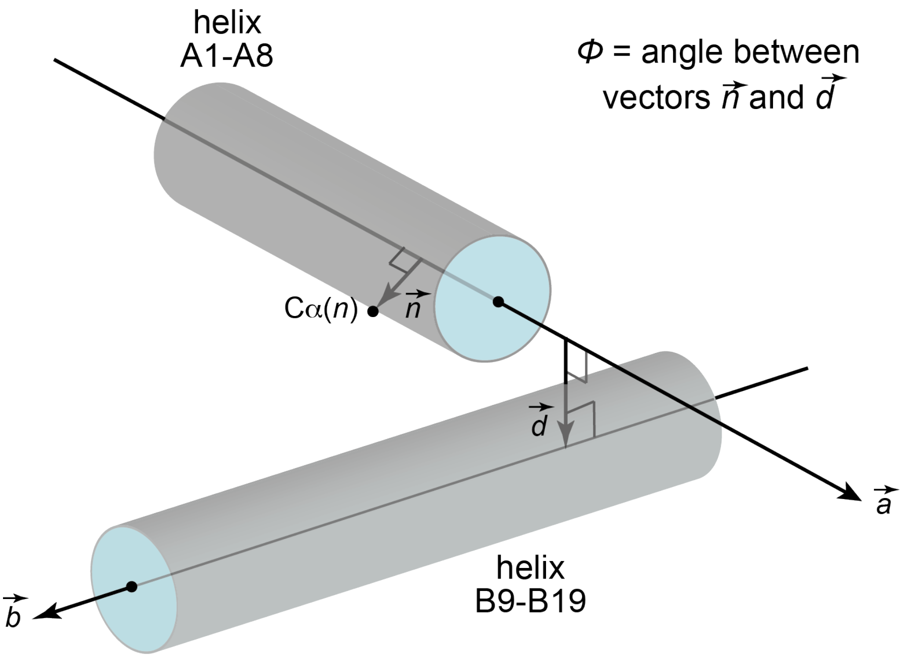

Figure S7. Definition of the azimuth angle ϕ of residue *n* within the segment A1-A10. The helix axial vectors $\vec{a}$ and $\vec{b}$ their vector of closest approach $\vec{d}$ were computed using HELIXANG within the CCP4 suite.[15]

Supplementary Movie 1

This movie highlights the difference in sidechain orientation of A1-A3 residues when transitioning between the tight and wide helical states of the first A-chain helix.

References

1 Robinson, A. J. & van Lierop, B. J. Insulin analogues. *Patent US20120225811.*, (2011).
2 Van Lierop, B. J., Bornschein, C., Jackson, W. R. & Robinson, A. J. Ring-closing metathesis in peptides – the sting is in the tail! *Aust. J. Chem.* **64**, 806-811, (2011).
3 van Lierop, B. J. *et al.* Dicarba alpha-conotoxin Vc1.1 analogues with differential selectivity for nicotinic acetylcholine and GABAB receptors. *ACS Chem. Biol.* **8**, 1815-1821, (2013).
4 Gleeson, E. C. *et al.* Stereoselective synthesis and structural elucidation of dicarba peptides. *Chem. Commun. (Camb)* **52**, 4446-4449, (2016).
5 Bullesbach, E. E. & Schwabe, C. Synthesis and conformational analysis of the insulin-like 4 gene product. *J. Pept. Res.* **57**, 77-83, (2001).
6 AMBER 14 (University of California, San Francisco, 2014).
7 Maier, J. A. *et al.* ff14SB: Improving the Accuracy of Protein Side Chain and Backbone Parameters from ff99SB. *J. Chem. Theory Comput.* **11**, 3696-3713, (2015).
8 Bayly, C. I., Cieplak, P., Cornell, W. & Kollman, P. A. A well-behaved electrostatic potential based method using charge restraints for deriving atomic charges: the RESP model. *J. Phys. Chem.* **97**, 10269-10280, (1993).
9 Dupradeau, F.-Y. *et al.* The R.E.D. tools: advances in RESP and ESP charge derivation and force field library building. *Phys. Chem. Chem. Phys.* **12**, 7821-7839, (2010).
10 R.E.D. Python: Object oriented programming for Amber force fields (Université de Picardie - Jules Verne, Sanford Burnham Prebys Medical Discovery Institute, 2013).
11 Vanquelef, E. *et al.* R.E.D. Server: a web service for deriving RESP and ESP charges and building force field libraries for new molecules and molecular fragments. *Nucleic Acids Res.* **39**, W511-W517, (2011).
12 Frisch, M. J. Gaussian 09, revision d.01. (Gaussian, Inc., Wallingford CT, 2009).
13 Roe, D. R. & Cheatham, T. E., 3rd. PTRAJ and CPPTRAJ: Software for Processing and Analysis of Molecular Dynamics Trajectory Data. *J. Chem. Theory Comput.* **9**, 3084-3095, (2013).
14 Kabsch, W. & Sander, C. Dictionary of protein secondary structure - pattern-recognition of hydrogen-bonded and geometrical features. *Biopolymers* **22**, 2577-2637, (1983).
15 Winn, M. D. *et al.* Overview of the CCP4 suite and current developments. *Acta Crystallogr. D. Biol. Crystallogr.* **67**, 235-242, (2011).
